# Impact of Additive Hydrophilicity on Mixed Dye-Nonionic Surfactant Micelles: Micelle Morphology and Dye Localization

**DOI:** 10.1021/acs.langmuir.4c00012

**Published:** 2024-04-19

**Authors:** Wenke Müller, Weronika Sroka, Ralf Schweins, Bernd Nöcker, Jia-Fei Poon, Klaus Huber

**Affiliations:** † Science Division/Large Scale Structures Group Institut Laue-Langevin 71 Avenue des Martyrs, Grenoble 38000, France; ‡ Basic Research & Technology Development KAO Germany GmbH Pfungstädter Straße 98-100, Darmstadt 64297, Germany; § European Spallation Source Box 176, Lund SE-221 00, Sweden; ∥ Food Technology, Engineering and Nutrition Lund University Box 117, Lund SE-221 00, Sweden; ⊥ Fakultät für Naturwissenschaften/Physical Chemistry Universität Paderborn Warburger Straße 100, Paderborn 33098, Germany

## Abstract

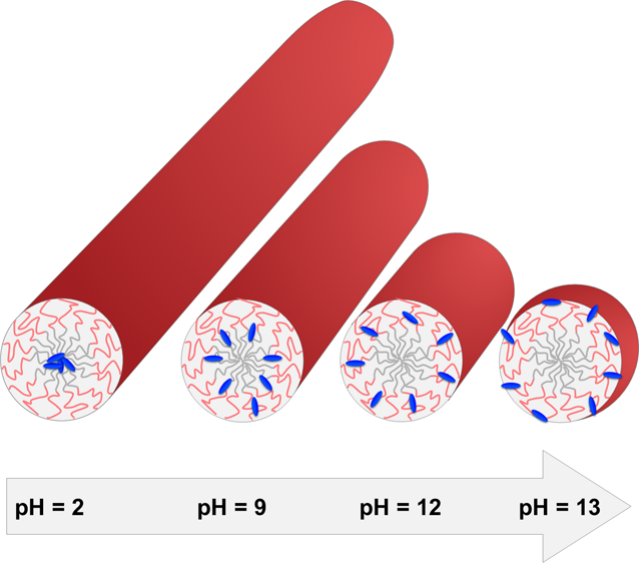

The nonionic surfactant pentaethylene glycol-monododecylether C_12_E_5_ forms micelles in aqueous solutions with a lower critical solution temperature. This characteristic solution behavior of C_12_E_5_ is independent of the pH. Such micelles are used to solubilize a large variety of active guest molecules like for instance dyestuffs. An example is an acidic azo dye termed Blue used as a hair colorant. Depending on the pH, Blue gradually changes its hydrophilicity from the protonated BlueH at pH = 2 to the bivalent anion Blue^2–^ at pH = 13 while keeping the shape and size of Blue essentially unchanged. These features of C_12_E_5_ and Blue offer the unique chance to investigate the sole impact of a tunable hydrophilicity of a guest molecule on the solution behavior of mixed micelles of the guest and C_12_E_5_. Accordingly, the present work establishes a phase diagram of Blue-C_12_E_5_ micelles and analyzes their morphology including the spatial distribution of Blue in the micelles as a function of the hydrophilicity of Blue. Small angle neutron scattering reveals the size and shape of the micelles, and detailed contrast matching of the C_12_E_5_ supported by ^1^H NMR with NOESY provided insight into the localization of Blue within the micelles as its hydrophilicity changes.

## Introduction

Alkyl ethoxylate surfactants C_*n*_E_*m*_ with *n* alkyl chain carbon atoms and *m* ethylene glycol (EG) groups are an integral part of industrial and personal consumer products.^1−3^ A common feature of these surfactants in aqueous solution is the occurrence of a liquid–liquid phase separation. The temperature at which the solution phase separates into a surfactant-rich and a surfactant-poor phase depends on the composition of the solution^3,4^ and is often termed “cloud point” or clouding temperature (CT). It corresponds to the boundary between the monophasic and biphasic regions forming a binodal curve with a lower critical solution temperature (LCST).^5^

Generally, the CT of C_*n*_E_*m*_ solutions increases with increasing hydrophilicity, i.e., with an increasing number of EG groups *m* at constant alkyl chain length, and decreases with increasing fraction of the hydrophobic part, i.e., with increasing alkyl chain length *n* if *m* is kept constant.^3,6−8^ Because of the interest in morphological transitions that micelles undergo upon approaching CT from the one-phase region, C_*n*_E_*m*_ solutions were intensively investigated with scattering techniques throughout the last decades.^9−13^ Scattering curves were interpreted assuming a combination of micellar growth and attractive interactions between individual C_*n*_E_*m*_ micelles.^12,13^

Typical applications of nonionic C_*n*_E_*m*_ surfactants aim at the solubilization and release of additives in solution like for instance dyes in a dyeing liquor. Such additives may interfere with both micellar growth and interactions between micelles, causing a change in the CT of C_*n*_E_*m*_ solutions.^14^

Corti et al. suggested that morphological transitions of C_*n*_E_*m*_ micelles upon additive addition may be similar to morphological transitions observed upon temperature change, as both factors change the temperature-distance of a sample with a given C_*n*_E_*m*_ concentration to its CT.^11^ Additive-induced CT variations are frequently discussed in terms of the polarity or hydrophilicity of the additive. Polar or hydrophilic additives that interact favorably with both EG and water increase the CT, whereas nonpolar or hydrophobic additives decrease the CT of C_*n*_E_*m*_ solutions.^14^

Although the polarity of a molecule or ion is related to its corresponding hydrophilicity, the two properties are not necessarily proportional to each other. This is nicely illustrated by examples such as carbon dioxide or chloride anions, with both having (partial) charges but with both lacking a net dipole moment. We therefore prefer to use the term hydrophilicity as one feature that induces variations of CT of C_*n*_E_*m*_.

Aside from molecular hydrophilicity, suitable accommodation of additives depends on steric effects. Vicente et al.^5^ recently investigated the impact of surface-active ionic liquids on the CT of solutions of the nonionic surfactant Tergitol. In accord with previously observed trends, they noticed a CT increase upon addition of comparably hydrophilic ionic liquids, whereas an increase of the volume fraction of the hydrophobic part of the ionic liquid lowers the CT. In contradiction to this trend, Sen et al.^15^ observed an increase of the CT of solutions containing the nonionic surfactant Triton X-100 and imidazolium-based ionic liquids as they increased the length of the hydrophobic alkyl chain attached to the imidazolium moiety. The two examples demonstrate that steric requirements and the hydrophilicity of additives both affect the solution behavior of nonionic surfactant micelles.

**Figure 1:**
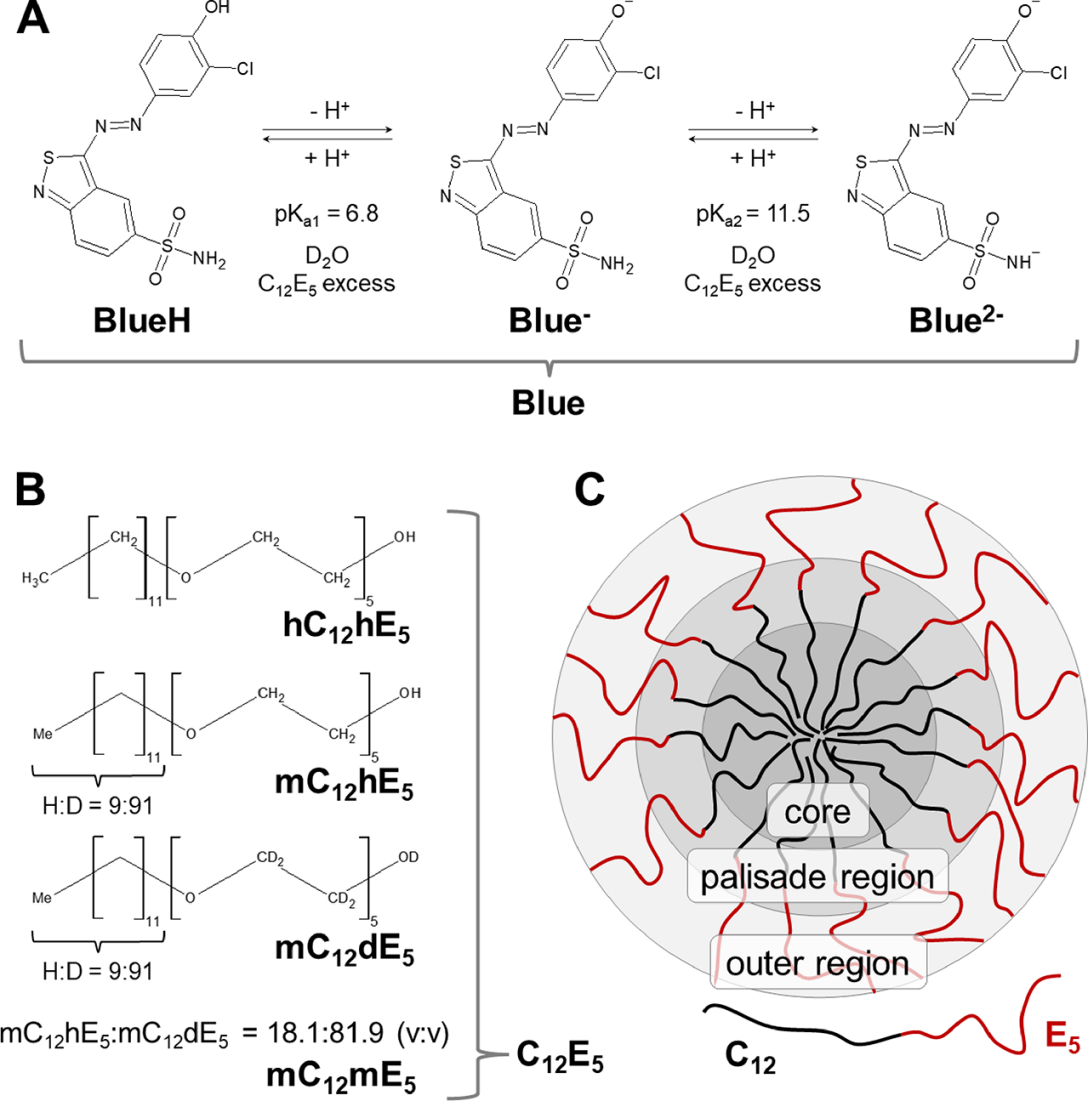
(A) Acid/base equilibria of Blue. Displayed p*K*_a_ values were measured for an excess of C_12_E_5_ in an isotonic solution of NaCl in D_2_O at room temperature (22 °C). As D_2_O was used exclusively to prepare the solvent, the term pD replaces pH throughout the Results and Discussion section. (B) Nonionic surfactant C_12_E_5_ and isotopically substituted species used in the present work. H:D denotes the ratio of hydrogen to deuterium in the statistically deuterated alkyl chain and V:V the volume ratio of (mC_12_hE_5_):(mC_12_dE_5_) at the SANS contrast match point. (C) General classification of the C_12_E_5_ micellar regions according to their polarity.^16^

The impact of both effects, steric effects due to variable chemical constitution of the additive and the effect of additive hydrophilicity, can be separated if hydrophilicity could be varied in one and the same molecule/ion. Herein, the azo dye Blue, an acidic additive, is presented as an example, where any steric effect can be totally removed, making possible an investigation of the impact of pure variation of molecular hydrophilicity of a host on the solution behavior of *C_n_E_m_*. Blue possesses two p*K*_a_ values between pH = 0 and 14 and therefore makes three different additive hydrophilicities accessible by simply varying the pH (Figure 1A).

Our approach is further facilitated by the use of C_12_E_5_ as a representative surfactant of the C_*n*_E_*m*_ class in the present study. As is demonstrated in our work, C_12_E_5_ and its micelles are completely insensitive to the solution pH. A scheme of the surfactant and its micelles is shown in Figure 1B,C.

It is this unique chance to isolate the impact of additive hydrophilicity on the solution behavior of C_12_E_5_ micelles and the resulting Blue-C_12_E_5_ coassemblies that motivates our work. Accordingly, the objective of the present study is to fundamentally understand the effect of additive hydrophilicity free of any steric effects on the additive–surfactant interactions leading to variations in the CT of such solutions and in the morphology of the comicelles in such solutions. The objective is pursued by addressing two issues: The first issue deals with the impact of Blue on the phase behavior and the morphology of Blue-C_12_E_5_ coassemblies. The variation of CT is recorded at variable hydrophilicity (i.e., at variable pH) for a given composition in solution, and along with this, the morphological changes of comicelles are measured by small angle neutron scattering (SANS). The second issue addresses the spatial distribution of Blue within the coassemblies at variable pH by the use of contrast matching SANS.

## Experimental Methods

### Chemicals and Sample Preparation

Blue (HC Blue 18, ≥ 99.8%) was provided by KAO Germany GmbH, Germany. Hydrogenated pentaethylene glycolmonododecylether (hC_12_hE_5_, BioXtra, ≥98.0% GC) was obtained from Sigma-Aldrich Chemie GmbH, Germany. C_12_E_5_ with statistical deuteration of the alkyl tail at a hydrogen/deuterium ratio of 9:91 and either hydrogenated (mC_12_hE_5_) or deuterated (mC_12_dE_5_) pentaethylene glycol headgroup were synthesized by the DEMAX laboratory ESS in Sweden (SI, Section SI11) to be able to contrast match C_12_E_5_ with D_2_O (SI, Section SI8). NaCl (puriss. p.a., ≥99.5%) was obtained from Sigma-Aldrich Chemie GmbH, Germany. D_2_O (99.9 atom % D) was obtained from Sigma-Aldrich Chemie GmbH, Germany. The 7.6 M DCl solution in D_2_O (99% D) used to prepare the DCl solution for pD adjustment was obtained from Euriso-top, France. The 40 wt % NaOD solution in D_2_O (99.5 atom % D) used to prepare the NaOD solution for pD adjustment was obtained from Aldrich Chem. Co., US. As all samples were prepared in D_2_O, dissociation leads to D^+^ + OD^–^, and pD is used instead of pH except for the Abstract, Introduction, and Conclusions, where pH is used for better.^17,18^

Figure 1B shows the C_12_E_5_ surfactant employed in this research. As contrast matching in SANS was needed to localize Blue in C_12_E_5_ micelles, C_12_E_5_ was used in different degrees of deuteration. Therefore, Figure 1B also provides an overview of the employed isotopically substituted C_12_E_5_ species. Figure 1C shows the classification of different regions in a surfactant micelle according to their polarity.^16^

Samples were prepared by mixing the alkaline solvent with stock solutions of Blue and C_12_E_5_ at the required ratio and subsequently adjusting the pD. The solvent contained NaCl at a concentration of 154 mM and NaOD at a concentration of 20 mM. D_2_O was used to prepare the solvent in all cases. Stock solutions with [Blue] = 10 mM or [C_12_E_5_] = 75 mM were prepared by dissolving Blue or C_12_E_5_ in the solvent and gently mixing the solution for 1 to 2 h. Samples containing [Blue] = 12.5 mM were prepared by weighing the appropriate amount of Blue and subsequently adding the solvent and C_12_E_5_ stock solution at the required ratio followed by gently mixing the solution for 1 to 2 h. All other samples were prepared by mixing of stock solutions and solvent. Following this preparation, the pD of each sample was adjusted at room temperature (22 °C) using a ∼1 M solution of NaOD in D_2_O and a ∼1 M solution of DCl in D_2_O. Samples were stored at a temperature lower than the measurement temperature (usually 4–7 °C) for a minimum of 24 h prior to measurement and equilibrated at the measurement temperature for at least 1 h prior to measurement except for NMR measurements, where the equilibration time was 15 min. Samples for NMR spectroscopy were filtered (MACHEREY-NAGEL, CHROMAFIL Xtra H-PTFE syringe filters, pore size 0.2 μm) into NMR tubes at a temperature of 7 °C after an equilibration time of at least 4 h at that temperature and were subsequently stored for a minimum of 24 h at 7 °C. Samples for the match point determination with SANS were not filtered. However, the solvent used for preparing stock solutions and the DCl solution used for pD adjustment were filtered (MACHEREY-NAGEL, CHROMAFIL Xtra H-PTFE syringe filters, pore size 0.2 μm). Samples for all other SANS experiments were not filtered either but prepared from the stock solutions, solvent, NaOD solution and DCl solution, which were filtered (MACHEREY-NAGEL, CHROMAFIL Xtra H-PTFE syringe filters, pore size 0.2 μm) at room temperature.

### Determination of Phase Diagrams

For the determination of phase diagrams, samples were prepared as described above and stored at a temperature of 4 °C for 24 h before further analysis. Samples had a volume of 1.2 mL and were prepared in 4 mL glass vials. The as-prepared samples were placed in an incubator (model IL 68R, VWR, Belgium). Starting from 4 °C, the sample temperature was increased in increments of 1 K, and the samples were equilibrated for 1 h and inspected for the onset of phase separation at each temperature. The upper limit of 50 °C corresponds to the maximum temperature of the used incubator model. The temperature at which clouding occurs for a given sample composition will be termed “clouding temperature” (CT) in the following to avoid confusion with the cloud point defined by the ASTM standard test method ASTM D2024-09(2017) for 1 wt % surfactant solutions.^19^

### Small-Angle Neutron Scattering

Samples for small-angle neutron scattering (SANS) were prepared as described above. Three different SANS experiments were performed: (1) The first experiment corresponds to the match point determination experiment, which served to determine the ratio between mC_12_hE_5_ and mC_12_dE_5_ at which no form factor scattering is observed (match point). (2) In the second experiment, “full contrast” SANS curves were recorded from samples containing the completely hydrogenated surfactant hC_12_hE_5_ and Blue. (3) In the third experiment, C_12_E_5_-matched SANS curves were recorded, where samples contained mC_12_hE_5_ and mC_12_dE_5_ at the match point determined in step (1) and Blue. As C_12_E_5_ was matched out under these conditions, the *q*-dependent scattering signal only arose from Blue.

SANS was performed at the small-angle neutron scattering instrument D22 at the Institute Laue-Langevin (Grenoble, France). A circular neutron beam with a diameter of 13 mm and cuvettes with a path length of 2 mm were used. The D22 instrument possesses two detectors: a front detector, which was at a fixed distance of 1.4 m to the sample, and a rear detector. Measurements were carried out at two sample-to-rear-detector distances: 4 m (collimation 4 m) and 17.6 m (collimation 17.6 m) at a neutron wavelength of 6 Å to cover a *q*-range of 0.0026 to 0.6424 Å^–1^. Neutrons were detected with two ^3^He detectors (multitube detector) consisting of vertically aligned Reuter–Stokes tubes, with 128 tubes for the rear and 96 tubes for the front detector, all with a diameter of 8 mm and a pixel size of 8 mm × 8 mm. Detector images were corrected to the transmission of the direct beam, scaled to absolute intensity, and azimuthally averaged, and experimental resolution was considered using the GRASP software.^20^ Empty cells and solvent scattering were subtracted from the scattering curves.^21^

In all cases, samples were equilibrated at the measurement temperature of 10 °C in the SANS cuvettes overnight. For match point determination and C_12_E_5_-matched experiments, 404-QS quartz cuvettes with a path length of 2 mm (Hellma, Müllheim, Germany) were used. For full contrast measurements, 404-QX quartz cuvettes with a path length of 2 mm (Hellma, Müllheim, Germany) were used.

The determination of the surfactant composition (based on components given in Figure 1B) where the scattering contrast of C_12_E_5_ matches that of the solvent is outlined in detail in Section SI8 of the Supporting Information and yielded a volume ratio V/V of Φ(mC_12_hE_5_)/Φ(mC_12_dE_5_) = 18.1:81.9 for the match point.

### NMR Spectroscopy

Samples for NMR spectroscopy were prepared, filtered, and stored as described above. Samples were equilibrated in the NMR spectrometer at 10 °C for 15 min before measurement. ^1^H NMR and nuclear Overhauser effect (NOESY) spectra were recorded with a Bruker 600 MHz NMR spectrometer. ^1^H NMR chemical shifts were referenced to residual HDO.^22^

## Theoretical Models

SANS curves were fitted using the SASfit software package.^23^

### Intermicellar Arrangements

The scattering intensity in a SANS experiment can be described by^24^
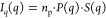
1which is strictly valid only for dispersions of spherically symmetric objects. In eq 1, *n*_p_ is the number density of colloidal particles, *q* the magnitude of the scattering vector, and *P*(*q*) is the single particle form factor. The form factor in eq 1 is given in units of the scattering cross section [cm^2^].^25,26^*S*(*q*) is the structure factor, which describes interparticle correlations.

Structure factors *S*(*q*) exist for hard spheres,^27,28^ for spheres with attractive potentials (“Baxter” model),^29^ and for spheres with repulsive potentials (Hayter and Penfold).^30^ In the present work, the use of structure factors based on simple hard spheres sufficiently well reproduced *S*(*q*) of repulsive interactions among spherical micelles even if they are loaded with negatively charged dyestuff ions.^27,28^ Attractive interactions as they are observed among micelles approaching the phase boundary in an LCST system generate concentration fluctuations of the number density of micelles close to the phase transition threshold.^11−13,31,32^ In such a case, the scattering intensity in the low-*q* regime can be described using the Ornstein–Zernike relation:^13^
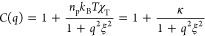
2in place of *S*(*q*) of eq 1. In eq 2*k*_B_ is the Boltzmann constant, *T* the temperature, and χ_T_ the isothermal osmotic compressibility. The numerator *n*_p_*k*_B_*T*χ_T_ is denoted as the scaling factor κ. The value of ξ corresponds to the correlation length of local concentration fluctuations.

### Form Factor Models

Scattering data on C_*n*_E_*m*_ solutions were typically interpreted in terms of (polydisperse) cylinders.^10,12,13^ Based on cryogenic transmission electron microscopy (cryo-TEM) images, Bernheim-Groswasser et al.^33^ suggested the coexistence of spherical and rather short worm-like micelles in C_12_E_5_ solutions at temperatures sufficiently far away from the CT. SANS curves emerging from solutions of C_*n*_E_*m*_ micelles closer to the CT showed deviations from the anticipated form factor of rod-like aggregates at low *q*, which could be attributed to an elongational growth of cylindrical micelles toward wormlike micelles^33−36^ or concentration fluctuations as described by eq 2.^11−13,37^ Accordingly, the following form factors or simplifications thereof shall be used in the present work: (i) core–shell spheres with *r*_core_ and *r*_shell_ as core radius and shell thickness, respectively, and uniform scattering length densities η_core_ and η_shell_ of the core and shell, respectively (*P*_core–shell-sphere_), (ii) core–shell cylinders with *r*_core_ and *r*_shell_ as core radius and shell thickness, respectively, and uniform scattering length densities η_core_ and η_shell_ of the core and shell, respectively, and with cylindrical caps on both ends (*P*_core–shell-cylinder_); (iii) core–shell spheres with a log-normal distribution of core radii *r*_core_ and a linear decay of the scattering length density of the shell from that of the core to that of the solvent (*P*_core-linshell-sphere_); (iv) core–shell cylinders with a log-normal distribution of core radii *r*_core_ and a linear decay of the scattering length density of the shell from that of the core to that of the solvent (*P*_core-linshell-cylinder_). The flexibility of the cylinders was neglected in all cases, which was justified by the short length values observed in all cases. The coexistence of cylinders and spheres was not considered either. Both simplifications are applied to keep the number of parameters low in particular once *S*(*q*) or *C*(*q*) has to be considered. An overview of the form factors and their applications is given in Table S1 of the Supporting Information.

## Results and Discussion

### Clouding Temperatures of Blue-C_12_E_5_ Solutions

**Figure 2:**
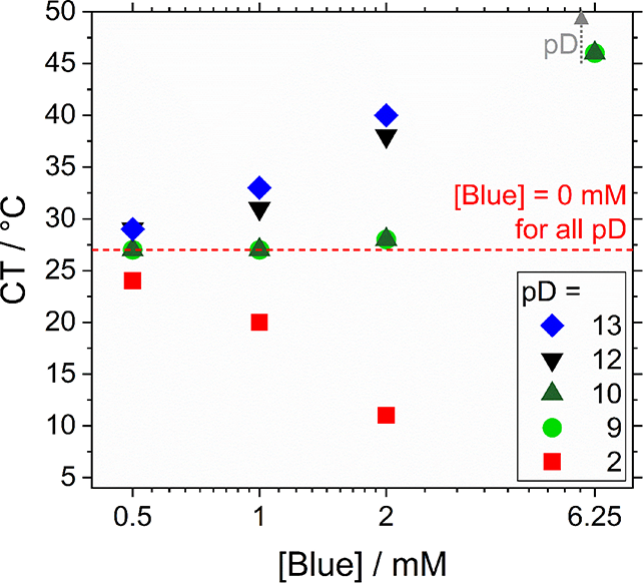
CT of solutions containing the surfactant hC_12_hE_5_ at a concentration of [hC_12_hE_5_] = 25 mM and varying concentrations of Blue at different pD values. An isotonic NaCl solution (*I* = 0.154 M) in D_2_O served as the solvent. The pD was adjusted by addition of DCl or NaOD. For [Blue] = 12.5 mM, solution CT lays outside the observed temperature range, i.e., above 50 °C for pD ≥ 9. The same is true for [Blue] = 6.25 mM at pD ≥ 12. The latter observation is indicated by a gray dotted arrow. C_12_E_5_ solutions containing [Blue] ≥ 6.25 mM at pD = 2 phase separated at all observed temperatures.

The phase behavior of Blue-C_12_E_5_ micelles is described by means of the clouding temperatures (CT). Figure 2 displays CTs of solutions containing Blue and the nonionic surfactant hC_12_hE_5_ at variable Blue concentrations in comparison to the CT of pure hC_12_hE_5_ solutions for different solution pD values. As explained in the experimental part and Figure 1B, the abbreviation hC_12_hE_5_ refers to the nondeuterated version of C_12_E_5_. Values of the pD are used instead of pH because the solvent was always prepared in D_2_O.

Although clear assignment of the CT values to either the binodal or spinodal curve of the phase diagram is not possible, data were determined with the same method and are thus suitable for a consistent comparison of their trends.

First and foremost, the CT of solutions containing only hC_12_hE_5_ does not depend on solution pD, thereby excluding pD-responsive behavior of the hC_12_hE_5_ surfactant. Changes in the CT of Blue-hC_12_hE_5_ solutions are therefore solely attributed to the presence of Blue at its given concentration and in its given state of hydrophilicity (BlueH, Blue^–^, or Blue^2–^) determined by the respective pD of the solution. Table 1 provides an overview of dissociation states at variable pD values based on experimentally determined p*K*_a_ values. The determination of these p*K*_a_ values is outlined in the Section SI1 of the Supporting Information.

**Table 1:** Position of the Acid/base Equilibrium Shown in Figure 1 for Different pD Values Based on Experimentally Determined pK_a_ Values^a^

pD	*x*(BlueH)/mol %	*x*(Blue^–^)/mol %	*x*(Blue^2–^)/mol %
2 ± 0.1	100 ± 0	0 ± 0	0 ± 0
9 ± 0.1	0.3 ± 0.1	99.5 ± 0.2	0.2 ± 0.1
10 ± 0.1	0 ± 0	96.9 ± 0.8	3.1 ± 0.8
12 ± 0.1	0 ± 0	24.0 ± 5	76.0 ± 5
13 ± 0.1	0 ± 0	3.1 ± 0.8	96.9 ± 0.8

a The determination of p*K*_a_ values is outlined in the SI and was performed in an isotonic solution of NaCl in D_2_O and at high C_12_E_5_ excess at room temperature. The variable *x* denotes the mole fraction of each species in the acid/base equilibrium. Uncertainties in that mole fraction, which could be induced through an experimental error of the pD to an extent of ±0.1, are given as well.

As shown in Figure 2, CTs follow the trend expected for variations in additive hydrophilicity.^14^ Addition of the poorly water-soluble BlueH at pD = 2 causes a significant decrease in the CT of C_12_E_5_ solutions, which is already observed for a [BlueH]/[C_12_E_5_] ratio as low as 1:50, corresponding to the sample containing [BlueH] = 0.5 mM in Figure 2. At pD = 9, the CT is only slightly increased for low concentrations of Blue, with the increase getting stronger with increasing [Blue^–^]. At a concentration of [Blue^–^] = 12.5 mM, the expected CT already lays outside the observed temperature range. At pD = 12, a mixture of Blue^–^ and Blue^2–^ is present, resulting in an effect intermediate between pD = 9 and 13, which can be related to the Blue^–^/Blue^2–^ ratio in such samples. At pD = 13, Blue is deprotonated twice, which results in a greater increase of solution CT than at pD = 12. As Blue^2–^ is expected to be more hydrophilic than Blue^–^, the observed trend is consistent with the hydrophilicity hypothesis.^14^

To conclude this section, the addition of the poorly water-soluble BlueH to C_12_E_5_ solutions results in a reduction of their CT with respect to the pure surfactant solution. An increase of the molecular hydrophilicity, accomplished with the ionic states Blue^–^ and Blue^2–^, increases the solution CT, shifting it above the CT of pure surfactant solutions. An increase of the dyestuff concentration boosts this effect by increasing the deviation of CT from the level of the pure surfactant solution. Results are consistent with literature, which suggests an increase of the solubility of nonionic surfactant micelles and a concomitant CT increase upon addition of more hydrophilic additives.^5,14^

### Structure Analysis of Blue-C_12_E_5_ Micelles at Variable pD Values with Full Contrast SANS

As the addition of Blue changes the CT of C_12_E_5_ solutions, morphological transitions similar to temperature-induced transitions are expected for Blue-C_12_E_5_ micelles. Therefore, SANS curves emerging from solutions of Blue and C_12_E_5_ will be interpreted with models similar to models that were applied to describe the scattering from pure C_*n*_E_*m*_ solutions^13^ (see Theoretical Models and Section SI2 of the Supporting Information). Cylindrical or spherical shapes determine the choice of form factors for the C_12_E_5_ micelles and Blue-C_12_E_5_ micelles. Once concentration fluctuations are observed close enough to the CT, they are accounted for by the Ornstein–Zernike expression (eq 2).^37^ At conditions sufficiently far from the CT, repulsive interactions among micelles are observed and treated with a structure factor *S*(*q*) from simple excluded volume spheres.^27,28^

For the analysis of SANS curves emerging from Blue/C_12_E_5_ solutions, preliminary fits were first performed to assist a fitting strategy in two steps. Preliminary fits were performed on SANS curves of solutions containing [Blue] ≤ 2 mM at all pD values. For this purpose, the form factor of end-capped core–shell cylinders including concentration fluctuations through the Ornstein–Zernike expression (eq 2) was applied. The cylinder core length *L*_core_, the cylinder shell thickness *r*_shell_, the scattering length densities, and the parameters κ and ξ were fitted. Core lengths obtained from this approach were surprisingly similar and scattered around the average value of 66 Å with a standard deviation of 13 Å. The value of 66 Å was therefore used as a starting point for further analysis in step 2.

**Figure 3:**
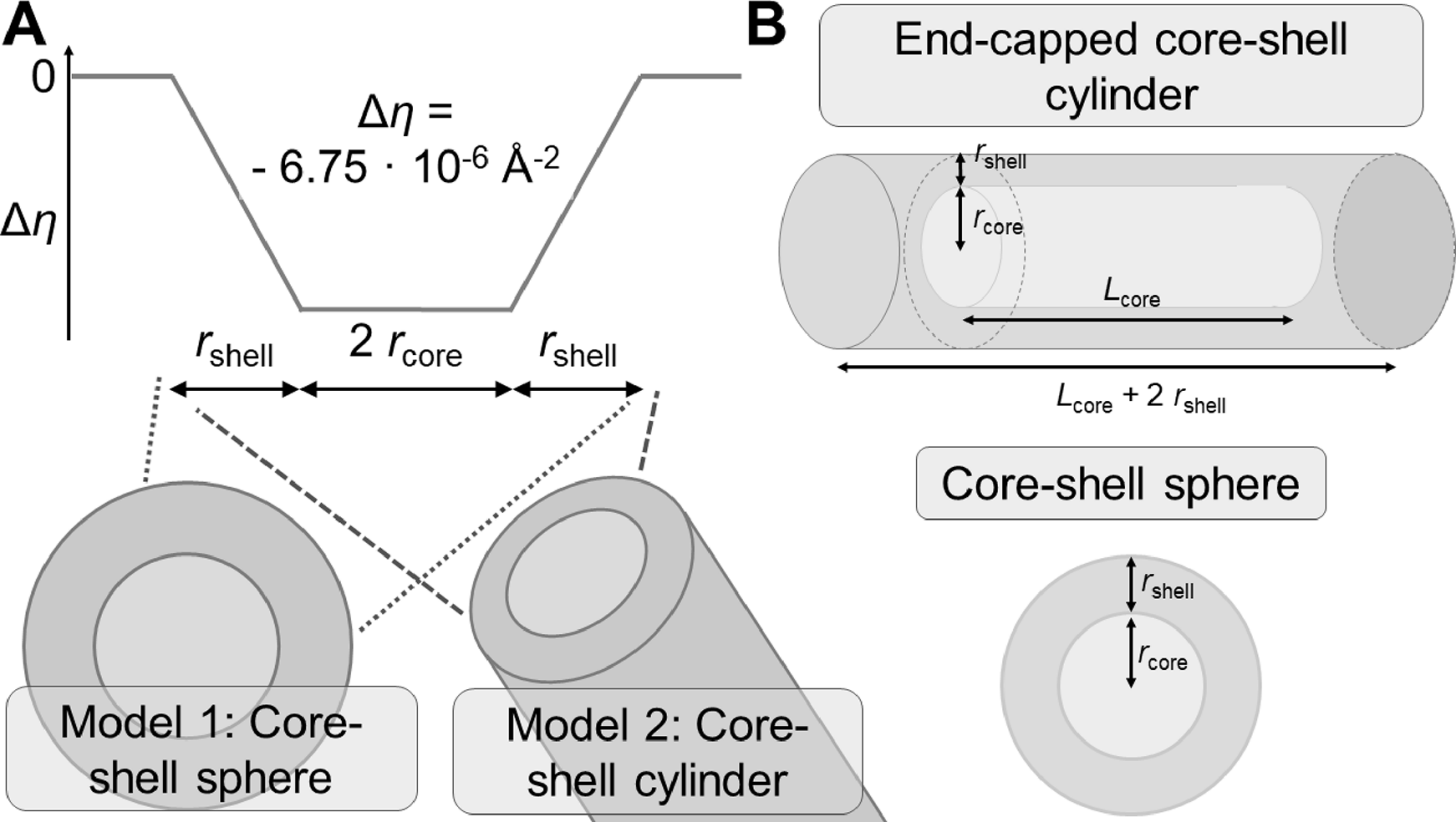
Schematic presentation of form factor models used for fitting (A) the high-*q* range (*q* > 0.045 Å^–1^) and (B) the complete *q*-range of full contrast SANS curves emerging from the solution containing Blue and hC_12_hE_5_. (A) For fitting the high-*q* range, the depicted scattering length density difference profile was assumed for the core–shell structure based on theoretical scattering length densities displayed in Table S2. (B) For fitting the entire *q*-range with the form factor of a core–shell structure, the core was assumed to possess the homogeneous scattering length density η_core_, and the shell was assumed to have a homogeneous scattering length density η_shell_.

In the first step, the average cross section of Blue-C_12_E_5_ assemblies was analyzed by fitting only the high-*q* range (*q* > 0.045 Å^–1^) with core–shell models. Within this approach, the form factor model of a core–shell sphere (*P*_core-linshell-sphere_) and the form factor model of a core–shell cylinder (*P*_core-linshell-cylinder_) were compared. Figure 3A displays the scattering length density difference profile Δη(*r*) = η(*r*) – η_solvent_ used for the cross section of these core–shell morphologies. The micellar core was assigned a homogeneous scattering length density η_core_, and the scattering length density η(*r*) in the shell region was assumed to linearly approach η_solvent_ at *r* = *r*_core_ + *r*_shell_, starting from η_core_ at the outer boundary of the core where *r = r*_core_. This leads to a net decay of Δη within the shell region. The core radius *r*_core_ was furthermore subdued to a log-normal distribution. A log-normal distribution could account for small deviations in the assumed profile of Δη(*r*) or for a coexistence of spherical and cylindrical micelles that possess different cross-section dimensions.^13,38^ Details on the choice of these models and on the best fits can be found in SI Section SI3.

Mean values for core radii were found to vary between 12 Å < *r*_core_ < 15 Å if the assumption of cylindrical micelles resulted in the best fit (Figure S4 and Tables S7-S9) and between 16 Å < *r*_core_ < 17 Å if spherical micelles led to better fits (Figure S4, Table 2, and Table S9). Standard deviations of *r*_core_, which were calculated from the log-normal distribution, lay between 2.5 Å < SD(*r*_core_) < 4 Å in both cases. Shell thicknesses *r*_shell_ varied between 10 Å < *r*_shell_ < 15 Å. None of these values show a systematic trend with pD or with concentration (Table 2 and Figure S4). Therefore, the investigation of the micellar cross section was designed to provide dimensions of the micellar core, which were used in the subsequent analysis of the entire *q*-range.

**Table 2:** Form Factor Model That Best Reproduces the Experimental Full Contrast SANS Curves in the Mid- and Low-*q* Range (*q* < 0.134 Å^–1^)^a^

	pD = 2	pD = 9	pD = 12	pD = 13
[Blue] = 0 mM	end-capped core–shell cylinder, *L*_**core**_**=** 66 Å	end-capped core–shell cylinder, *L*_core_= 66 Å	end-capped core–shell cylinder, *L*_core_= 66 Å	end-capped core–shell cylinder, *L*_core_= 66 Å
[Blue] = 1 mM	end-capped core–shell cylinder, *L*_core_= 66 Å	end-capped core–shell cylinder, *L*_core_= 66 Å	end-capped core–shell cylinder, *L*_core_= 40 Å	end-capped core–shell cylinder, *L*_core_= 40 Å
[Blue] = 2 mM	end-capped core–shell cylinder, *L*_core_= 66 Å	end-capped core–shell cylinder, *L*_core_= 66 Å	end-capped core–shell cylinder, *L*_core_= 40 Å	end-capped core–shell cylinder, *L*_core_= 40 Å
[Blue] = 6.25 mM	phase separated	end-capped core–shell cylinder, *L*_core_= 40 Å	core–shell sphere, *r*_core_= 17.1 Å, hard sphere potential	core–shell sphere, *r*_core_= 16.9 Å, hard sphere potential
[Blue] = 12.5 mM	phase separated	end-capped core–shell cylinder, *L*_core_= 40 Å	core–shell sphere, *r*_core_= 16.9 Å, hard sphere potential	core–shell sphere, *r*_core_= 16.5 Å, hard sphere potential

a In case of end-capped core–shell cylinders with a core length *L*_core_ of 20, 40, 66, 100, or 200 Å, fluctuation scattering according to the Ornstein–Zernike expression (eq 2) was taken into account in all cases. SANS curves from samples with [Blue] ≥ 6.25 mM and pD ≥ 12 were described with the form factor of core–shell spheres in combination with a structure factor *S*(*q*) taking into account hard sphere interactions. All samples contained hC_12_hE_5_ at a concentration of [hC_12_hE_5_] = 25 mM

In the second step, the whole *q*-range of full contrast SANS curves was analyzed using eq 1. For this purpose, form factors *P*_core–shell-cylinder_ of end-capped core–shell cylinders with a core length *L*_core_ of either 20, 40, 66, 100, or 200 Å and the form factor of *P*_core–shell-sphere_ of a core–shell sphere (Figure 3B) were systematically applied to all experimental SANS curves. Restriction of the regime of core lengths to 20 Å < *L*_core_ < 200 Å is justified as follows. The lower limit of 20 Å is close to core-radii obtained from step 1, and fits using the upper limit of *L*_core_ = 200 Å or longer do not describe experimental SANS curves sufficiently well. The intermediate value of *L*_core_ = 66 Å was adopted from the above-described preliminary analysis. Availability of model form factors in accessible software libraries (SasView, SASfit) predetermined the use of the form factor for end-capped core–shell cylinders with flat ends instead of hemispherical end-caps. To minimize the number of fit parameters, the core radius *r*_core_ and its distribution were fixed to the corresponding state retrieved in step 1 (Figure S4 and Tables S7–S9), and *L*_core_ was predetermined to the set of values given above. The particle number density *n*_p_ in eq 1 was calculated by dividing the total surfactant concentration by an estimated aggregation number, assuming that all C_12_E_5_ molecules participate in micelle formation. Details are given in Section SI3 of the Supporting Information. The shell thickness (*r*_shell_) and the scattering length densities of the core (η_core_) and the shell (η_shell_) were fitted. A structure factor *S*(*q*) had to be used in addition to the form factor of core–shell spheres at high pD. Fluctuation scattering was included at low pD by substituting *S*(*q*) in eq 1 with the Ornstein–Zernike expression *C*(*q*) from eq 2, leading to the scaling term κ and the correlation length ξ as fit parameters.

**Figure 4:**
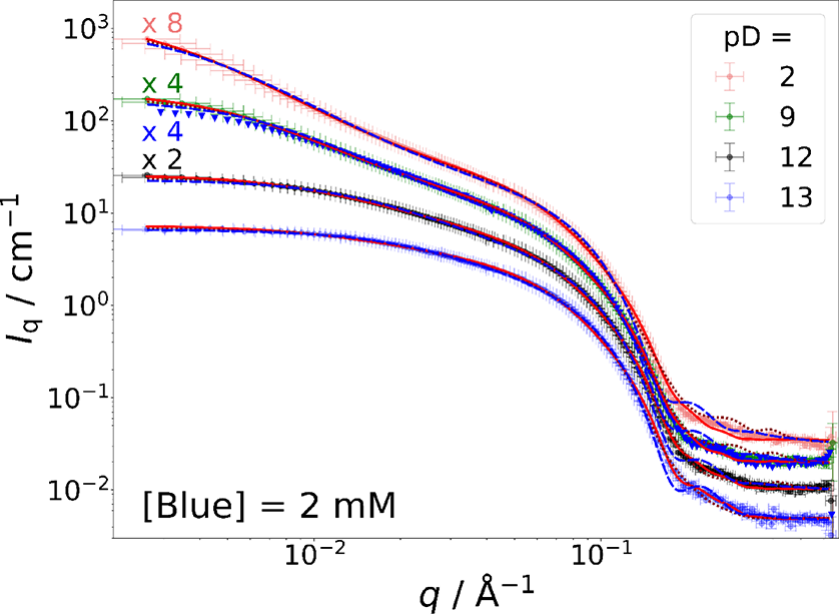
Full contrast SANS curves of solutions containing [hC_12_hE_5_] = 25 mM and [Blue] = 2 mM at variable pD values (see legend) and full contrast SANS curve of solution containing [hC_12_hE_5_] = 25 mM without Blue at pD = 2 as a reference (blue triangles). An isotonic NaCl solution (*I* = 0.154 M) in D_2_O served as the solvent. SANS curves were recorded at a sample temperature of 10 °C. Red line: fit with the form factor of end-capped core–shell cylinders with a core length of *L*_core_ = 66 Å and a hard sphere structure factor. Dark red dotted line: fit with the same model but with *L*_core_ = 40 Å. Blue dashed line: fit with the form factor of core–shell spheres and a hard sphere structure factor.

Figure 4 displays a representative set of SANS curves and fits with a selection of applied form factor models at variable pD values to illustrate the impact of solute hydrophilicity on the morphology of the comicelles. All other SANS curves are shown in Figures S5–S8.

Fits to the entire q-regime of SANS curves are compared by means of the reduced χ^2^ parameter referring to the low and mid-*q* range (*q* < 0.134 Å^–1^). This comparison is shown in Table S4. As a result thereof, Table 2 provides an overview on the model form factors that best describe the SANS curve in the range of *q* < 0.134 Å^–1^ for each sample, the core–shell cylinder, or the core–shell sphere, and Table 2 furthermore displays the core size of the best model fit.

A shrinking of assembly size toward higher pD is clearly visible. Except for pD = 2, the assembly size decreases with increasing Blue concentration. If correlated with the clouding temperature expressed as the distance Δ*T*_CT_ of a given solution state to the respective CT, the micellar size tends to get larger the closer to the CT that sample lies. It must be noted that all measurements were performed at the same temperature and a variation in Δ*T*_CT_ is caused by a shift of the clouding temperature curve.

SANS curves of samples containing [Blue] ≥ 6.25 mM and pD ≥ 12 could not be described with cylinder models. The form factor of a core–shell sphere was thus used instead to describe those SANS curves. Corresponding fits are shown in Figures S7 and S8. Fits are significantly improved upon inclusion of a structure factor according to eq 1 derived for a simple hard sphere excluded volume interaction potential.^27,28^ The resulting averaged excluded volume distances occur between 34 and 40 Å and are close to but larger than the overall radii obtained for the respective spherical micelles (*r*_core_ + *r*_shell_ ≈ 29 Å).

Using the core lengths (*L*_core_) displayed in Table 2 and the core radii (*r*_core_) obtained by the fit strategy described above (Figure S4 and Tables S7–S9), core volumes of C_12_E_5_ micelles can be calculated. This permits an estimation of aggregation numbers based on the assumption that the micellar core only consists of C_12_ alkyl chains with an approximate volume of 350.2 Å^3^.^39^ As core radii of cylindrical micelles do not vary with *L*_core_, *N*_agg_ of micelles was calculated to be *N*_agg_ = 100 ± 6, and 65 ± 7 for micelles with *L*_core_ = 66 and 40 Å, respectively. For spherical micelle, an average *N*_agg_ = 56 ± 4 was calculated.

**Figure 5:**
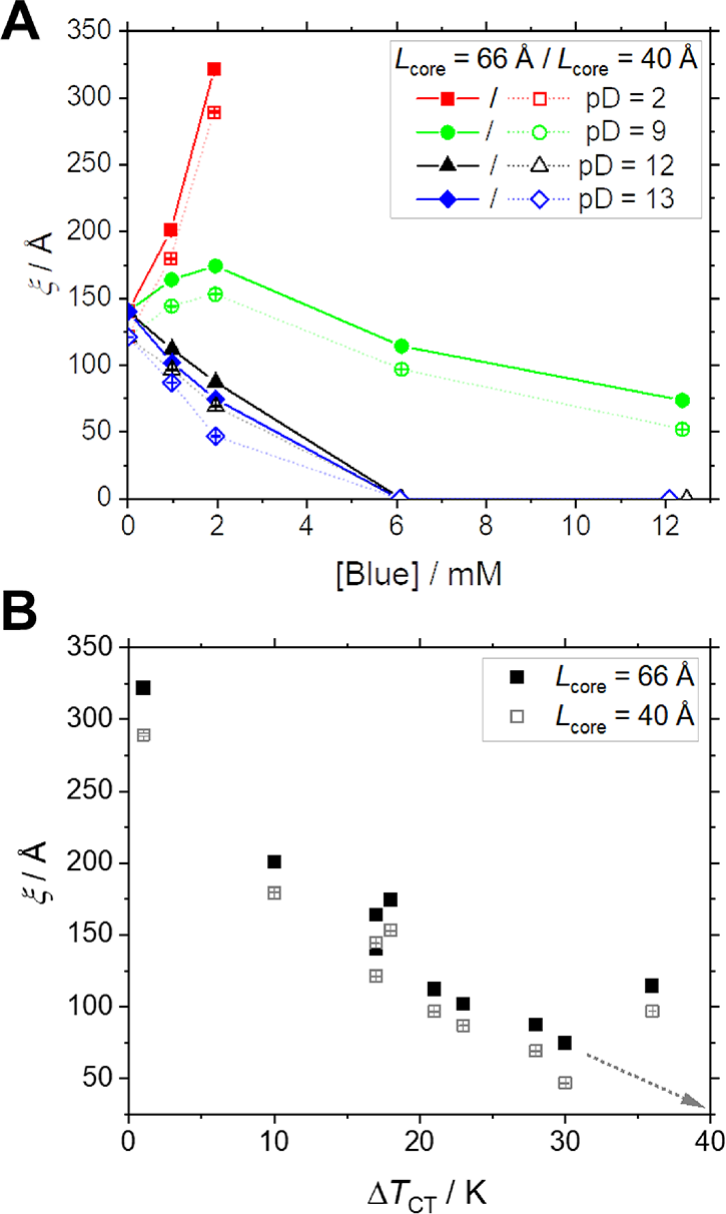
(A) Correlation length of concentration fluctuations ξ obtained from fitting full contrast SANS curves with the Ornstein–Zernike expression combined with the form factor of end-capped core–shell cylinders with a core length of either *L*_core_ = 66 or 40 Å versus the concentration of Blue at the given pD at [hC_12_hE_5_] = 25 mM. An isotonic NaCl solution prepared in D_2_O served as the solvent, and the sample temperature was 10 °C. (B) The same values of ξ as a function of the temperature difference Δ*T*_CT_ between the sample temperature and the CT of that sample, independent of sample composition. The gray dotted arrow indicates that no Ornstein–Zernike fluctuations were observed for most samples with Δ*T*_CT_ > 40 °C.

Figure 5A displays correlation lengths of concentration fluctuations (ξ) obtained from the fits for the cases where the model of core–shell cylinders has been superior to the model of core–shell spheres. The trend for ξ is similar for both choices of a core length, i.e., for either *L*_core_ = 66 or 40 Å. At pD = 2, ξ strongly increases with increasing concentration of Blue. This indicates strong attractive interactions between micelles if they contain neutral BlueH and is therefore consistent with the decrease of solution CT upon addition of BlueH. In line with this, ξ decreases upon addition of Blue as pD increases. At pD ≥ 9, micelles incorporate negatively charged Blue molecules, which lower attractive interactions. Eventually at [Blue] ≥ 6.25 mM and pD ≥ 12, ξ becomes zero, signaling the disappearance of attractive interactions. The respective samples ([Blue] ≥ 6.25 mM and pD ≥ 12) even show repulsive intermicellar interactions.

A suitable way to discuss trends of the correlation length ξ is to correlate it with the deviation of the actual temperature of the sample (10 °C) from its CT (Δ*T*_CT_). As is shown in Figure 5B, ξ decreases with the temperature distance from the CT and does not depend on sample composition. As the measurement temperature was 10 °C and CTs were only observed up to a temperature of 50 °C, the maximum observable Δ*T*_CT_ amounts to 40 K. Data of five samples with Δ*T*_CT_ > 40 K fall beyond this limit. As expected, the respective correlation lengths are zero or close to zero, i.e., ξ = 0 for four samples and ξ = 52 or 74 Å (at *L*_core_ = 40 or 66 Å, respectively) for the fifth sample.

To summarize, cylindrical C_12_E_5_ micelles were shown to shrink upon increasing the hydrophilicity of the dye molecules. Changing the dye from BlueH via Blue^–^ to Blue^2–^ transforms rod-like micelles eventually to spherical micelles in case of Blue^2–^. This change of micellar morphology is accompanied by a variation in solvent behavior. The analysis of SANS curves from samples containing C_12_E_5_ at a concentration of [C_12_E_5_] = 25 mM and at a constant temperature of 10 °C confirms that the addition of Blue changes both the size of micelles and their intermicellar interactions. Addition of BlueH significantly increases attractive interactions between micelles, whereas the addition of Blue^–^ or Blue^2–^ weakens those intermicellar attractions. This effect is more pronounced when the negative charge is increased from Blue^–^ to Blue^2–^. Blue^2–^ even induces repulsive intermicellar interactions among the micelles at [Blue^2–^] ≥ 6.25 mM. The trends of attractive intermicellar interactions can be correlated with the temperature distance from the CT of a sample. An interesting question to be tackled in what follows is if such morphological transformations accompanied by a change in solubility can be correlated with the spatial distribution of dye molecules within the hosting micelles.

### Localization of Blue in C_12_E_5_ Micelles with NMR Spectroscopy

**Figure 6:**
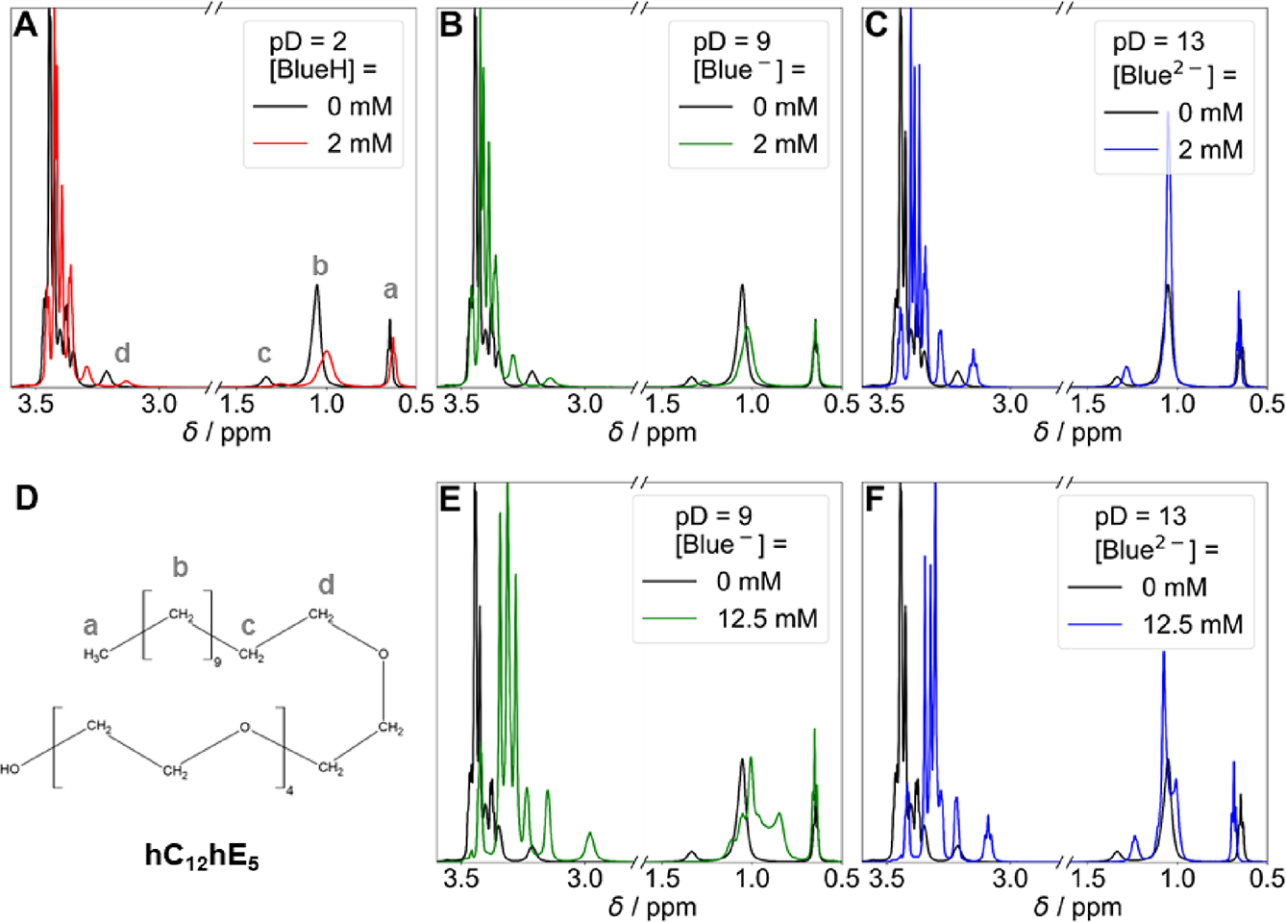
hC_12_hE_5_ resonance region in ^1^H NMR spectra of solutions containing Blue and [hC_12_hE_5_] = 25 mM at variable Blue concentrations and pD values compared to the ^1^H NMR spectrum of a pure surfactant solution (black line). An isotonic NaCl solution (*I* = 0.154 M) in D_2_O served as the solvent. All measurements were performed at 10 °C. The first row shows hC_12_hE_5_ proton resonances of solutions containing [Blue] = 2 mM, and the second row displays spectra recorded from solutions containing [Blue] = 12.5 mM. No NMR spectrum could be recorded for the solution containing [Blue] = 12.5 mM and [hC_12_hE_5_] = 25 mM at pD = 2 because of phase separation. Panel D displays the assignment of ^1^H NMR resonances of the pure hC_12_hE_5_ spectrum in panel A to protons of the hC_12_hE_5_ molecule.^9,41,46^ Resonances between 3.3 and 3.5 ppm in the pure surfactant spectrum belong to protons of the E_5_ headgroup.^9,41,46^

Changes in chemical shift and peak width of surfactant ^1^H-resonances upon Blue addition provide first insights into the solubilization locus of Blue in hC_12_hE_5_ surfactant micelles.^40−44^Figure 6 shows the hC_12_hE_5_ resonance region of ^1^H NMR spectra recorded from solutions containing Blue and hC_12_hE_5_ in comparison to the spectrum of a pure hC_12_hE_5_ solution. Resonances between 3.3 and 3.5 ppm in the pure surfactant spectrum are assigned to protons of the E_5_ headgroup. The most upfield shifted resonances belong to protons of the EG group closest to the hydrophobic core, and the most downfield shifted resonances belong to protons of the EG group furthest away from the core.^9,45^

Addition of BlueH to an hC_12_hE_5_ solution causes significant peak broadening of surfactant alkyl chain signals, whereas resonances of the E_5_ headgroup remain sharp (Figure 6A). Unlike the alkyl chain protons, the E_5_ headgroup protons furthermore show only a small variation in their chemical shift. The alky chain protons are multiple protons (signal b) with sharp peaks in the absence of Blue. Peak broadening of this multiproton resonances is typically caused by the creation of differing microenvironments for these protons due to the spatial proximity of neighboring guest molecules. Hence, this peak broadening of the alkyl chain signal b points toward a localization of BlueH close to corresponding protons. Blue^–^ and Blue^2–^ induce a less severe peak broadening of signal b compared to BlueH (Figure 6B,C). They furthermore exert a smaller influence on the chemical shifts of signals a, b, and c, signaling reduced penetration of these charged Blue species into the micellar core.

Spectra recorded at higher Blue concentrations (Figure 6E,F) reveal differences between Blue^–^ and Blue^2–^: Adding Blue^–^ to a solution of hC_12_hE_5_ causes a significant peak broadening of signal b, whereas Blue^2–^ does not strongly affect the width of this resonance. This indicates that Blue^–^ has a higher affinity to the micellar core than Blue^2–^. Resonances of the E_5_ headgroup are significantly upfield shifted when either Blue^–^ or Blue^2–^ is added at a concentration of 12.5 mM. This can easily be explained by the shielding effect that the negative charge of Blue^–^ or Blue^2–^ exerts on surrounding protons. The increased separation between E_5_ proton resonances can be explained by the same argumentation as their peak broadening.^41^

**Figure 7:**
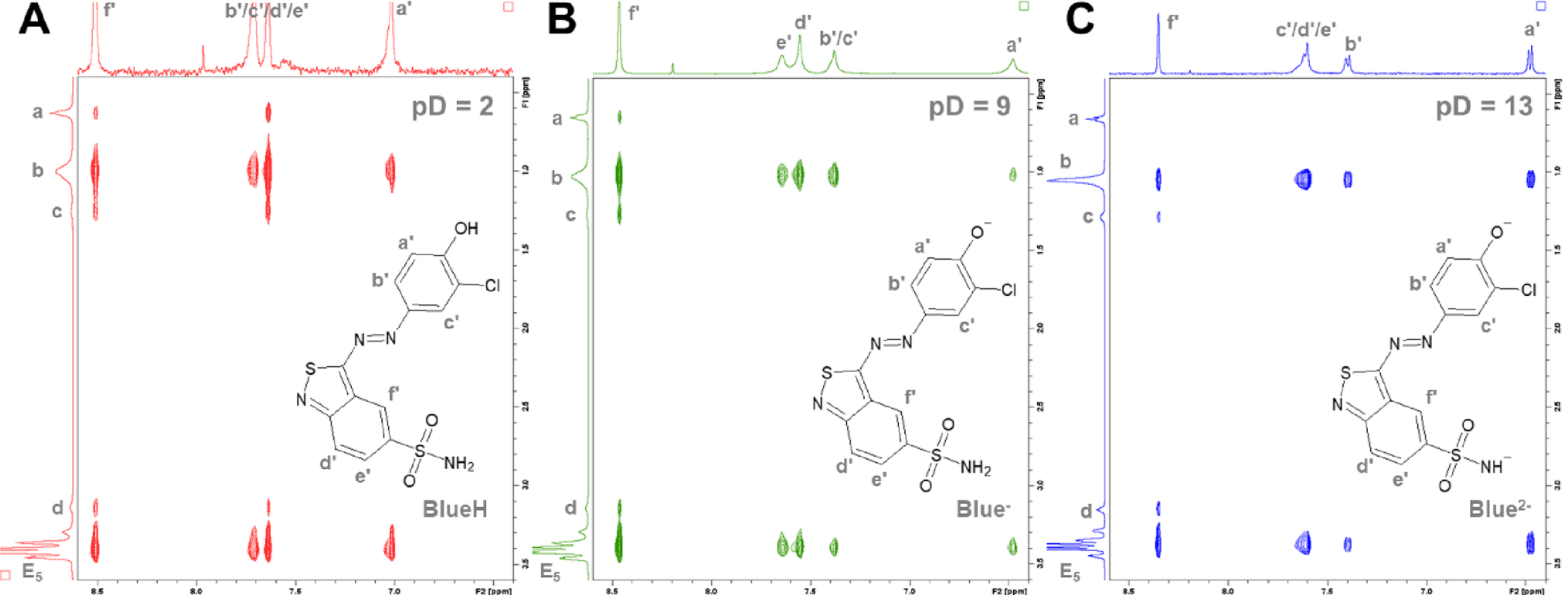
NOESY spectra recorded from solutions containing Blue at a concentration of [Blue] = 2 mM and hC_12_hE_5_ at a concentration of [hC_12_hE_5_] = 25 mM. An isotonic NaCl solution (*I* = 0.154 M) in D_2_O served as the solvent. All measurements were performed at 10 °C. Panels A, B, and C correspond to spectra of samples with pD = 2, 9, and 13, respectively. The corresponding Blue species is shown in each spectrum to indicate peak assignment. The assignment of surfactant resonances is based on Figure 6D.

Nuclear Overhauser effect spectroscopy (NOESY) further clarifies the picture of the spatial proximity between Blue and hC_12_hE_5_ protons. Based on through-space magnetic interactions (dipolar coupling), proton pairs with an interproton distance smaller than about 5 Å can be detected.^47,48^ These proton pairs can directly be identified by assigning cross-peaks in the two-dimensional NOESY spectrum. Figure 7A–C displays a relevant section of NOESY spectra showing Blue-hC_12_hE_5_ cross peaks. These spectra were recorded from solutions containing Blue at a concentration of [Blue] = 2 mM and hC_12_hE_5_ at a concentration of [hC_12_hE_5_] = 25 mM at variable pD values.

All spectra exhibit cross peaks between Blue and the alkyl chain of hC_12_hE_5_ as well as between the hydrophilic pentaethylene glycol group of Blue and the alkyl chain of hC_12_hE_5_. This suggests that the dye is in close contact with surfactant head groups as well as the hydrophobic methylene groups. Figure 7A furthermore shows discernible cross peaks between BlueH resonances and the terminal methyl group of the surfactant alkyl chain, which strengthen the hypothesis that BlueH penetrates deeply into the micellar core. The absence of cross peaks for some BlueH resonances is attributed to the broad peak width and low signal-to-noise ratio. For Blue^–^ and Blue^2–^ (Figure 7B,C), no or negligible cross peaks with the resonance of the terminal hC_12_hE_5_ methyl group occur. This remains the case for higher concentrations of Blue^–^ and Blue^2–^ (in Figure S21 of Section SI9 in the SI).

### Localization of Blue in C_12_E_5_ Micelles with SANS Contrast Matching

Contrast matching the surfactant to the solvent in solutions of Blue and C_12_E_5_ permits the detection of a *q*-dependent scattering signal, which arises from Blue only. It can therefore be used to directly investigate the localization of Blue in C_12_E_5_ micelles.

Contrast matching of C_12_E_5_ to the solvent was achieved by mixing the two differently deuterated C_12_E_5_ species mC_12_hE_5_ and mC_12_dE_5_ (Figure 1B and Experimental Methods) at a volume ratio of 18.1:81.9. The mixture is termed mC_12_mE_5_ within this work, and samples containing this mixture are labeled “C_12_E_5_-matched”.

Before discussing SANS curves from C_12_E_5_-matched samples at different pD values, a few general remarks on their analysis will be made: (1) The significance of SANS curves from samples containing a high excess of C_12_E_5_, i.e., samples that contain Blue at a concentration of either [Blue] = 1 mM or [Blue] = 2 mM, is strongly limited because of the low Blue content of each micelle under these conditions. (2) We abstained from considering any size distribution of Blue-C_12_E_5_ coassemblies for the interpretation of C_12_E_5_-matched SANS curves with models. Even though a polydispersity of certain dimensions, e.g., the length of Blue-C_12_E_5_ coassemblies, is possible, the inclusion of a distribution function would lead to overparametrization of fits. (3) The shape and size of Blue-C_12_E_5_ coassemblies are inferred from the full contrast experiments with *L*_core_ (from cylinders) shown in Table 2. (4) The morphology of the C_12_E_5_ micelles is not affected by the pD in the range of 2 ≤ pD ≤ 13 (Section SI4). (5) Isotope effects, which result from the use of surfactants with different degrees of deuteration in SANS contrast matching are expected to mostly affect correlation lengths ξ, but not the assembly cross section if compared to full contrast measurements (Section SI6).

**Figure 8:**
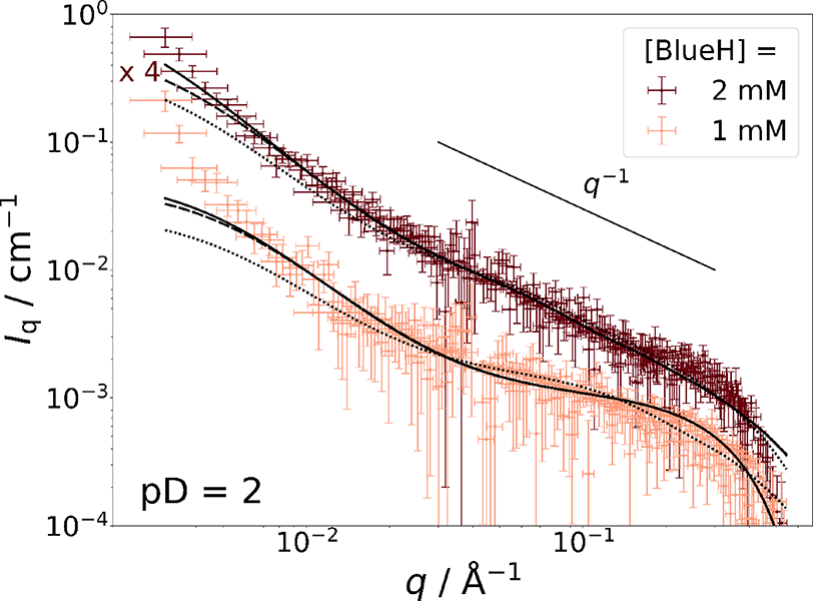
C_12_E_5_-matched SANS curves from solutions with pD = 2 containing [mC_12_mE_5_] = 25 mM at different concentrations of Blue^–^ in an isotonic NaCl solution prepared in D_2_O at 10 °C. Dotted line (····): fit with the form factor of a cylinder including Ornstein–Zernike scattering as a structure factor with κ and ξ obtained from the corresponding SANS full contrast measurement. Dashed line (**---**): fit with the same model but only ξ was kept constant according to the value obtained from the SANS full contrast measurement. Solid line (**—**): fit with the same model but ξ was kept constant according to a value obtained from static light scattering (Section SI7). Parameters for each fit can be found in Table S7. In addition to that, a direct comparison to full contrast data is given in Figure S22.

The presentation of the results starts with the SANS experiments on the incorporation of BlueH in its lowest degree of hydrophilicity. Figure 8 displays SANS curves from solutions containing mC_12_mE_5_ at a concentration of 25 mM and BlueH at a concentration of either 1 or 2 mM (pD = 2). Whereas for [BlueH] = 1 mM a power law according to *I*_q_ ∼ *q*^–1^ is hardly discernible, it is clearly observed at *q* > 0.01 Å^–1^ for [BlueH] = 2 mM. This observation points toward a rod-like arrangement of BlueH, in line with the clear-cut evidence for rod-like structures provided by the respective full-contrast experiments. Therefore, experimental SANS curves were fitted with a form factor for rigid cylinders^25,49^ while including Ornstein–Zernike scattering (*C*(*q*), eq 2) in place of *S*(*q*) according to
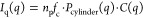
3

In eq 3, *n*_p_ is the particle number density and *f*_c_ is a factor correcting for uncertainties in the contrast. The product *n*_p_*f*_c_ was fitted and termed “apparent particle number density” in all analyses concerning C_12_E_5_-matched SANS data. The contrast of cylindrical particles is related to the scattering length density difference between the solvent and cylinder, which was fixed to Δη = 3.358 · 10^–6^ Å^–2^. This value corresponds to the scattering length density difference between BlueH and the solvent (Table S2). The inclusion of Ornstein–Zernike scattering into the evaluation of SANS curves from solutions containing BlueH and C_12_E_5_ at pD = 2 is necessary because the addition of BlueH promotes attractive interactions between micelles.

The Ornstein–Zernike term was established by transferring correlation lengths ξ from fits to full contrast SANS data or by using ξ obtained from independent static light scattering (Section SI7) experiments. Better fits were obtained when the scaling parameter κ, which relates to the isothermal osmotic compressibility, was varied (Figure 8 and Table S7) instead of also being transferred from full contrast SANS experiments.

In both concentrations, cylindrical BlueH aggregates with a cross-section radius (*r*_cylinder_) of less than 6 Å were observed. Considering that the core radii derived from the corresponding full contrast SANS data lie in the regime of *r*_core_ ∼ 13 Å, in line with the length of a fully extended C_12_ alkyl chain of 16.7 Å^50^, it gets obvious that the resulting cylindrical domains formed by BlueH in C_12_E_5_ solution are easily accommodated by the hydrophobic core region of cylindrical C_12_E_5_ micelles. A schematic outline of this result together with an overview of the results from model fitting is displayed in Table 3. Contrast matched SANS is fully compatible with findings from the analysis of NMR spectra (Figures 6 and 7), which indicate the interaction between BlueH and the hydrophobic C_12_ alkyl chain of C_12_E_5_.

**Table 3:**
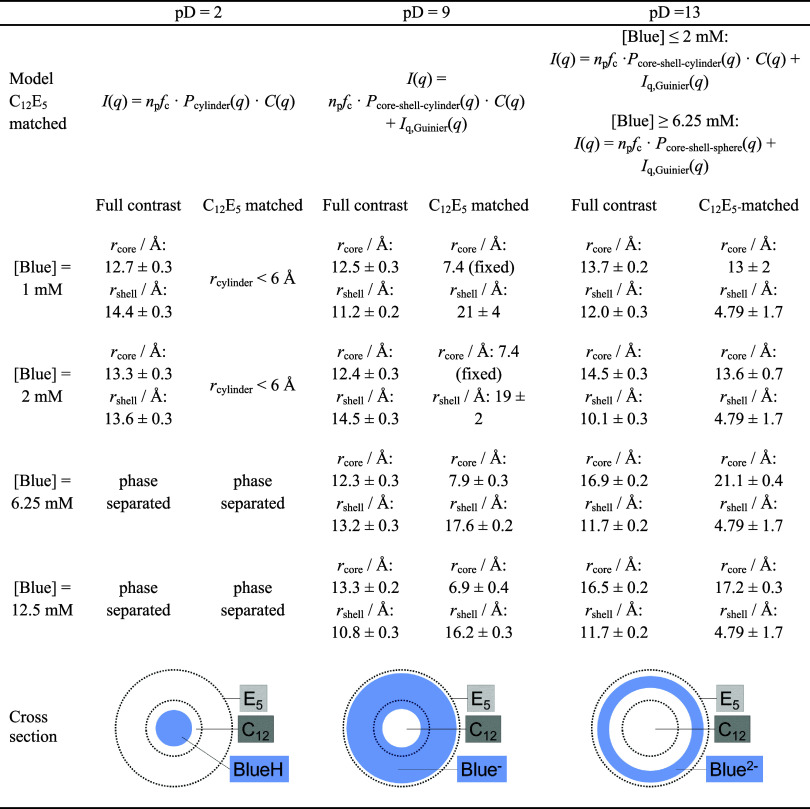
Form Factor Models for Fitting SANS Curves from C_12_E_5_-Matched Solutions with [mC_12_mE_5_] = 25 mM Containing the Indicated Concentration of Blue at the Given pD^a^

a The apparent particle number density (*n*_p_*f*_c_) and cross-section size parameters were fitted. Cross-section size parameters that resulted from such fits to C_12_E_5_ matched SANS curves are compared to cross-section size parameters that were obtained from fits to the high-*q* region of corresponding full contrast SANS curves (step 1 of the two-step fitting strategy for full contrast SANS curves). Full contrast cross-section dimensions describe a cylinder cross section in all cases except for samples with pD = 13 and [Blue^2–^] ≥ 6.25 mM, where *r*_core_ corresponds to the core radius and *r*_shell_ to the shell thickness of a core-shell sphere. In the full contrast case, the value of *r*_core_ corresponds to the mean core radius. In the C_12_E_5_ matched case, no distribution of *r*_core_ was assumed. The value of *r*_cylinder_ corresponds to the radius of a homogeneous cylinder, which depends on the Ornstein–Zernike parameters that were set during fitting. All parameters can be found in the tables of Section SI10 in the SI.

**Figure 9:**
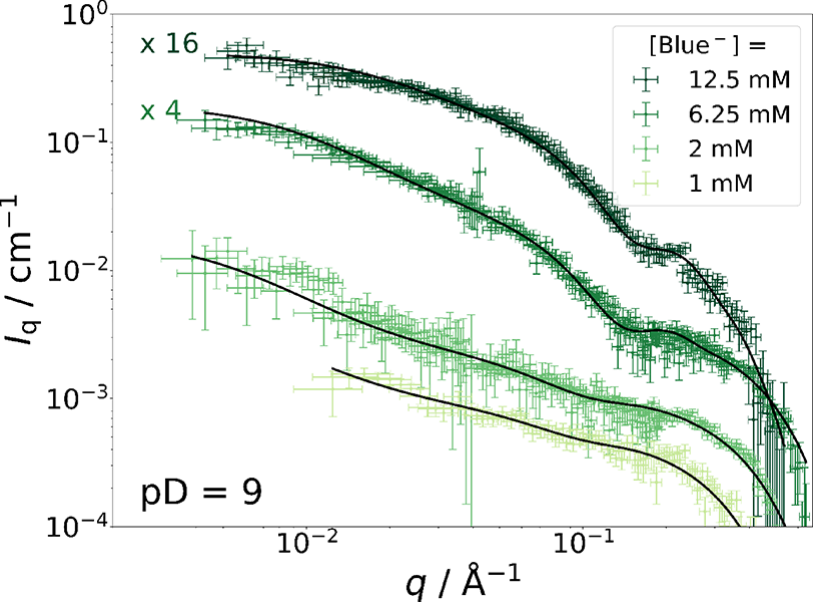
C_12_E_5_-matched SANS curves from solutions with pD = 9 containing [mC_12_mE_5_] = 25 mM at variable concentrations of Blue^–^ in an isotonic NaCl solution prepared in D_2_O at 10 °C. Solid line (**- - - -**): fit with the model described by eq 4. Parameter values for each fit can be found in Table S8. A direct comparison to full contrast data is given in Figure S23.

Increasing the pD from 2 to 9 increases the hydrophilicity of Blue as we are now dealing with negatively charged Blue^–^. Figure 9 displays C_12_E_5_ contrast-matched SANS curves from solutions containing mC_12_mC_5_ at a concentration of 25 mM and variable concentrations of Blue^–^ at pD = 9.

The SANS curves shown in Figure 9 all close with a strong decay of the scattering intensity *I*_q_(*q*) at the high-*q* end, which is observed in addition to the micellar form factor. Because the ionic character of Blue makes dissolution of Blue molecules as single ions or very small oligomers likely, the decay at the high-*q* end can be interpreted as a Guinier shoulder of dissolved Blue entities. The SANS curves were therefore fitted with the following model, which assumes the coexistence of micellar domains of Blue^–^ (*P*_core–shell-cylinder_) and of molecularly dissolved Blue^–^ or small oligomers thereof:
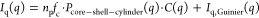
4
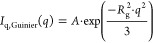
5

*I*_q,Guinier_(*q*) is a Guinier term, which describes scattering from molecularly dissolved Blue^–^ molecules (or small oligomers thereof) and accounts for the decay of the scattering intensity at *q* > 0.2 Å^–1^. In eq 5, *R*_g_ is the radius of gyration, and *A* is the scaling factor or forward scattering intensity of the monomers/oligomers. By performing Guinier fits according to eq 5 to the *q*-range of *q* > 0.2 Å^–1^, the value of *R*_g_ was identified to lay between 4.2 and 6.5 Å for all samples. This is compatible with a recent estimation of *R*_g_ of Blue dimers to *R*_g_ ≈ 4 Å.^51^ It needs to be pointed out that these values have a fairly large uncertainty due to the low scattering intensity (*I*_q_ < 0.001 cm^–1^) and the high signal-to-noise ratio in this *q*-range. Nevertheless, parameters from Guinier fits were kept constant in subsequent fits using eq 4.

All SANS curves with contrast matched C_12_E_5_ at pD = 9 can be satisfactorily described by the form factor *P*_core–shell-cylinder_ of core–shell cylinders inserted in eq 4, assuming that Blue^–^ establishes the shell of a “hollow” core. The length of these cylinders was fixed to 66 Å for [Blue^–^] ≤ 2 mM and to 40 Å for [Blue^–^] ≥ 6.25 mM, which correspond to the core length of Blue^–^-C_12_E_5_ assemblies inferred from corresponding full contrast measurements (Table 2). The Ornstein–Zernike parameters κ and ξ were also fixed to values obtained from the corresponding full contrast measurements. The contrast between the solvent and the core was set to Δη_core_ = 0, and the contrast between the solvent and the shell was set to a value of Δη_shell_ = 3.358 · 10^–6^ Å^–2^ (Table S2). The possible inaccuracy of this contrast is compensated by fitting the apparent particle number density (*n*_p_*f*_c_). The described strategy left *n*_p_*f*_c_ and the cross-section dimensions (*r*_core_ and *r*_shell_) of the core–shell cylinder to be identified. A simultaneous variation of these parameters during fitting of eq 4 to experimental SANS curves led to the cross-section dimensions displayed in Table 3 for samples containing [Blue^–^] ≥ 6.25 mM. For the analysis of SANS curves from samples containing [Blue^–^] ≤ 2 mM, *r*_core_ was fixed to 7.4 Å, which corresponds to the average of the two *r*_core_ values obtained from SANS curves of samples with [Blue^–^] = 6.25 and 12.5 mM, leaving only *n*_p_*f*_c_ and *r*_shell_ to be fitted. This was done because of the high signal-to-noise ratio of SANS curves from samples with [Blue^–^] ≤ 2 mM. The complete parameter set is summarized in Table S8.

The core radius *r*_core_ obtained for the core–shell cylinders formed by Blue^–^ is smaller than the core radius found for Blue^–^-C_12_E_5_ comicelles by the analysis of full contrast SANS curves and smaller than the length of a fully extended C_12_ alkyl chain (<16.7 Å).^50^ Furthermore, the sum *r*_core_ + *r*_shell_ obtained from analysis of full contrast and C_12_E_5_-matched SANS curves are similar, suggesting that Blue^–^ is mainly located in the EG region. As a consequence, the Blue^–^ ions establish a hollow cylinder. Blue^–^ obviously penetrates less deep into the hydrophobic core than BlueH does at pD = 2. This is schematically depicted in Table 3. Results from SANS are fully in line with the findings from NMR spectroscopic analysis, which suggests the interaction between Blue^–^ and both the alkyl part and the EG part of the C_12_E_5_ surfactant.

**Figure 10:**
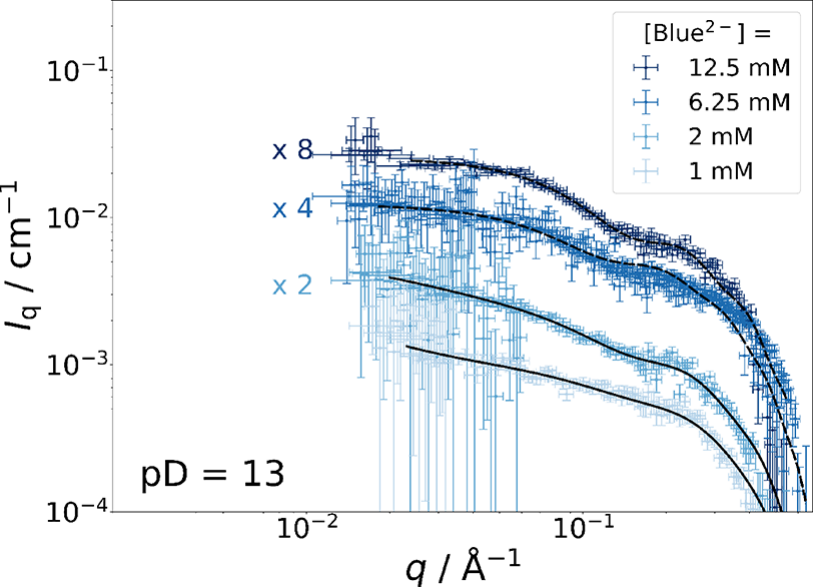
C_12_E_5_-matched SANS curves from solutions with pD = 13 containing [mC_12_mE_5_] = 25 mM at variable concentrations of Blue^–^ in an isotonic NaCl solution prepared in D_2_O at 10 °C. Dashed lines (**----**) correspond to form factor fits according to eq 6. Solid lines (**- - -**) correspond to form factor fits according to eq 4. Precise parameters for each fit can be found in Table S9. In addition to that, a direct comparison to full contrast data is given in Figure S24.

At pD = 13, Blue^2–^ is the only species of Blue existing. Based on information from NMR-spectroscopy, Blue^2–^ is not expected to significantly penetrate into the micellar core but to interact with the EG headgroup. This implies that scattering from a core–shell arrangement is observed in the C_12_E_5_-matched case, with Blue^2–^ spreading throughout the shell and leaving a core with zero contrast (Δη = 0) to the solvent. In addition to the micelles, the presence of molecularly dissolved Blue^2–^ may occur in a similar way as has already been discussed at pD = 9. Such small entities cause the Guinier shoulder of the scattering intensity that is observed at *q* > 0.2 Å^–1^. Analogous to the evaluation of C_12_E_5_-matched SANS curves at pD = 9, the form factor *P*(*q*) that describes the Blue molecules incorporated into C_12_E_5_ micelles was based on information from full contrast data (Table 2). At a concentration of [Blue^2–^] = 1 or 2 mM, core–shell cylinders with a core length of *L*_core_ = 40 Å are formed when Blue^2–^ is added to a C_12_E_5_ solution. Therefore, eq 4 was applied to corresponding C_12_E_5_-matched curves, which are displayed in Figure 10. The Ornstein–Zernike parameters were likewise fixed to values obtained from fits to corresponding full contrast SANS curves.

At Blue^2–^ concentrations of [Blue^2–^] = 6.25 or 12.5 mM, spherical Blue^2–^-C_12_E_5_ coassemblies were observed with the full-contrast experiments (Table 2), and eq 6 was used instead of eq 4:
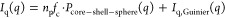
6

In eq 6, *P*_core–shell-sphere_(*q*) is the form factor of a core–shell sphere and *n*_p_*f*_c_ is the apparent number density of core–shell particles.

Figure 10 displays all C_12_E_5_ contrast-matched SANS curves from samples containing mC_12_mE_5_ at a concentration of 25 mM and variable concentrations of Blue^2–^ measured at pD = 13. Preliminary Guinier analysis of the high-*q* region of all four SANS curves shown in Figure 10 according to eq 5 suggests an average *R*_g_(Blue^2–^) = 5.4 Å, which was kept constant during analysis with eq 4 or 6. Because of the low absolute scattering intensity and high signal-to-noise ratio of experimental SANS curves, *R*_g_ is affected by a large uncertainty. For fitting eq 4 or 6 to the SANS curves shown in Figure 10, values of the scattering contrast were fixed (Δη_core_ = 0 and Δη_shell_ = 3.358 · 10^–6^ Å^–2^), but the apparent number density of core–shell assemblies (*n*_p_*f*_c_) varied by fitting to compensate for the inaccuracies of the chosen scattering contrast. Successful fitting does not only confirm the morphologies inferred from the full-contrast experiments but, more importantly, revealed the spatial distribution of Blue^2–^ within these micelles.

For all four SANS curves shown in Figure 10, *r*_shell_ was fitted globally to *r*_shell_ = 4.79 Å and *r*_core_ was fitted individually. Values of *r*_core_ are displayed in Table 3. In cases where eq 4 was applied, *r*_core_ obtained from fits to C_12_E_5_-matched SANS curves are similar to *r*_core_ obtained from fits to full contrast SANS curves. In cases where eq 6 was applied, *r*_core_ is larger than the values of *r*_core_ obtained by the analysis of corresponding full contrast data and larger than the length of a completely extended C_12_ alkyl chain (>16.7 Å).^50^ These observations point toward the preferential localization of Blue^2–^ molecules in the headgroup region of C_12_E_5_ micelles for all four cases. Based on the necessity to include a Guinier contribution to account for molecularly dissolved Blue^2–^ molecules, Blue^2–^ is considered to be distributed between the headgroup region of C_12_E_5_ micelles and the bulk phase of the solution.

## Conclusions

Given a temperature of 10 °C and a surfactant concentration of 25 mM, the nonionic surfactant C_12_E_5_ forms elongated cylindrical micelles independent of the pH within a regime of 2 ≤ pH ≤ 13. In this very pH regime, the dyestuff Blue changes its degree of hydrophilicity from the neutral state of an undissociated BlueH at pH = 2 all the way to a negatively charged bivalent Blue^2–^ ion at pH = 13, with hardly any changes in the morphology of the Blue molecules throughout this large variation of the hydrophilicity. As the C_12_E_5_ micelles that remain unaffected by pD in its pure state host Blue molecules in all different solution states, this coincidence offers the unique chance to study the sole impact of a varying degree of hydrophilicity of guest molecules on the morphology of the hosting micelles.

It is exactly this study that was pursued in the present work with a joint application of SANS and NMR. Whereas full contrast SANS experiments revealed the morphology of the micelles throughout the entire space of solution conditions, contrast matching of the hosting surfactant molecules to that of the solvent provided a significant insight into the spatial distribution of Blue in the micelles. The results thereof could be supported and supplemented by ^1^H NMR spectroscopy and NOESY, as they teach us the nature of the immediate surrounding of the Blue molecules/ions and/or the surfactant.

A view on the modifications of the solution behavior at variable Blue concentrations measured at four different pH values of 2, 9, 12, and 13 gives a first clue on the solution behavior of the mixed Blue-C_12_E_5_ micelles. At pH = 2, addition of Blue as BlueH shifts the clouding temperature (CT) to a lower value, bringing it closer to the temperature (10 °C) under consideration in the present work. At pH > 9, negatively charged Blue ions exist with an increasing amount of Blue^2–^ as the pH is further increased. This causes a reverse shift of CT; i.e., now the CT moves to a higher temperature than that of the pure surfactant state, with the shift getting larger as the pH increases from pH = 9 to 13. Independent of the direction of this shift from the reference state established by the pure surfactant, that shift increases with increasing concentration of Blue.

Within this phase space summarized in Figure 2, full contrast SANS reveals spherical micelles at pH = 13, with a tendency to elongate to cylindrical micelles as the pH is lowered. In Figure 2, such trends proceed along vertical lines representing fixed Blue concentrations. Because the lowering of the pH parallels with the molecular hydrophilicity of Blue, this gradual decrease in hydrophilicity first elongates the mixed spherical micelles to cylinders and at pH = 2 brings the system even beyond the reference line of pure C_12_E_5_, micelles. This feature is more pronounced the larger the Blue concentration gets as, for obvious reasons, elongated cylinders of a length of some 10 Å already exist in the absence of Blue no matter what pH the solution has. Decreasing the hydrophilicity of a charged guest molecule to the rather hydrophobic state of an undissociated neutral molecule brings the solution of micellar coassemblies ever closer to the CT. Such an effect is comparable to gradually increasing the temperature of pure C_12_E_5_ micelles.

Finally, matching the scattering contrast of the hosting surfactant molecules to that of the solvent background enabled us to localize the dyestuff Blue within the mixed micelles. At pH = 13, Blue occurs as a bivalent anion and is predominantly located in the polar headgroup zone of the E_5_. With decreasing pH, the dyestuff changes its hydrophilicity and moves toward the hydrophobic core, which establishes the main location of BlueH at pH = 2. These findings are in full agreement with supplementary NMR experiments.

The sphere-to-cylinder transformation not only is caused by the disappearance of negative charges from Blue^2–^ but also can be reconciled with an optimal packing^52,53^ caused by the movement of the Blue from the polar periphery to the hydrophobic core as it is discharged. At high pH, location of Blue in the headgroup zone increases the cone-like nature of the building units. The more cone-like a building unit is, the stronger is the tendency to fit into spheres. The more Blue moves toward the center, the larger the deviation of a cone-like structure gets, thereby shifting an optimal fitting to cylinder-like morphologies.

To conclude, the present case study successfully demonstrates that a joint application of SANS including contrast matching of one component together with ^1^H NMR including NOESY is capable of unraveling the morphology of mixed micelles of a binary system and of revealing the spatial distribution of the two components within the micelle.

## Supporting Information Available

Determination of Blue acid dissociation constants; form factor models used to interpret SANS curves; details on fits to full contrast SANS curves; impact dependence of solution pD on C_12_E_5_ self-assembly; evaluation of light scattering data; isotope effects; light scattering from BlueH/C_12_E_5_ solutions at pD = 2; determination of the SANS match point of C_12_E_5_ in D_2_O; NMR spectra; comparison of parameters from full contrast and C_12_E_5_ contrast matched SANS; and synthesis of deuterated C_12_E_5_. The authors have cited additional references within the Supporting Information.^54−67^ (PDF)
