# Supplementary material for: Impact of Additive Hydrophilicity on Mixed Dye-Nonionic Surfactant Micelles: Micelle Morphology and Dye Localization
Source: Langmuir. 2024 Apr 19;40(17):8872–85. doi: 10.1021/acs.langmuir.4c00012 (PMC11536388; doi:10.1021/acs.langmuir.4c00012)
Supplement: Supplementary file 1 — la4c00012_si_001.pdf [file la4c00012_si_001.pdf]

Supporting Information

Impact of Additive Hydrophilicity on Mixed Dye-Nonionic Surfactant Micelles: Micelle Morphology and Dye Localization

Wenke Müller[a], Weronika Sroka[a], Ralf Schweins[a], Bernd Nöcker[b], Jia-Fei Poon[c,d], Klaus Huber*[e]

[a] Dr. W. Müller, W. Sroka, Dr. R. Schweins
Science Division / Large Scale Structures Group
Institut Laue-Langevin
71 Avenue des Martyrs, 38000 Grenoble, France

[b] Dr. B. Nöcker
Basic Research & Technology Development
KAO Germany GmbH
Pfungstädter Straße 98-100, 64297 Darmstadt, Germany

[c] Dr. J.-F. Poon
European Spallation Source
Box 176, SE-221 00 Lund, Sweden

[d] Dr. J.-F. Poon
Food Technology, Engineering and Nutrition
Lund University
Box 117, SE-221 00 Lund, Sweden

[e] Prof. Dr. K. Huber
Fakultät für Naturwissenschaften / Physical Chemistry
Universität Paderborn
Warburger Straße 100, 33098 Paderborn, Germany
E-mail: klaus.huber@upb.de

Table of Content

SI1. Determination of Blue acid dissociation constants 2

SI2. Form Factor Models used to interpret SANS curves 4

SI3. Details on fits to full contrast SANS curves 6

SI4. Dependence of $C_{12}E_5$ self-assembly on solution pD 15

SI5. Light scattering – Data Evaluation 16

SI6. Isotope effects 19

SI7. Light scattering from BlueH/$C_{12}E_5$ solutions at pD = 2 26

SI8. Determination of the SANS match point of $C_{12}E_5$ in D_2O 30

SI9. NMR-spectra 32

SI10. Comparison of parameters from full contrast and $C_{12}E_5$ contrast matched SANS 34

SI11. Synthesis of deuterated $C_{12}E_5$ 41

SI12. References 48

SI1. Determination of Blue acid dissociation constants

The pK_a values of Blue could be determined by spectroscopic pD-titration because the absorption spectrum of Blue is sensitive to pD-changes (Figure S1). UV/vis spectra were recorded from solutions containing Blue at a concentration of [Blue] = 0.1 M and $hC_{12}hE_5$ at a concentration of $[hC_{12}hE_5]$ = 12.5 mM at room temperature (22 °C) using a Jasco V-630 UV-vis spectrophotometer. Samples were prepared and handled as described in the **Section Chemicals and Sample Preparation** in the main document. Added volumes of DCl and NaOD solution were noted to be able to precisely determine the concentration of Blue ([Blue]) in each sample. This is needed for the calculation of the molar extinction coefficient ε according to the Beer-Lambert law:[1]

$$\varepsilon = \frac{A}{d \cdot [\text{Blue}]} \qquad \text{(SI1)}$$

In **eq (SI1)**, A is the absorbance of the sample and d the optical path length, which was d = 0.1 cm (Hellma QS cuvette) for the present experiment.

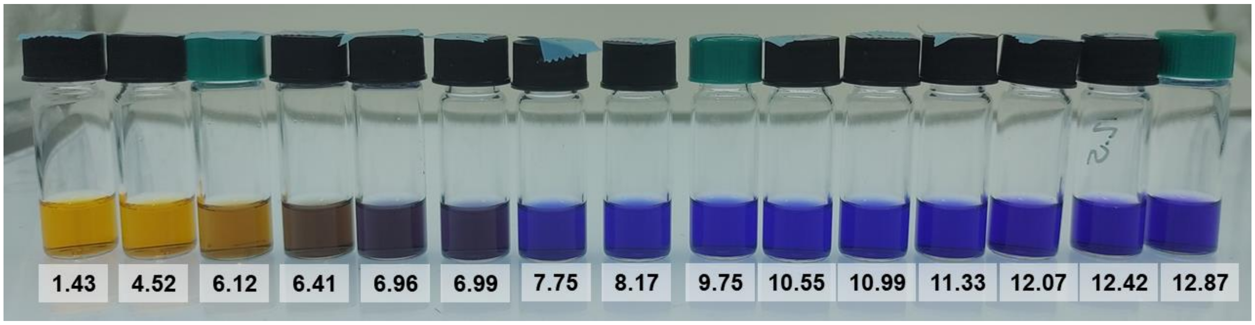

Figure S1: Photo of samples containing Blue at a concentration of [Blue] = 0.1 mM and $hC_{12}hE_5$ at a concentration of $[hC_{12}hE_5]$ = 12.5 mM at variable solution pD. The pD is indicated below each sample. An isotonic NaCl solution (I = 0.154 M) in D_2O served as the solvent. The pD of each solution was adjusted using 1 M solutions of DCl or NaOD. Solutions were kept at room temperature (22 °C).

Figure S2A shows the evolution of ε at a wavelength of 583 nm as a function of solution pD. This wavelength corresponds to the wavelength of maximum absorbance (λ_{max}) of solutions containing Blue in its one-fold deprotonated form ($Blue^-$). A decrease in ε(583 nm) therefore signals the protonation of Blue to form BlueH. From **Figure S2A**, the first pK_a (pK_{a1}) describing the acid/base equilibrium between BlueH and $Blue^-$ was obtained. This was done by determining the pD at which [BlueH] = $[Blue^-]$, accomplished by interpolating to the pD at which an average ε(583 nm) is obtained with respect to ε(583 nm) for BlueH and ε(583 nm) for $Blue^-$. Following this analysis, pK_{a1} was determined to pK_{a1} = 6.8.

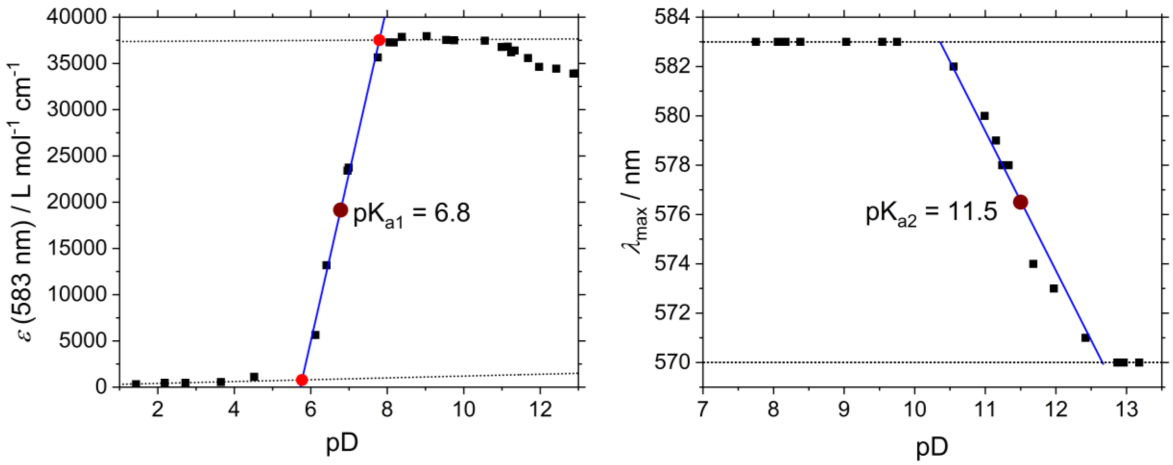

Figure S2: UV/vis spectroscopic pD-titration of samples containing [Blue] = 0.1 mM and [$hC_{12}hE_5$] = 12.5 mM at room temperature. An isotonic NaCl solution in D_2O was used as a solvent. The pD of each solution was adjusted using DCl and NaOD. **A** For the determination of pK_{a1}, the molar extinction coefficient at a wavelength of 583 nm was evaluated as a function of pD. **B** For the determination of pK_{a2}, the position of the absorption maximum (λ_{max}) was evaluated as a function of pD.

Figure S2B shows the wavelength of maximum absorbance λ_{max} as a function of solution pD. The absorption maximum of $Blue^-$ is red-shifted compared to the absorption maximum of $Blue^{2-}$. With this red-shift, pK_{a2}, which describes the acid/base equilibrium between $Blue^-$ and $Blue^{2-}$ was identified as the pD, at which the UV/vis spectrum showed an average λ_{max} at the average value corresponding to (583nm+570nm)/2. This analysis yields pK_{a2} = 11.5.

SI2. Form Factor Models used to interpret SANS curves

Table S1: Denotation of form- and structure factors used in this work compared to their name in the SASfit user guide.[5]

Denotation in the current work	Denotation in the SASfit user guide
**Form factors**	
**Form factors for fitting the entire *q*-range of full contrast SANS curves**	
Form factor of a core-shell sphere: $P_{\text{core-shell-sphere}}(q)$	Spherical Shell iii
Form factor of an end-capped core-shell cylinder with core length L_{core}: $P_{\text{cap-core-shell-cylinder}}(q)$	CylShell2
**Form factors for fitting the entire *q*-range of $C_{12}E_5$-matched SANS curves**	
Form factor of a cylinder: $P_{\text{cylinder}}(q)$	Cylinder
Form factor of a sphere: $P_{\text{sphere}}(q)$	Sphere
Form factor of a core-shell cylinder with length L (not end-capped) $P_{\text{core-shell-cylinder}}(q)$	CylShell1
Extended Guinier plateau: $I_{\text{q,Guinier}}(q)$	generalized Guinier law (α = 0)
**Form factors for fitting the high-*q*-range of full contrast SANS curves (*q* > 0.045 Å^{-1})**	
Core-shell cylinder with linearly decaying shell contrast $P_{\text{core-linshell-cylinder}}(q)$	Pcs:linear shell cyl. · P'(Q): Rod
Core-shell sphere with linearly decaying shell contrast $P_{\text{core-linshell-sphere}}(q)$	LinShell
**Structure factors**	
Ornstein-Zernike expression $C(q)$	Critical Scattering
Hard sphere interactions $S(q)$	3D Hard Sphere (PY)

SANS curves were fitted with form factors and structure factors available in the SASfit software package (Version: 0.94.12, documentation from 09.01.2023).[4,5] In the case of spherically symmetric objects, the q-dependency of the scattering intensity is described as:[6]

$$I_q(q) = n_p \cdot P(q) \cdot S(q) \qquad \text{(SI2)}$$

In **eq (SI2)**, n_p is the number density of scattering particles and $P(q)$ the single particle form factor, which describes the shape and contrast of a single particle. In the presented case, it returns the scattering cross section of that particle [cm^2]. $S(q)$ is the structure factor, which describes interparticle correlations. In case of concentration fluctuations due to attractive interparticle interactions it is replaced by an Ornstein-Zernike expression $C(q)$.

$$C(q) = 1 + \frac{\kappa}{1 + q^2\xi^2} \qquad \text{(SI3)}$$

A complete description of all form- and structure factors is provided in the SASfit user guide ("User guide for the SASfit software package", version 0.94.12 from the 9th of January 2023).[5] **Table S1** provides an overview on all SASfit models used in the present work.

Model form factors in **Table S1** of core-shell structures are based on scattering length density profiles. Data to establish such profiles are summarized in **Table S2**.

Table S2: Scattering length densities used for the calculation of scattering length density profiles for form factor fitting.

Designation	Sum formula	Scattering length density η / 10^{-6} Å^{-2}
solvent	D_2O	6.358
hC_{12} (hydrogenated C_{12} alkyl chain)	$C_{12}H_{25}$	-0.392
hE_5 (hydrogenated E_5 pentaethyleneglycol group)	$C_{10}H_{21}O_6$	0.731
dE_5 (deuterated E_5 pentaethyleneglycol group)	$C_{10}D_{21}O_6$	7.750
BlueH	$C_{13}H_9ClN_4O_3S_2$	3.0

SI3. Details on fits to full contrast SANS curves

Solutions for SANS with full contrast were prepared in an isotonic NaCl solution in D_2O and measured at a sample temperature of 10 °C. Preliminary fits revealed either core-shell spheres or end-capped core-shell cylinders with an L_{core} of 66 Å. For the analysis of full contrast SANS curves emerging from Blue/$C_{12}E_5$ solutions, the following strategy was employed:

In a first step, only the high-*q* range ($q > 0.045$ Å^{-1}) was fitted with a core-shell model to obtain information on the ensemble average assembly cross section. No structure factor or Ornstein-Zernike contribution was assumed in this step.

In a second step, the entire *q*-range was fitted with various form factor models, including a structure factor S(q) or Ornstein-Zernike scattering *C*(q). For this analysis, the core radius and its distribution were adapted from step one and kept constant during fitting. All other parameters, including shell thickness and scattering length densities, were fitted for a set of different form factors, including core-shell spheres and end-capped core-shell cylinders. **Table S1** presents an overview on all scattering models applied in the present work.

Assessment of the number density of assemblies (n_p). Assemblies were assumed to possess a core-shell morphology in all cases, with a core volume accessible from L_{core} and r_{core} in case of cylinders and from r_{core} only in case of spheres. This core volume led to an estimation of the aggregation number by dividing this core volume by the molecular volume of a C_{12} alkyl chain of 350.2 Å^3.[8] Finally, the particle number density n_p was calculated resulting simply by dividing the total surfactant concentration by the aggregation number, assuming that all $C_{12}E_5$ molecules participate in micelle formation. Scattering from molecularly dissolved $C_{12}E_5$ molecules was not considered due to the low critical micelle concentration of $C_{12}E_5$ (< 0.1 mM in many aqueous solvents) and a subsequent excess of micellized surfactant at a $C_{12}E_5$ concentration of 25 mM.[28] For the fits of the entire *q*-range, the calculated n_p was kept constant, whereas scattering length densities were fitted.

Analysis of assembly cross section. In a first step, information on the assembly cross section were obtained from form factor fits to the high-*q* range ($q > 0.045$ Å^{-1}). No structure factor was considered. Model form-factors of core-shell cylinders (without end-caps) and core-shell spheres were applied to fit experimental data. In case of cylinders, insensitivity of the high-*q* regime to the exact length of cylinders led us to fix the total cylinder length to $L = 90$ Å. This length corresponds to a core length of $L_{core} = 66$ Å, typically found during the preliminary fits mentioned above. Resulting fits are presented in **Figure S3** and cross section dimensions obtained from the fits are

summarized in **Figure S4**. **Table S3** compares χ^2_{red} values obtained for fits to experimental SANS data in the high-q range (q > 0.045 Å^{-1}) with the two discussed form factor models. It furthermore displays the standard deviation of the core radius $SD(r_{core})$ resulting from the number-weighted log-normal distribution of the core radius r_{core} as a measure for the polydispersity of r_{core}.

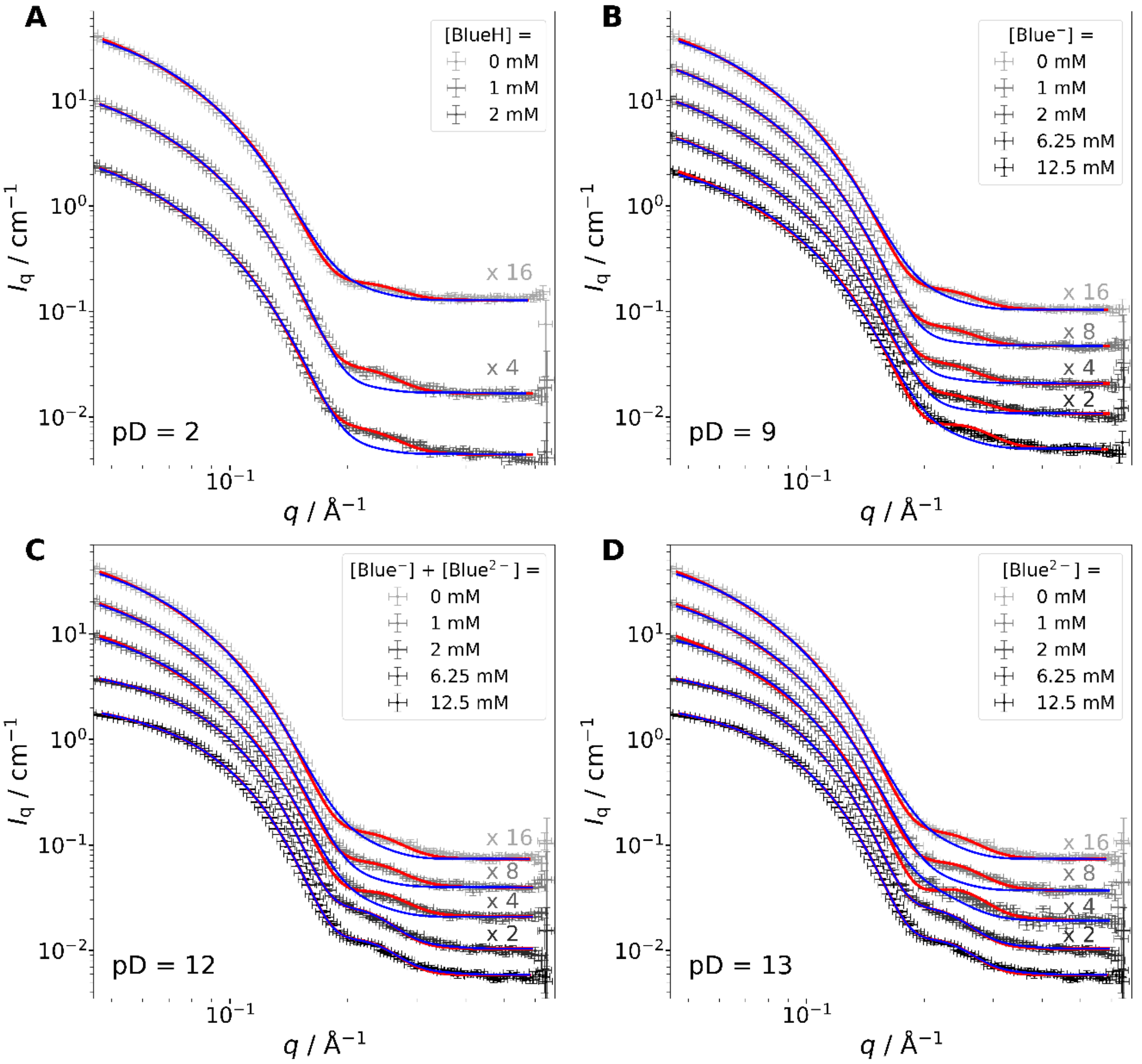

Figure S3: High-q region of full contrast SANS curves recorded from samples containing $[hC_{12}hE_5]$ = 25 mM, and Blue at the indicated concentration at **A** pD = 2, **B** pD = 9, **C** pD = 12 or **D** pD = 13. An isotonic NaCl solution (I = 0.154 M) in D_2O served as the solvent. SANS curves were recorded at a sample temperature of 10 °C. Blue lines (—): Form factor fit assuming core-shell spheres and a linearly decaying scattering length density difference profile ($\Delta\eta$) within the shell region of the sphere ($P_{core\text{-}linshell\text{-}sphere}(q)$). Red lines (—): Form factor fit assuming core-shell cylinders with a total length of L = 90 Å and a linearly decaying scattering length density difference profile ($\Delta\eta$) within the shell region of the cylinder cross section ($P_{core\text{-}linshell\text{-}cylinder}(q)$).

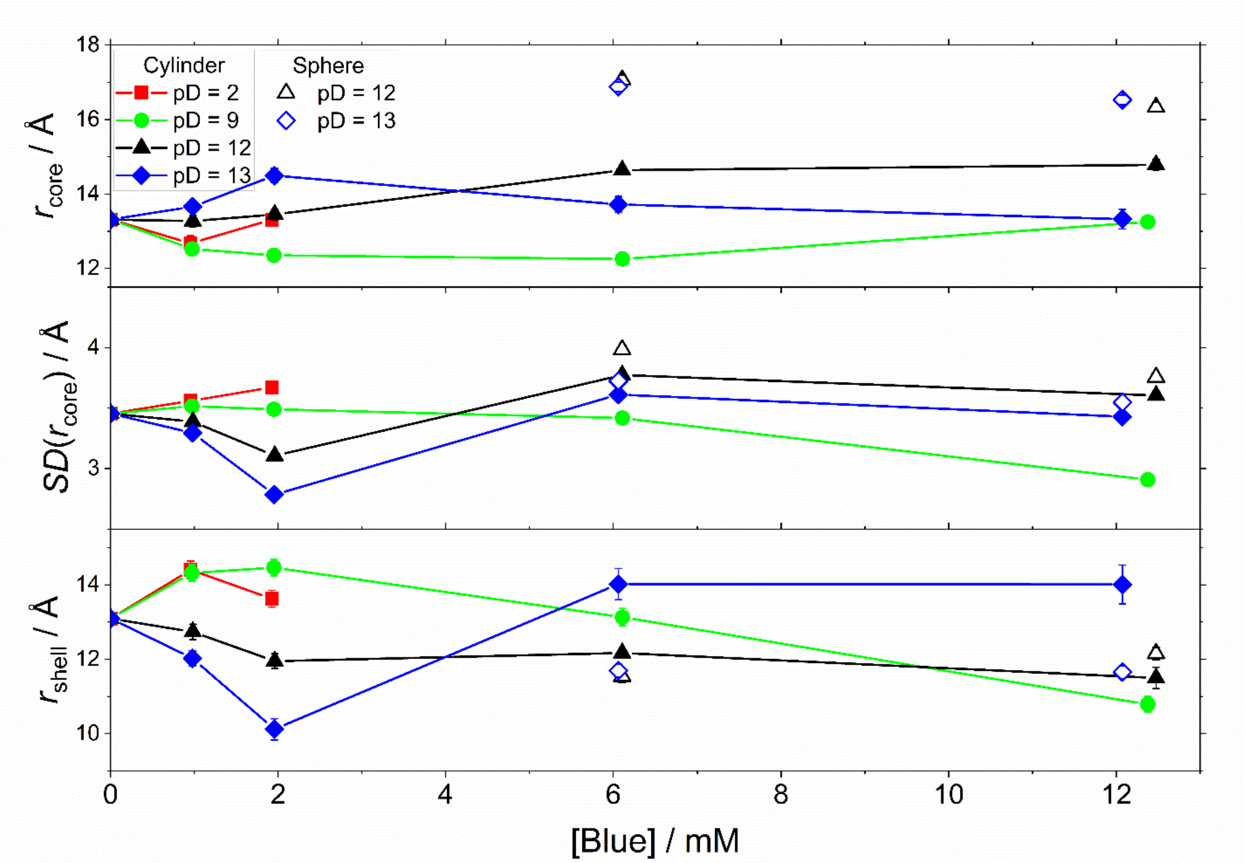

Figure S4: Size parameters for the cross section of Blue/$C_{12}E_5$ micelles obtained from fitting the high-*q* range (*q* > 0.045 Å^{-1}) of full contrast SANS curves emerging from solutions containing $hC_{12}hE_5$ at a concentration of 25 mM. An isotonic solution of NaCl in D_2O was used as a solvent and measurements were performed at a sample temperature of 10 °C. The form factor model of a core-shell cylinder with a length of 90 Å and linearly decaying $\Delta\eta$ in the shell region towards the solvent was used in most cases. In cases, where the form factor model of a core-shell sphere with the same $\Delta\eta$ profile resulted in a better fit, resulting size parameters are displayed with open symbols in addition to parameters obtained upon assumption of a core-shell cylinder. The core radius is distributed according to a log-normal distribution. The parameter r_{core} is the mean value of this distribution and $SD(r_{core})$ the standard deviation of r_{core}.

By comparison of χ^2_{red} (**Table S3**) it is easily seen, that most experimental SANS curves are better described assuming a cylindrical rather than a spherical morphology of the overall assembly. Noteworthy, standard deviations of r_{core} from the spherical model are higher than from the cylindrical model in those cases where the cylindrical model describes experimental data better than the spherical model does. This confirms, that high polydispersity of the core radius points towards anisometric micelles. The cylindrical morphology ($P_{core\text{-}shell\text{-}cylinder}(q)$) was considered to be the better description than the spherical morphology ($P_{core\text{-}shell\text{-}sphere}(q)$) in cases, where χ^2_{red} is lower. The sample with [Blue^{2-}] = 2 mM poses an exception to this criterion due to the high

SD(r_{core}) resulting from fitting upon assumption of a spherical morphology. A cylindrical morphology was therefore considered to be more appropriate in this case.

Based on these findings, cross section dimensions obtained from the fit with a cylindrical form factor were used for the analysis of the entire *q*-regime for all samples with pD ≤ 9 and for samples with [Blue] ≤ 2 mM for pD = 12 and pD = 13 and the spherical form factor for the analysis of the total *q*-regime of the SANS curves emerging from samples containing [Blue] ≥ 6.25 mM at pD ≥ 12.

Table S3: χ^2_{red} values obtained from the application of two model form factors to the high-*q* region (q > 0.045 Å^{-1}) of full contrast SANS curves from samples containing $hC_{12}hE_5$ at a concentration of 25 mM together with the standard deviation *SD*(r_{core}) of r_{core} resulting from the number-weighted log-normal distribution of the core radius. SANS curves were recorded at variable concentrations of Blue and at variable pD. .

	$P_{core-linshell-sphere}(q)$		$P_{core-linshell-cylinder}(q)$ total length L = 90 Å (L_{core} ≈ 66 Å)	
[Blue] / mM	χ^2_{red}	*SD*(r_{core})	χ^2_{red}	*SD*(r_{core})
0	7.75	5.6 ± 0.2	**2.05**	3.45 ± 0.06
		pD = 2		
1	4.09	5.4 ± 0.3	**1.05**	3.6 ± 0.2
2	5.03	5.5 ± 0.3	**1.136**	3.7 ± 0.2
		pD = 9		
1	4.14	5.3 ± 0.3	**0.920**	3.5 ± 0.2
2	3.96	5.3 ± 0.3	**0.741**	3.5 ± 0.2
6.25	3.65	5.1 ± 0.3	**1.14**	3.4 ± 0.2
12.5	3.83	5.0 ± 0.2	**3.05**	2.91 ± 0.09
		pD = 12		
1	5.57	5.3 ± 0.3	**1.57**	3.4 ± 0.1
2	4.75	5.2 ± 0.2	**4.48**	3.1 ± 0.1
6.25	**2.18**	3.98 ± 0.07	2.30	3.77 ± 0.04
12.5	**2.89**	3.76 ± 0.07	3.31	3.6 ± 0.1
		pD = 13		
1	6.18	5.3 ± 0.2	**2.09**	3.3 ± 0.1
2	**2.35**	5.0 ± 0.2	6.47	2.8 ± 0.1
6.25	**2.68**	3.72 ± 0.07	2.80	3.6 ± 0.2
12.5	**3.00**	3.55 ± 0.07	3.20	3.4 ± 0.2

Analysis of the entire *q*-range. The second step of analysing experimental SANS curves addresses the entire *q*-range. For this purpose, form factors $P(q)$ of end-capped core-shell cylinders with a core length L_{core} of either 20 Å, 40 Å, 66 Å, 100 Å or 200 Å and the form factor of a core-shell sphere were systematically applied to all SANS curves (main text, Figure 3B). Ornstein-Zernike scattering was included by substituting $S(q)$ in **eq (SI2)** by the Ornstein-Zernike expression **eq (SI3)**. Unlike to the form factors applied for the analysis of the high-*q* regime, the form factors used in the analysis of the entire *q*-range have a shell with a homogeneous scattering length density (η_{shell}). The choice of these slightly simpler models offered the opportunity to combine them with an Ornstein-Zernike scattering contribution or a structure factor using the SASfit software, which would not have been possible with the model of an end-capped core-shell cylinder or a core-shell sphere with linearly decaying $\Delta\eta$ in the shell (version 0.94.12).

The core radius r_{core} and its distribution were kept constant at values established with fits in step 1. The shell thickness r_{shell}, scattering length densities η_{core} and η_{shell} and Ornstein-Zernike parameters were fitted for each of the described form factor models. The number density of assemblies n_p was calculated from the micellar aggregation as described on p.6 of the SI.

Figure S5 to **Figure S8** display experimental SANS curves and best fits upon application of the form factor of a core-shell sphere (blue, dashed line), the form factor of an end-capped core-shell cylinder with a core length of 40 Å (red, dotted line), and the form factor of an end-capped core-shell cylinder with a core-length of 66 Å (red, solid line). Fits obtained from application of the end-capped core-shell cylinders with a core length of either 20 Å, 100 Å or 200 Å are not shown. Except for fits to SANS curves from samples containing [Blue] ≥ 6.25 at pD ≥ 12, Ornstein-Zernike scattering was included as $S(q)$. Use of Ornstein-Zernike scattering for the interpretation of SANS curves from samples containing [Blue] ≥ 6.25 at pD ≥ 12 did not reveal satisfying results. Therefore, the blue, dotted lines in **Figure S7** and **Figure S8** display best fits with the form factor of core-shell spheres while keeping $S(q) = 1$. The latter fit was improved by the inclusion of a structure factor based on hard sphere interactions (lime green, solid lines).

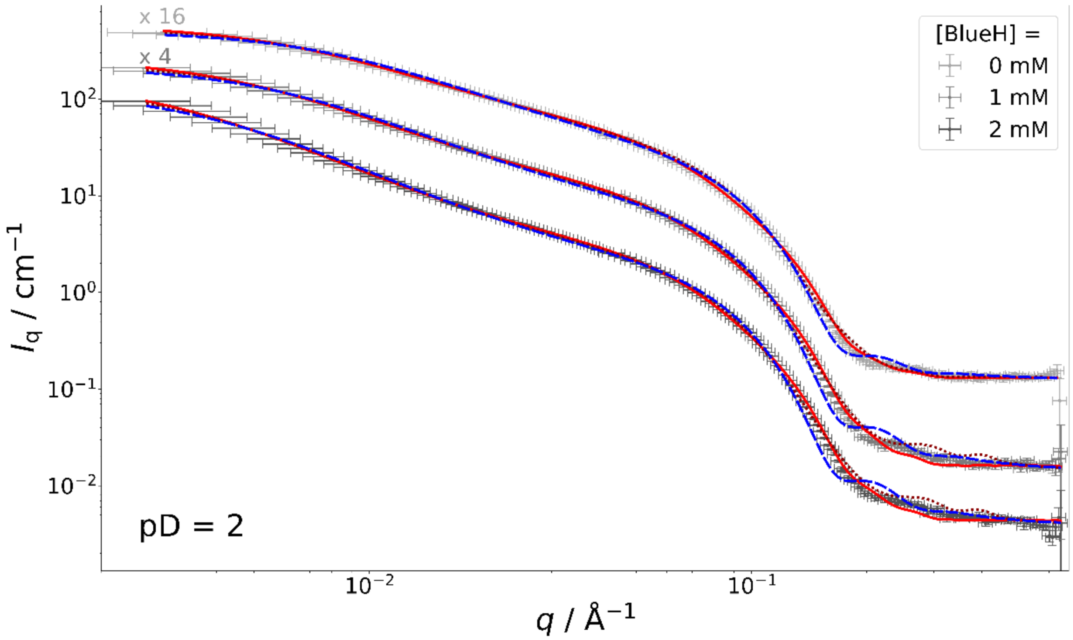

Figure S5: Full contrast SANS curves of solutions containing [$hC_{12}hE_5$] = 25 mM and Blue at the indicated concentration at pD = 2. An isotonic NaCl solution (I = 0.154 M) in D_2O served as the solvent. SANS curves were recorded at a sample temperature of 10 °C. Red line (—): Fit with the form factor of end-capped core-shell cylinders with a core length of L_{core} = 66 Å and Ornstein-Zernike scattering. Red dotted line (**.....**): Fit with the same model, but L_{core} = 40 Å. Blue dashed line (**-----**): Fit with the form factor of core-shell spheres and Ornstein-Zernike scattering.

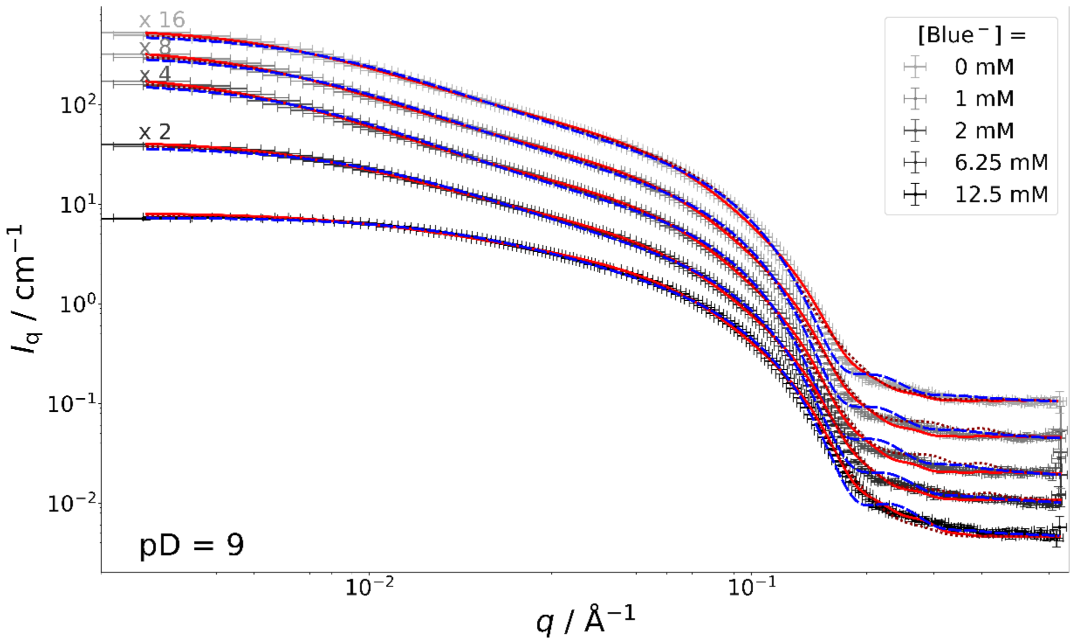

Figure S6: Full contrast SANS curves of solutions containing [$hC_{12}hE_5$] = 25 mM and Blue at the indicated concentration at pD = 9. An isotonic NaCl solution (I = 0.154 M) in D_2O served as the solvent. SANS curves were recorded at a sample temperature of 10 °C. Red line (—): Fit with the form factor of end-capped core-shell cylinders with a core length of L_{core} = 66 Å and Ornstein-Zernike scattering. Red dotted line (**.....**): Fit with the same model, but L_{core} = 40 Å. Blue dashed line (**-----**): Fit with the form factor of core-shell spheres and Ornstein-Zernike scattering.

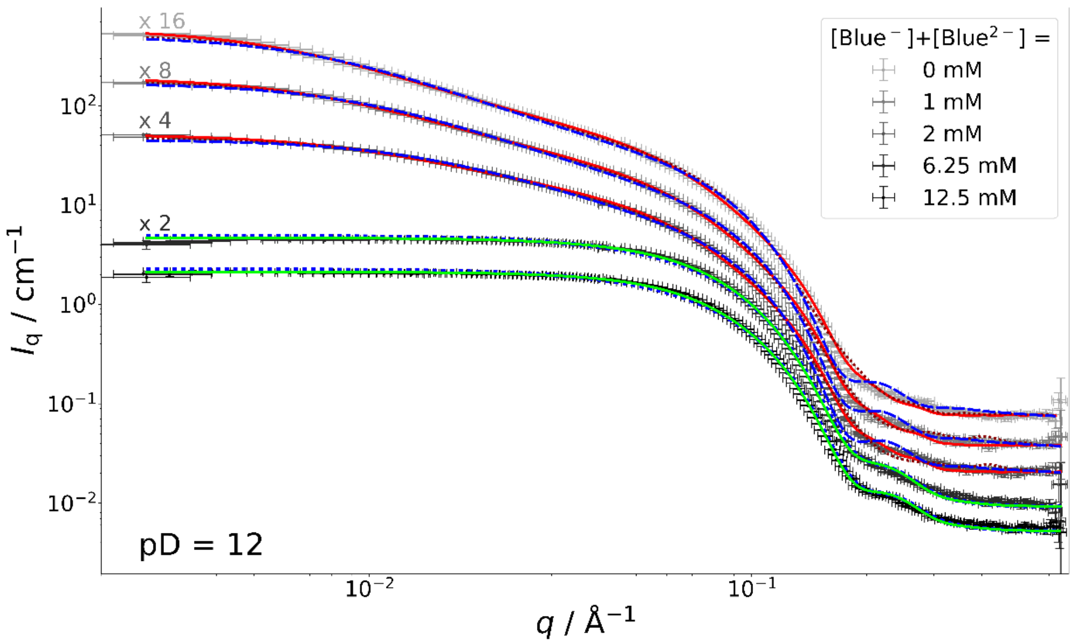

Figure S7: Full contrast SANS curves of solutions containing [$hC_{12}hE_5$] = 25 mM, and Blue at the indicated concentration at pD = 12. An isotonic NaCl solution (*I* = 0.154 M) in D_2O served as the solvent. SANS curves were recorded at a sample temperature of 10 °C. Red line (—): Fit with the form factor of end-capped core-shell cylinders with a core length of L_{core} = 66 Å and Ornstein-Zernike scattering. Red dotted line (**.....**): Fit with the same model, but L_{core} = 40 Å. Blue dashed line (**-----**): Fit with the form factor of core-shell spheres and Ornstein-Zernike scattering. Blue dotted line (**.....**, [Blue] ≥ 6.25 mM): Fit with the form factor of core-shell spheres. Green line (—, [Blue] ≥ 6.25 mM): Fit with the form factor of core-shell spheres and a hard sphere structure factor.

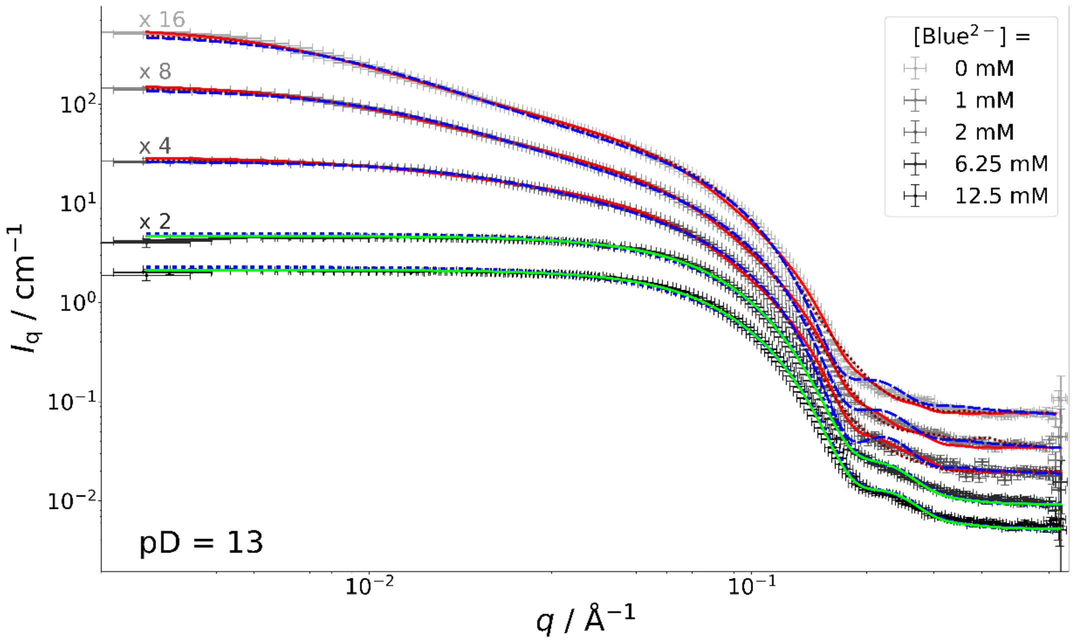

Figure S8: Full contrast SANS curves of solutions containing [$hC_{12}hE_5$] = 25 mM, and Blue at the indicated concentration at pD = 13. An isotonic NaCl solution (*I* = 0.154 M) in D_2O served as the solvent. SANS curves were recorded at a sample temperature of 10 °C. Red line (—): Fit with the form factor of end-capped core-shell cylinders with a core length of L_{core} = 66 Å and Ornstein-Zernike scattering. Red dotted line (**.....**): Fit with the same model, but L_{core} = 40 Å. Blue dashed line (**-----**): Fit with the form factor of core-shell spheres and Ornstein-Zernike scattering. Blue dotted line (**.....**, [Blue] ≥ 6.25 mM): Fit with the form factor of core-shell spheres. Green line (—, [Blue] ≥ 6.25 mM): Fit with the form factor of core-shell spheres and a hard sphere structure factor.

In order to deduce an overall morphology of the assembly, the fit quality was compared in the mid- and low-q regime (q < 0.134 Å^{-1}) by means of χ^2_{red}. Excluding the high-q regime from this analysis avoids distortions due to the use of a simplified scattering length density profile for the cross section, which mostly affects the high-q region. **Table S4** compares χ^2_{red} values obtained from the application of different form factors to the mid- and low-q regime (q < 0.134 Å^{-1}) of experimental SANS curves. In cases, where fitting did not provide meaningful parameters upon inclusion of Ornstein-Zernike scattering no χ^2_{red} values are given.

Table S4: χ^2_{red} values obtained from fits to the mid- and low-q regime (q < 0.134 Å^{-1}) of full contrast SANS curves. Solutions. Solutions were prepared in an isotonic NaCl solution at [NaCl]=154 mM in D_2O had a concentration of $hC_{12}hE_5$ of 25 mM and a temperature of 10 °C. The product of a core-shell particle form factor and an Ornstein-Zernike scattering contribution was applied in all cases. In cases, where the form factor of an end-capped core-shell cylinder was applied, the fixed core length is given by L_{core}. No χ^2_{red} values are given in cases, where fits including Ornstein-Zernike scattering did not improve χ^2_{red} compared to fits without the Ornstein-Zernike contribution.

[Blue] / mM	χ^2_{red} $P_{\text{core-shell-sphere}}(q)$	χ^2_{red} $P_{\text{cap-core-shell-cylinder}}(q)$ L_{core} = 20 Å	χ^2_{red} $P_{\text{cap-core-shell-cylinder}}(q)$ L_{core} = 40 Å	χ^2_{red} $P_{\text{cap-core-shell-cylinder}}(q)$ L_{core} = 66 Å	χ^2_{red} $P_{\text{cap-core-shell-cylinder}}(q)$ L_{core} = 100 Å	χ^2_{red} $P_{\text{cap-core-shell-cylinder}}(q)$ L_{core} = 200 Å
0	56.2	139.781	59.5	**28.5**	39.1	59.3168
			pD = 2			
1	109.8	74.1	38.3	**15.6**	59.6	111.9
2	143.6	90.7	48.0338	**19.1**	69.8	147.8
			pD = 9			
1	103.5	70.8	36.5	**15.5**	59.8	107.0
2	125.7	87.3	46. 2	**14.6**	51.6	107.9
6.25	54.0	32.3	**15.2**	15.8	57.8	75.2
12.5	18.9	4.69	**3.34**	25.9	66.0	-
			pD = 12			
1	47.1	24.3	**11.5**	20.9	67.3	80.9
2	29.3	11.2	**4.72**	26.0	87.3	142.5
6.25	-	-	-	-	-	-
12.5	-	-	-	-	-	-
			pD = 13			
1	37. 4	16.8	**8.02**	22.5	66.7	77.0
2	8.22	1.84	**1.79**	15.0	59.0	140
6.25	-	-	-	-	-	-
12.5	-	-	-	-	-	-

Fitted parameters are displayed in **Figure S9** for fits, where the form factor of an end-capped core-shell cylinder with a core length L_{core} of either 40 Å or 66 Å was applied. Correlation lengths of concentration fluctuations ξ from the fits are displayed in **Figure 5** of the main text.

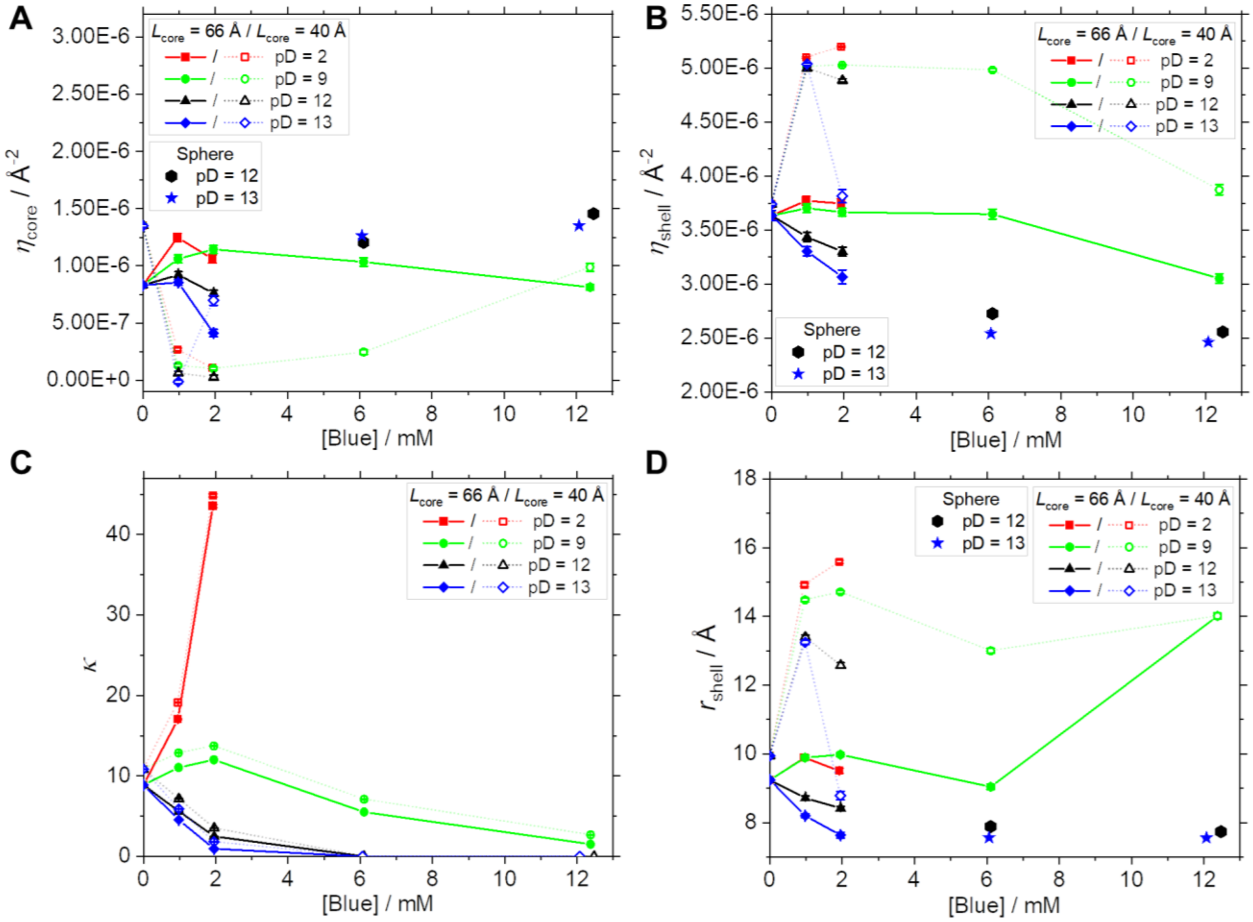

Figure S9: **A** Scattering length density (η_{core}) of the core, **B** scattering length density (η_{shell}) of the shell, **C** the scaling parameter for Ornstein-Zernike scattering (κ) and **D** the shell thickness (r_{shell}) obtained from fitting the entire q-range of full contrast SANS curves shown in **Figures S5-S8**. Fits were performed with the form factor for end-capped core-shell cylinders with a core length (L_{core}) of either 40 Å or 60 Å. Ornstein-Zernike scattering was included into the analysis. Correlation lengths are shown in Figure 5A of the main document. Cases, where the application of the core-shell sphere form factor with a hard sphere structure factor resulted in a better fit are separately indicated. The core radius r_{core} and its distribution were adopted from the corresponding high-q fit (**Figure S4**) in all cases.

SI4. Dependence of $C_{12}E_5$ self-assembly on solution pD

SANS curves were recorded from solutions of $hC_{12}hE_5$ at three different pD values at the surfactant concentration of 25 mM used throughout the present study. The pD values were 2, 9 and 13 thereby covering the entire pD regime relevant for the present study. As these SANS curves, shown in **Figure S10**, perfectly overlay, they were fitted globally with the form factor model of end-capped core-shell cylinders with a core length of 66 Å including fluctuation scattering via an Ornstein-Zernike term. Details of the fit are explained in the main document and in Section SI3 of the SI. The identity of SANS curves from $C_{12}E_5$ solutions with different pD proves, that the morphology of $C_{12}E_5$ micelles does not depend on solution pD under the given conditions.

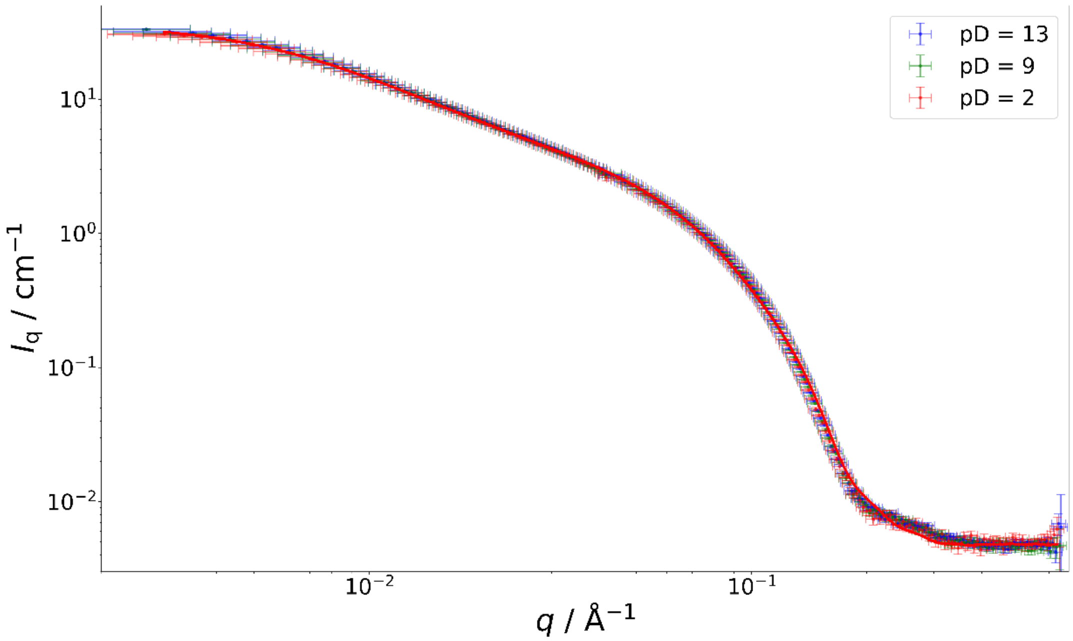

Figure S10: Full contrast SANS curves of solutions containing [$hC_{12}hE_5$] = 25 mM at pD = 2, 9, and 13. An isotonic NaCl solution at [NaC] = 154 mM in D_2O was used as the solvent. SANS curves were recorded at a sample temperature of 10 °C. The red line (—) displays a fit with the form factor model of end-capped core-shell cylinders with a core length of 66 Å including fluctuation scattering as a structure factor. Experimental SANS curves and the fit overlay perfectly.

SI5. Light scattering – Data Evaluation

Combined static and dynamic light scattering (SLS/DLS) was applied to investigate isotope effects due to variable deuteration of $C_{12}E_5$ (**Section SI6**) and to determine the correlation lengths of concentration fluctuations ξ in solutions of Blue-$C_{12}E_5$ mixtures supplementary to SANS experiments (**Section SI7**).

Samples for light scattering were prepared by the same procedure as NMR- and SANS samples, which is described in the experimental section of the main document. Samples for light scattering were filtered (MACHEREY-NAGEL, CHROMAFIL Xtra H-PTFE syringe filters, pore size 0.2 µm) into tempered, dust-free cuvettes at a temperature of 7 °C after an equilibration time of at least 4 h at that temperature and subsequently stored for 24 h.

Light scattering measurements were performed on an ALV CGS-3 Compact Goniometer System (ALV GmbH, Langen, FRG) using a HeNe laser at a wavelength of 632.8 nm. Measurements were performed using cylindrical quartz glass cuvettes with an inner diameter of 1 cm. The temperature of the sample in the toluene bath was controlled using a thermostat set to 10 °C. Samples were equilibrated at that temperature in the toluene bath for at least 1 h prior to any light scattering measurement. For the evaluation of static light scattering (SLS) data, toluene was used as a standard.

Static light scattering. The absolute intensity scattered by the sample at a given angle θ is given by the Rayleigh ratio (R_θ) and calculated according to the following equation:[9]

$$R_\theta = \frac{I_\theta - I_{\mathrm{solvent},\theta}}{I_{\mathrm{toluene},\theta}} \cdot R_{\mathrm{toluene}} \qquad \text{(SI4)}$$

In **eq (SI4)**, I_θ is the experimentally measured scattered intensity from the solution at the scattering angle θ. $I_{\mathrm{solvent},\theta}$ and $I_{\mathrm{toluene},\theta}$ are the scattering intensities of the solvent and the standard toluene at the same angle and R_{toluene} is the absolute scattering intensity of the standard toluene.

The q-dependency of the Rayleigh ratio was analysed under the assumption of fluctuation scattering and is described by the Ornstein-Zernike relation:[10,11,12]

$$R_\theta = \frac{R_0}{1 + q^2\xi^2} \qquad \text{(SI5)}$$

In **eq (SI5)**, R_0 is the zero- or forward scattering intensity. It is related to the isothermal osmotic compressibility χ_T according to[7,12]

$$R_0 = AT\chi_T \tag{SI6}$$

where A is an instrumental constant and T the temperature. The validity of the Ornstein-Zernike relation can be verified by recovering a linear relation between the inverse scattering intensity R_θ^{-1} and the squared scattering vector q^2 in an Ornstein-Zernike.[12]

$$\frac{1}{R_\theta} = \frac{1}{R_0} \cdot \left(1 + \xi^2 q^2\right) \tag{SI7}$$

Extrapolation of $1/R_\theta$ to $q = 0$, reveals the forward scattering intensity R_0 and the correlation length of concentration fluctuations ξ .

Dynamic light scattering. In a dynamic light scattering experiment, the normalized intensity time autocorrelation function $g^{(2)}(\tau)$ is measured as a function of the correlation time at given scattering vector q.[9,13] Cumulant analysis provides a suitable tool to obtain the average decay rate $\overline{\Gamma}$ of $g^{(2)}(\tau)$, which relates to the apparent diffusion coefficient D_{app} at a given angle according to

$$\overline{\Gamma} = D_{app} \cdot q^2 \tag{SI8}$$

Assuming a monomodal, but polydisperse distribution of $\overline{\Gamma}$, $g^{(2)}(\tau)$ can directly be fitted with the following expression:[13]

$$g^{(2)}(\tau) = B + \beta \exp\left(-2\overline{\Gamma}\tau\right)\left(1 + \frac{\mu_2}{2!}\tau^2\right)^2 \tag{SI9}$$

In **eq (SI9)**, $\overline{\Gamma}$ is the average decay rate and μ_2 its variance, leading to the variance of D_{app}

$$Var\left(D_{app}\right) = \left(\frac{1}{q^2}\right)^2 \cdot \mu_2 \tag{SI10}$$

In cases, where no clear q-dependency of D_{app} was observed, the diffusion coefficient D was obtained as an average of all D_{app}. The variance of D was obtained from error propagation as $Var(D) = Z^{-2} \cdot \sum Var(D_{app})$ with Z being the number of D_{app} values to be averaged. As each D_{app} was obtained from a separate fit, no covariance between D_{app} obtained at different angles was included. The standard deviation of the diffusion coefficient $SD(D)$ is $\{Var(D)\}^{0.5}$.

If D_{app} depends on q, the diffusion coefficient D is typically obtained from an extrapolation of D_{app} as a function of q^2 to $q = 0$.

$$D_{app}(q) = D \cdot \left(1 + K \cdot \langle R_g^2 \rangle_z q^2\right) \tag{SI11}$$

In **eq (SI11)**, $\langle R_g^2 \rangle_z$ is the *z*-average radius of gyration of scattering particles and K depends on sample polydispersity as well as on the particle topology.[9]

An alternative approach to analyse dynamic light scattering data is to perform an inverse Laplace Transformation of the field autocorrelation function. The field-time autocorrelation function $g^{(1)}(\tau)$ is related to the intensity-time autocorrelation function $g^{(2)}(\tau)$ via the Siegert relation.[9,13]

$$g^{(2)}(\tau) = 1 + \left[g^{(2)}(\tau = 0)\right] \cdot \left|g^{(1)}(\tau)\right|^2 \qquad \text{(SI12)}$$

One way to perform an inverse Laplace Transformation on $g^{(1)}(\tau)$ is to use the constrained regularization method (CONTIN) developed by Provencher.[12,14,15]

The diffusion coefficient in the present system depends on the motion of polydisperse $C_{12}E_5$ micelles and on concentration fluctuations. Due to the similarity of micellar sizes and the correlation length of concentration fluctuations, a distinction between these two contributions is not possible with the performed experiments. Therefore, D was used to calculate an apparent hydrodynamic size R_a using the Stokes-Einstein equation:[11,16]

$$R_a = \frac{k_B T}{6\pi \eta D} \qquad \text{(SI13)}$$

In **eq (SI13)**, k_B is the Boltzmann constant, T is the temperature and η is the viscosity of the solvent. The distance parameter R_a was used to compare relative differences of samples analysed within this work, keeping in mind that an increase in R_a could signal an increase in particle size and/or in correlation length of concentration fluctuations (ξ).

SI6. Isotope effects

Figure S11 shows the clouding temperatures CT of solutions of $C_{12}E_5$ at $[C_{12}E_5]$ = 25 mM containing Blue at varying concentration and at variable pD for samples prepared with $mC_{12}mE_5$ which is a mixture of $mC_{12}hE_5$ and $mC_{12}dE_5$ at a volume ratio of 18.1:81.9 (left plot) in comparison to samples prepared with $hC_{12}hE_5$ (right plot). All solutions were prepared in D_2O. Clouding temperatures in both graphs follow the same trends concerning their dependence on pD and on Blue concentration, regardless of whether solutions contain $hC_{12}hE_5$ or $mC_{12}mE_5$. However, samples containing $mC_{12}mE_5$, systematically show an increase of CT by 6 – 7°K. This deviation is attributed to isotope effects. In literature, similar observations of the CT of C_nE_m systems were reported when the H_2O solvent was substituted for D_2O, which resulted in an increase of the CT by about 3 – 5 K.[7]

According to literature data and results reported in the main document (**Figure 5B**), a CT increase goes along with a reduction in the correlation length of concentration fluctuations (ξ) and/or the size of micelles at a given temperature.[10,11,17] Therefore, ξ and the size of micelles are expected to be smaller for $mC_{12}mE_5$ compared to $hC_{12}hE_5$.

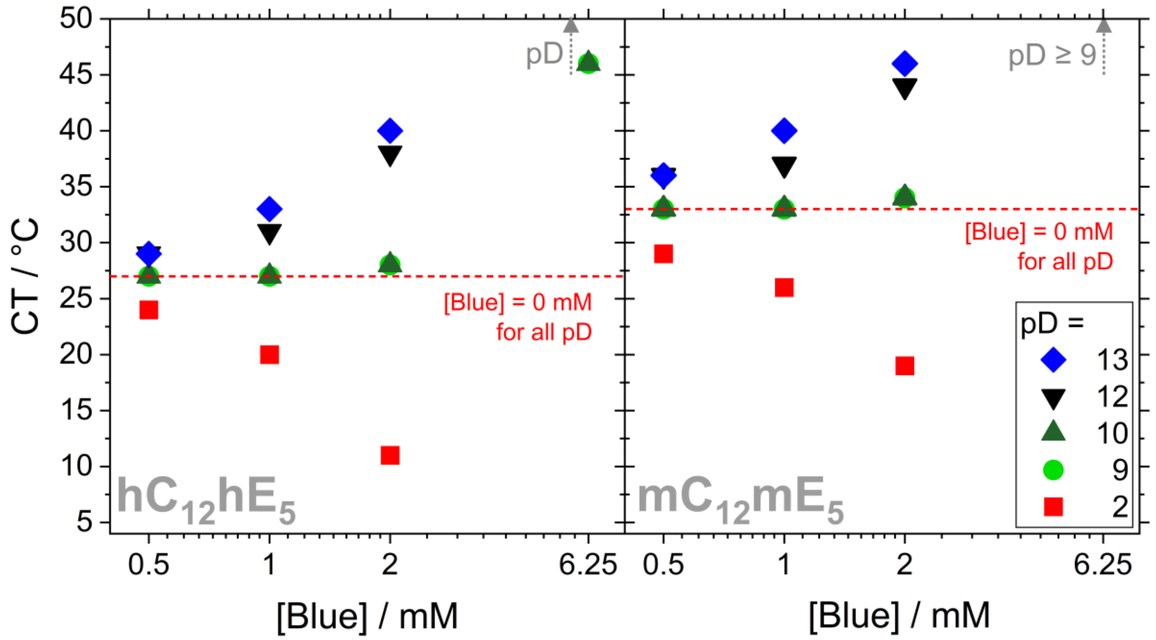

Figure S11: CT of solutions in D_2O, containing either completely hydrogenated surfactant $hC_{12}hE_5$ at a concentration of $[hC_{12}hE_5]$ = 25 mM (left) or a mixture of $mC_{12}hE_5$ and $mC_{12}dE_5$ at match point composition ($mC_{12}mE_5$) at a concentration of $[mC_{12}mE_5]$ = 25 mM (right) at variable concentration of Blue and pD. An isotonic NaCl solution (I = 0.154 M) in D_2O was used as a solvent. The pD of each solution was adjusted with solutions of DCl and NaOD. Grey, dotted arrows indicate, that CT of solutions containing [Blue] = 6.25 mM at pD ≥ 12 ($hC_{12}hE_5$) and at pD ≥ 9 ($mC_{12}mE_5$) lay above 50 °C. CT of solutions containing [Blue] = 12.5 mM at pD ≥ 9 lay above 50 °C in case of both surfactants.

As $mC_{12}mE_5$ is a mixture of $mC_{12}hE_5$ and $mC_{12}dE_5$, its self-assembly and CT-properties likely lay between that of $mC_{12}hE_5$ and $mC_{12}dE_5$. For this reason and to investigate trends as a function of the degree of deuteration of the surfactant, solutions of pure $mC_{12}hE_5$ and pure $mC_{12}dE_5$ were investigated and results compared to the behaviour $hC_{12}hE_5$ in solution.

Figure S12 shows DLS intensity correlation functions of samples containing either $hC_{12}hE_5$, $mC_{12}hE_5$ or $mC_{12}dE_5$ at a concentration of 25 mM. It is clearly visible, that the decay of $g^{(2)}(\tau)$ is shifted to smaller correlation times for an increasing degree of deuteration, pointing towards smaller micelles. Intensity correlation functions were fitted with the revised method of cumulants (eq (SI9)), which permitted to obtain an apparent diffusion coefficient D_{app} and its variance (eq (SI10)) for each angle. As D_{app} did not show a pronounced angular dependency, all values of D_{app} were averaged to obtain the diffusion coefficient D. Error propagation yielded the variance and therefore also the standard deviation SD of D. The values of D and $SD(D)$, together with an apparent hydrodynamic size R_a calculated according to **eq (SI13)** and its standard deviation are displayed in **Table S5**. The standard deviations of D and R_a are a measure of the width of the size distribution and/or distribution of correlation lengths of $C_{12}E_5$ micelles. An idea about the width of such a distribution may also be obtained by calculating the distribution ($A(\tau)$) of decay times from the field correlation function $g^{(1)}(\tau)$ using CONTIN analysis. Such distributions are displayed in **Figure S12**. They have similar width, but, depending on the extent of deuteration of the $C_{12}E_5$ species, are shifted along the τ-axis, in line with the trend of diffusion coefficients D and their standard deviation $SD(D)$ displayed in **Table S5**. With increasing degree of deuteration, an increase in D is observed, leading to a decrease of the hydrodynamic size.

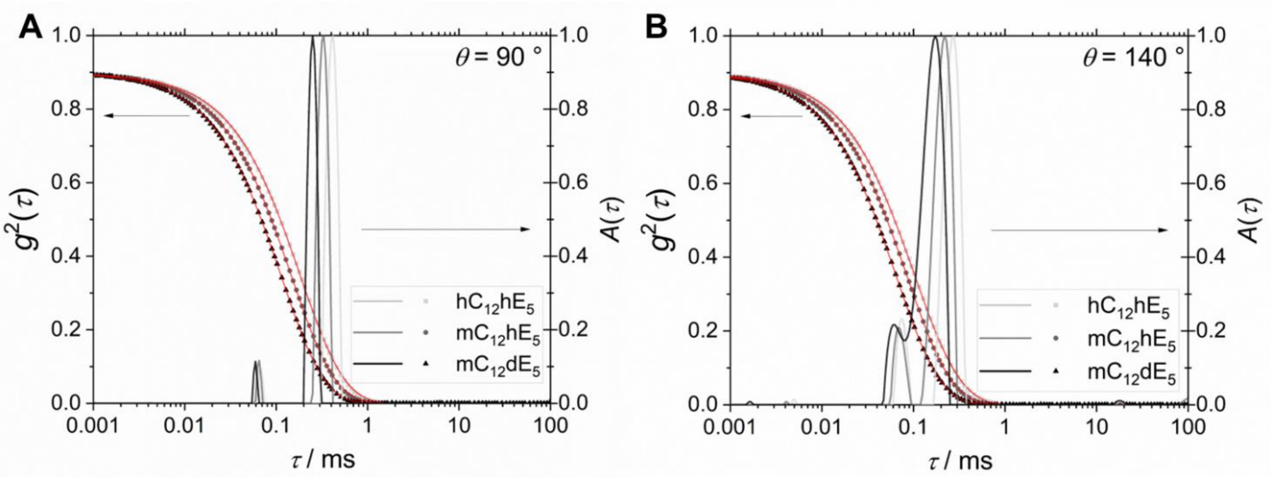

Figure S12: Intensity autocorrelation functions $g^{(2)}(\tau)$ of samples containing the indicated $C_{12}E_5$ species at a concentration of $[C_{12}E_5]$ = 25 mM in an isotonic NaCl solution prepared in D_2O at pD = 2 and 10 °C at the scattering angles θ = 90 ° (**A**) and θ = 140 ° (**B**). The red lines show cumulant fits to $g^{(2)}(\tau)$ (eq (SI9)). Solid, grey lines display the distribution of decay times $A(\tau)$ obtained from CONTIN analysis of respective field autocorrelation functions $g^{(1)}(\tau)$.

Figure S13 displays Ornstein-Zernike plots of SLS data from solutions of the three differently deuterated $C_{12}E_5$-species. They were evaluated according to **eq (SI7)** to obtain correlation lengths of concentration fluctuations ξ(SLS) displayed in **Table S5**. Apart from the linear dependency of R_θ^{-1} on q^2, a small upturn of R_θ^{-1} is observed at low values of q^2. In the $R_\theta = f(q)$ scattering curve this translates to a correlation peak, which appears due to spatial correlations with a correlation length of about $2\pi q_{\text{peak}}^{-1} \approx 564$ Å ($q_{\text{peak}} \approx 0.011$ Å^{-1}). This value is larger than the correlation lengths ξ(SLS) found for the concentration fluctuations. No physical explanation can yet be presented for the appearance of the correlation peak. Even though this additional feature slightly impairs an Ornstein-Zernike analysis of SLS data according to **eq (SI7)**, ξ(SLS) is still considered to be comparable among each other due to the low strength of the correlation peak.

According to the analysis of SLS data, correlation lengths of concentration fluctuations become smaller with increasing degree of deuteration of the surfactant. This is consistent with the observed increase in the CT of solutions containing deuterated surfactant compared to $hC_{12}hE_5$. In qualitative agreement with SLS, R_a from DLS analysis yield a similar trend.

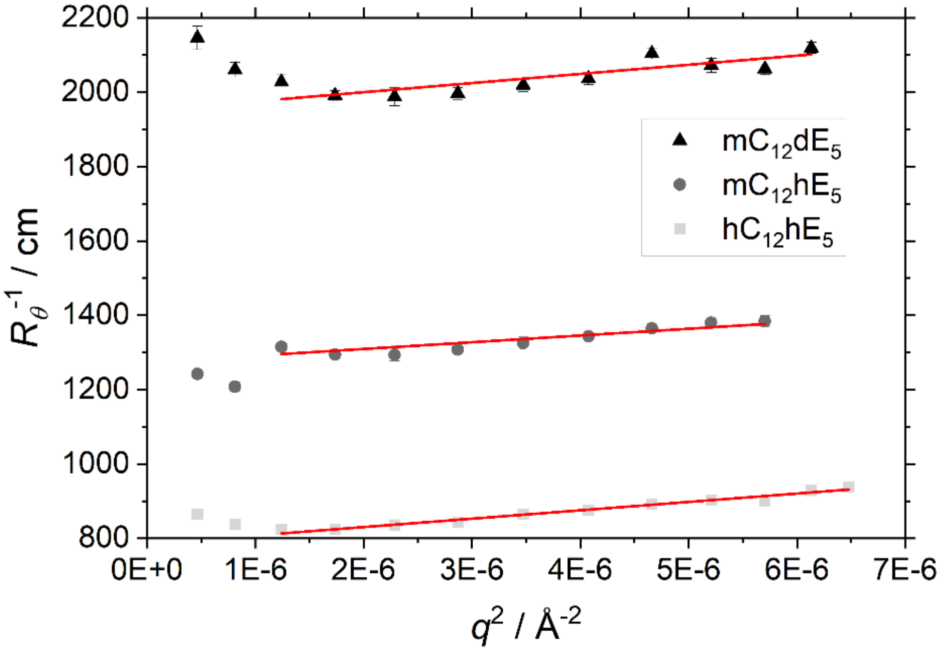

Figure S13: Ornstein-Zernike plots of SLS data from $C_{12}E_5$ solutions according to eq (SI7) and corresponding fits. The reciprocal Rayleigh ratio is depicted as a function of the square of the value of the scattering vector. Sample solutions contained the respective $C_{12}E_5$ species at a concentration of [$C_{12}E_5$] = 25 mM and were prepared in an isotonic NaCl solution in D_2O with pD = 2. Measurements were performed at a sample temperature of 10 °C. The red lines denote data evaluation according to **eq (SI7)**.

Table S5: Comparison of the size and Ornstein-Zernike scattering parameters of micelles formed by $C_{12}E_5$ molecules with different degrees of deuteration at $[C_{12}E_5]$ = 25 mM in an isotonic solution of NaCl in D_2O at pD = 2 and 10 °C. Cross section dimensions were obtained from fitting the high-q region with the form factor of core-shell cylinders with a linearly decaying contrast towards the solvent in the shell region at a fixed length of 90 Å. The corresponding profile of the scattering length density difference $\Delta\eta$ is given in the first row. For $hC_{12}hE_5$, a log-normal distribution of the core radius was assumed. For this reason, r_{core} is the mean value of this distribution and $SD(r_{core})$ its standard deviation. Parameters for the Ornstein-Zernike scattering contribution were obtained from fitting the entire SANS curve with the form factor of an end-capped core-shell cylinder with a core length of L_{core} = 66 Å.

	$hC_{12}hE_5$	$mC_{12}hE_5$	$mC_{12}dE_5$
**SANS**			
$\Delta\eta$ profile	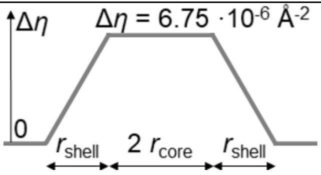	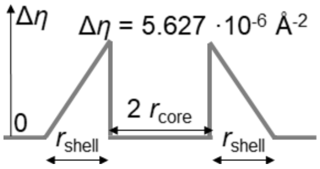	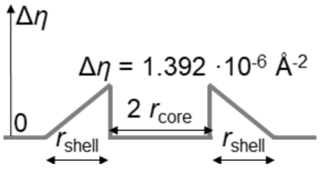
r_{core} / Å	13.3 ± 0.2	11.2 ± 0.2	15.3 ± 2
$SD(r_{core})$ / Å	3.45 ± 0.06<br>Log-normal distribution	0	0
r_{shell} / Å	13.1 ± 0.2	20.9 ± 0.3	12 ± 4
κ(SANS)	8.88 ± 0.02	5.58 ± 0.07	35.3 ± 11
ξ(SANS) / Å	140 ± 0.2	119 ± 2	418 ± 76
**SLS**			
R_0(SLS) / 10^{-6} cm^{-1}	1273 ± 9	785 ± 7	513 ± 6
ξ(SLS) / Å	169 ± 7	119 ± 13	112 ± 14
**DLS**			
D / 10^6 Å^2 s^{-1}	788	986	1277
$SD(D)$ / 10^6 Å^2 s^{-1}	105	123	151
R_a / Å	158	126	97
$SD(R_a)$ / Å	21	16	12

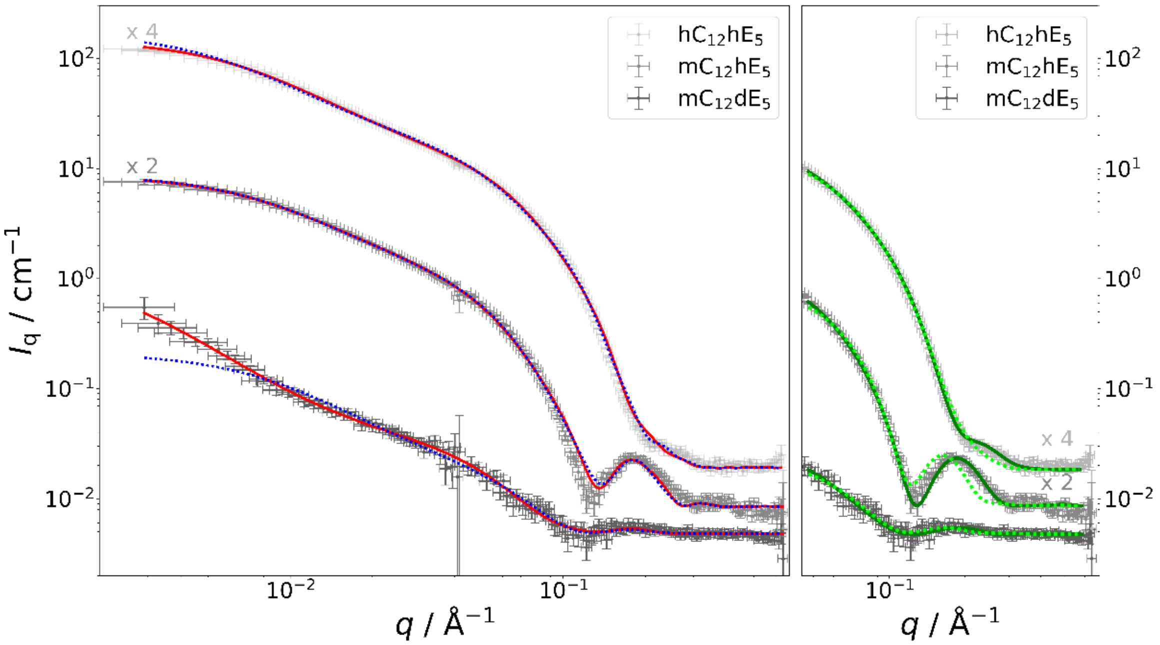

Figure S14: SANS curves of solutions from three different $C_{12}E_5$ species at a concentration of 25 mM and the indicated pD. An isotonic NaCl solution with [NaCl]=0.154 M in D_2O was used as the solvent. SANS curves were recorded at a sample temperature of 10 °C. **Left hand**: Red line (—) : Fit with the form factor model of end-capped core-shell cylinders with a core length of 66 Å including Ornstein-Zernike scattering as a structure factor. Dotted, blue line (....): Fit with the same form factor model including Ornstein-Zernike scattering as the structure factor, but the correlation length ξ was kept constant to values obtained from the evaluation of static light scattering data in the Ornstein-Zernike plot (**Figure S13** and **Table S5**). **Right hand**: Dark green line (—): Form factor fit assuming core-shell cylinders with a total length of L = 90 Å and a linearly decaying scattering length density difference ($\Delta\eta$) profile within the shell region of the cylinder cross section. Green dotted line (.....): Form factor fit assuming core-shell spheres and the same $\Delta\eta$ profile. $\Delta\eta$ profiles are displayed in **Table S5**.

Figure S14 displays SANS curves from solutions containing the three differently deuterated $C_{12}E_5$ surfactants. The right image displays the analysis of assembly cross sections analogous to investigations described in the main document and in **Section SI3**. This was done by only fitting the high-q region (q > 0.045 Å^{-1}) with the form factor of a core-shell cylinder or with the form factor of a core-shell sphere. In both cases, the scattering contrast was assumed to linearly decay towards the solvent in the shell region. The profile of the scattering length density difference differs for each surfactant (first row of **Table S5**). In case of a core-shell cylinder a total length of L = 90 Å was used as a pre-set value (see also **Section SI3**). The assumption of a cylindrical morphology provided the best fit in all cases. The resulting cross section dimensions from this analysis are displayed in **Table S5**. For fitting the SANS curve from solution of $hC_{12}hE_5$, a log-normal distribution of the core radius needed to be assumed to describe the data well. The standard

deviation $SD(r_{core})$ of r_{core} calculated from this distribution is displayed in **Table S5** and r_{core} denotes the mean value of the distribution. For $mC_{12}hE_5$ no such distribution needed to be assumed, which indicates that the necessity to assume a distribution of r_{core} results from the small difference between the scattering length densities of the core and the shell for $hC_{12}hE_5$. For $mC_{12}dE_5$, no distribution of r_{core} was assumed either, which is justified by the high signal-to-noise ratio of the experimental SANS curve. Core radii and shell thicknesses obtained from this analysis differ for all $C_{12}E_5$ species. This is attributed to the different contrast conditions caused by the differing extent of deuteration. Total cross section radii ($r_{core}+r_{shell}$) lay between 26.4 Å and 32.1 Å, but do not systematically vary with the degree of deuteration. As all surfactants possess the same chain lengths in their hydrophobic and their hydrophilic part, the apparent differences in cross section dimensions are likely caused by differences in contrast and actual cross section dimensions are expected to be similar.

Core radii shown in **Table S5** were successively used as fixed values in the fits to the total scattering curves. The left plot in **Figure S14** shows experimental SANS curves together with fits assuming the form factor of end-capped core-shell cylinders with and Ornstein-Zernike scattering. A core length of L_{core} = 66 Å which corresponds to an overall cylinder length of $L \approx 90$ Å revealed the best fit for all three types of $C_{12}E_5$.

From **Table S5** it can be seen, that κ and ξ obtained from this fit differ for differently deuterated $C_{12}E_5$ species. Correlation lengths obtained from SANS are fairly similar to those obtained from SLS for $hC_{12}hE_5$ and $mC_{12}hE_5$. Only in case of $mC_{12}dE_5$ do the two values significantly differ from each other. The dotted, blue line in **Figure S14** shows a fit, for which correlation lengths ξ were kept constant to values obtained from SLS measurements. SANS curves of $hC_{12}hE_5$ and $mC_{12}hE_5$ are well described with this approach. The SANS curve of $mC_{12}dE_5$ is well described up until the low-q upturn in scattering intensity. Considering the consistency of light scattering results with observed CT variations and the fact, that light scattering revealed a systematic decrease in ξ with increasing degree of deuteration, the high correlation length found with SANS for $mC_{12}dE_5$ is questionable.

To conclude this section, isotope effects cause an increase of solution CT when $mC_{12}mE_5$ instead of $hC_{12}hE_5$ is used as a surfactant (**Figure S11**). This goes along with a decrease in the correlation length of concentration fluctuations with increasing degree of surfactant deuteration at a given temperature. However, neither the trends of micellar morphology nor of CT caused by the addition of Blue were affected by deuteration. It was assumed, that the length of cylindrical micelles formed

by $mC_{12}mE_5$ is similar to the length of cylindrical micelles formed by $hC_{12}hE_5$. Cylinder lengths were therefore inferred from the $hC_{12}hE_5$ case and all isotope effects were included into the Ornstein-Zernike parameters ξ and κ. This is fully justified as the main focus of the present analysis lays on the evaluation of the morphology of the micellar cross sections.

SI7. Light scattering from BlueH/$C_{12}E_5$ solutions at pD = 2

SANS data close enough to the CT exhibit concentration fluctuations which can be described by an Ornstein-Zernike term. To confirm this pattern supplementary light scattering experiments were carried out with samples containing $hC_{12}hE_5$ at a concentration of 25 mM and varying concentrations of Blue. An isotonic NaCl solution with [NaCl] = 154 mM in D_2O served as the solvent. All measurements were performed *at* pD = 2 and at a sample temperature of 10 °C. Results from SLS are summarized in **Figure S15**.

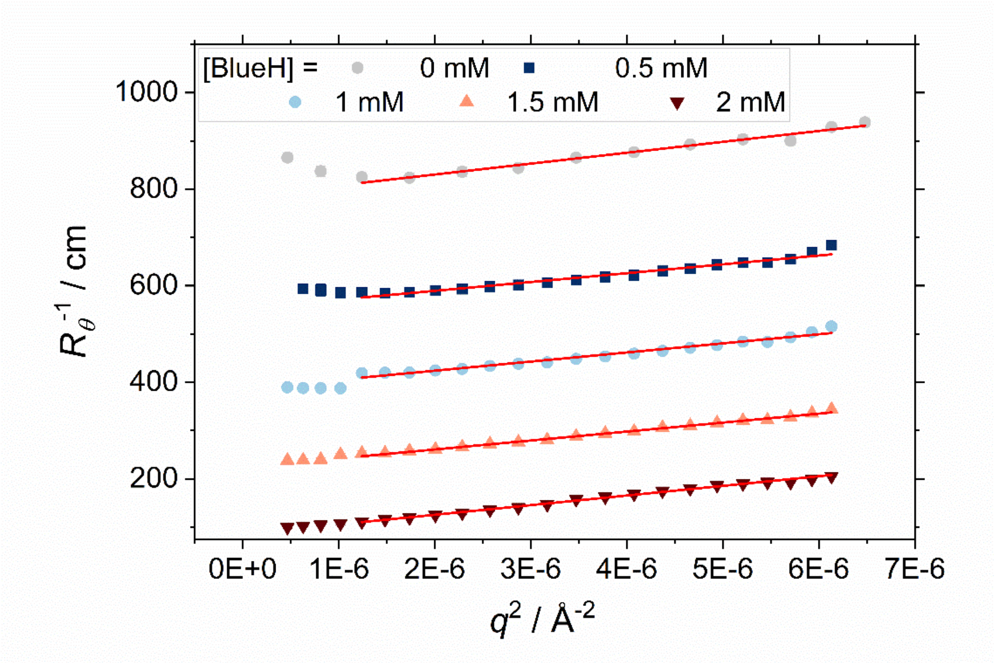

Figure S15: Ornstein Zernike plots of SLS data obtained from solutions containing $hC_{12}hE_5$ at a concentration of 25 mM and variable concentrations of BlueH. An isotonic NaCl solution prepared in D_2O served as the solvent. All measurements were performed at pD = 2 and at a sample temperature of 10 °C. Red lines indicate data evaluation according to **eq (SI7)**.

Correlation lengths for concentration fluctuations were obtained from fits according to **eq (SI7)** and are shown in **Table S6** in comparison to ξ obtained from fitting of full contrast SANS experiments. For the sample containing [Blue] = 1 mM, SANS covered a sufficiently low *q*-range to obtain a correlation length ξ, which is comparable to ξ obtained from SLS. For the sample containing [Blue] = 2 mM, the Guinier plateau was not reached in the *q*–range covered by SANS and the analysis of SLS data provides a more reliable correlation length. Apart from the expected linear relationship between R_θ^{-1} and q^2, **Figure S15** shows an upturn of R_θ^{-1} at low values of q^2. The same observation was made in **Figure S13** and discussed in **Section S6**.

Intensity correlation functions obtained from DLS measurements were analysed using the method of cumulants (**eq (SI9)**). **Figure S16** displays $g^{(2)}(\tau)$ for samples containing $hC_{12}hE_5$ at a concentration of 25 mM and varying concentrations of Blue at a scattering angle of 90 °. The decay of $g^{(2)}(\tau)$ becomes broader with increasing [Blue], but does not show two distinct shoulders. The broad decay points towards a high polydispersity of $C_{12}E_5$ micelles, but does not reveal two distinct species of micelles differing in size. Apparent diffusion coefficients obtained by cumulant analysis at different angles are displayed in **Figure S17** and were fitted according to **eq (SI11)** to obtain diffusion coefficients D, which were used to calculate distances R_a that are displayed in **Table S6**.

Table S6: Comparison of Ornstein-Zernike parameters obtained from fitting full contrast SANS curves to correlation lengths obtained from the evaluation of SLS data and to apparent hydrodynamic sizes R_a obtained from DLS. The Ornstein-Zernike parameters κ and ξ were obtained from fitting full contrast SANS curves in the entire q-range with the form factor for end-capped core shell cylinders with a core length of 66 Å, including Ornstein-Zernike scattering. For [BlueH] = 0.5 mM and [BlueH] = 1.5 mM, no SANS curves exist.

**[BlueH] :**	**0.5 mM**	**1 mM**	**1.5 mM**	**2 mM**
**SANS**				
κ(SANS)	-	17.07 ± 0.06	-	43.6 ± 0.2
ξ(SANS) / Å	-	200.9 ± 0.5	-	322 ± 1
**SLS**				
R_0(SLS) / 10^{-6} cm^{-1}	1809 ± 12	2589 ± 22	4469 ± 28	11682 ± 213
ξ(SLS) / Å	182 ± 7	221 ± 9	288 ± 8	483 ± 35
**DLS**				
R_a / Å	205 ± 1	257 ± 2	359 ± 2	792 ± 16
$K\langle R_g^2\rangle\cdot\xi(\mathrm{SLS})^{-2}$	0.33 ± 0.3	0.51 ± 0.03	0.58 ± 0.02	0.76 ± 0.06

An increase of the slope in $D_{app} = f(q^2)$, which is proportional to $K\cdot\langle R_g^2\rangle$, signals an increase in particle polydispersity and/or size, if the particle shape remains the same in all compared samples.[9,18,19] A systematic change of K with sample composition may reveal a trend in particle polydispersity. If $\langle R_g^2\rangle$ was known, K could be calculated from the product $K\cdot\langle R_g^2\rangle$, which is obtained by the analysis of $D_{app} = f(q^2)$ according to **eq (SI11)**. Unfortunately, $\langle R_g^2\rangle$ is unknown. However, $\langle R_g^2\rangle$ could be obtained from the slope of a Zimm plot and only differs from $[\xi(\mathrm{SLS})]^2$ by a constant. Therefore, the use of $K\langle R_g^2\rangle\cdot\xi(\mathrm{SLS})^{-2}$ as a measure for sample polydispersity was considered reasonable and is displayed in **Table S6**. As all BlueH/$C_{12}E_5$ co-assemblies are expected to

possess a cylindrical shape and show fluctuation scattering, values of $K\langle R_g^2\rangle \cdot \xi(\text{SLS})^{-2}$ can be compared. Accordingly, **Table S6** suggests, that the polydispersity of BlueH/$C_{12}E_5$ co-assemblies increases with increasing concentration of BlueH. This is in accord with the increasing width of the decay time τ from $g^{(2)}(\tau)$ with increasing [BlueH] (**Figure S16**).

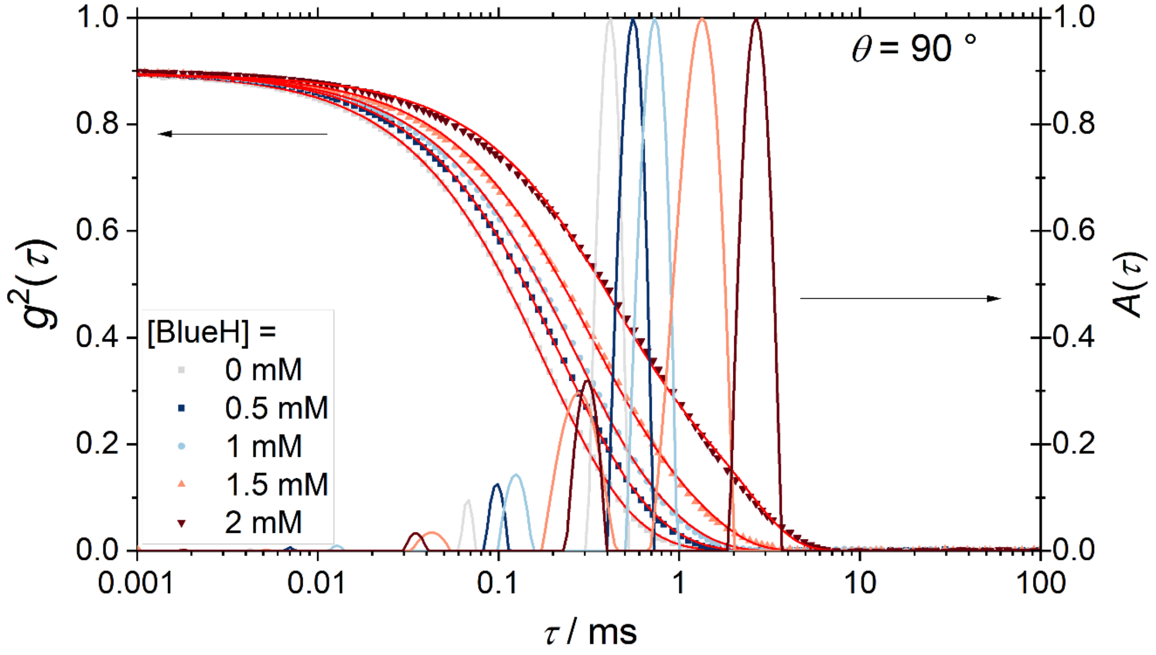

Figure S16: Intensity autocorrelation functions $g^{(2)}(\tau)$ of samples containing $hC_{12}hE_5$ at a concentration of 25 mM and variable concentrations of BlueH. An isotonic NaCl solution prepared in D_2O served as the solvent. The pD of each solution was pD = 2 and measurements were performed at a sample temperature of 10 °C. The red lines show fits to $g^{(2)}(\tau)$ according to the method of cumulants (eq (SI9)). The thicker, solid lines display the distribution of decay times $A(\tau)$ obtained from CONTIN analysis of respective field autocorrelation functions $g^{(1)}(\tau)$. The line colour corresponds to the symbol colour of the respective concentration of BlueH. Data refer to the scattering angle θ = 90 .

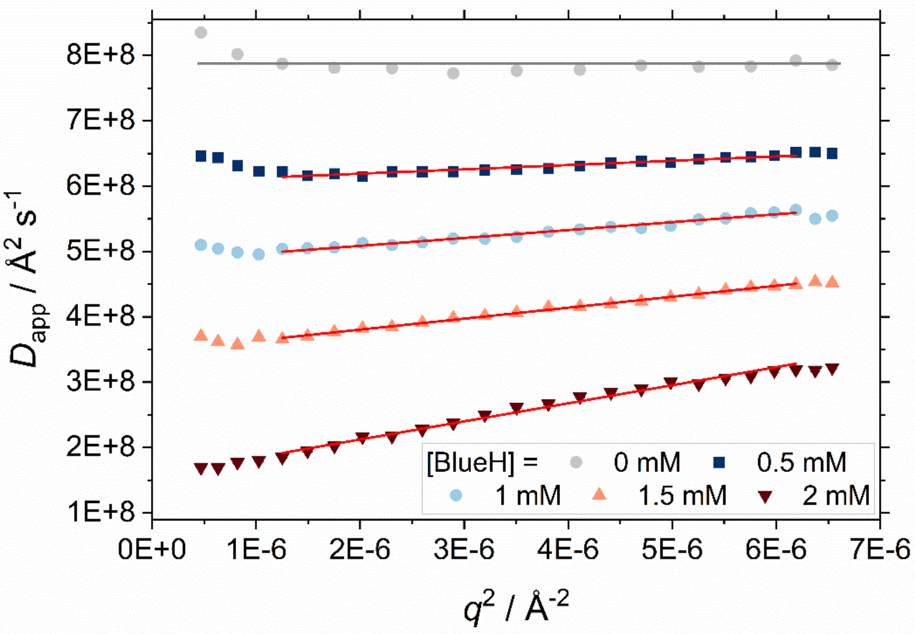

Figure S17: Apparent diffusion coefficients obtained from cumulant analysis of DLS intensity correlation functions from samples containing $hC_{12}hE_5$ at a concentration of 25 mM and varying concentrations of BlueH. An isotonic NaCl solution in D_2O served as the solvent. Samples had a pD of 2 and were measured at a sample temperature of 10 °C. Red lines denote data evaluation according to **eq (SI7)**. In the case of [BlueH] = 0 mM, the grey line indicates the average D_{app}.

To conclude this section, it was found that correlation lengths ξ for concentration fluctuations of the number density of BlueH/$C_{12}E_5$ co-assemblies can be obtained from Ornstein-Zernike plots of SLS data. They compare well to ξ obtained from SANS as long as the corresponding Guinier plateau is reached in the SANS curve. DLS data do not permit to observe the presence of two or more different types of modes, but point towards a broad size distribution of BlueH/$C_{12}E_5$ co-assemblies. The width of this distribution increases with increasing concentration of BlueH.

SI8. Determination of the SANS match point of $C_{12}E_5$ in D_2O

Solutions containing $mC_{12}mE_5$ were prepared by mixing stock solutions of $mC_{12}hE_5$ and $mC_{12}dE_5$ at volume fractions Φ of $mC_{12}hE_5$ and $mC_{12}dE_5$ with respect to the total $C_{12}E_5$ concentration:

$$\Phi(mC_{12}hE_5) = V(mC_{12}hE_5) / [V(mC_{12}hE_5) + V(mC_{12}dE_5)] \quad \text{(SI14)}$$

The total $C_{12}E_5$ concentration in all cases was 25 mM. The solvent was an isotonic NaCl solution with [NaCl] = 154 mM in D_2O at pD = 2. Measurement temperature for SANS was 10 °C. **Figure S18** displays the resulting SANS data as a plot of the square root of the scattering intensity versus $\Phi(mC_{12}hE_5)$ at sixteen different values of the scattering vector q. For each q, a linear fit to $\sqrt{I_q}$ and $-\sqrt{I_q} = f\,[\Phi(mC_{12}hE_5)]$ was performed. Linear fits are shown as lines in **Figure S18**. The value of $\Phi(mC_{12}hE_5)$, at which $\sqrt{I_q} = 0$ and the linear fits intersect, is identified as the match point. It corresponds to a volume ratio of $\Phi(mC_{12}hE_5):\Phi(mC_{12}dE_5)$ = 18.1:81.9. This mixture is denoted as $mC_{12}mE_5$ throughout the entire study.

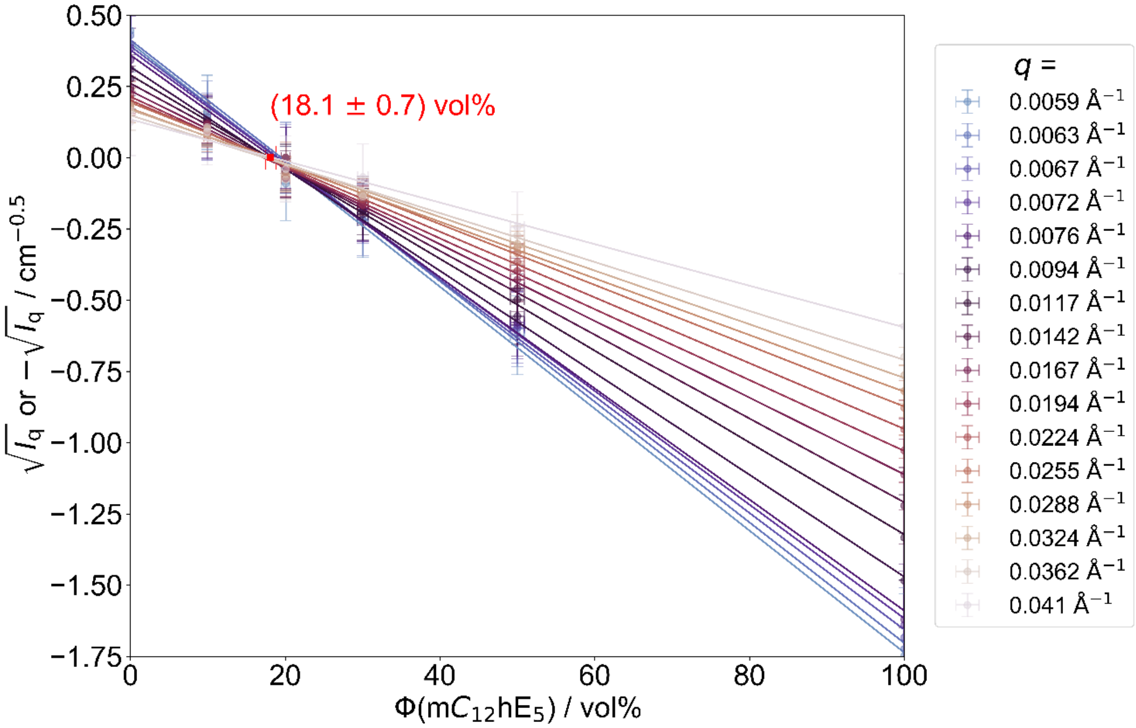

Figure S18: Square root of the scattering intensity observed at the indicated value of the scattering vector q as a function of the volume fraction of $mC_{12}hE_5$ in this mixture containing a total $C_{12}E_5$ concentration of 25 mM. Different values of $[\Phi(mC_{12}hE_5)]$ are achieved by mixing 25 mM solutions of $mC_{12}hE_5$ and $mC_{12}dE_5$ at varying volume ratio. The volume fraction of $mC_{12}hE_5$, at which all linear fits to $\sqrt{I_q}$ and $-\sqrt{I_q} = f\,[\Phi(mC_{12}hE_5)]$ cross through $\sqrt{I_q} = 0$ was identified as the match point.

The success of contrast matching is demonstrated in **Figure S19**, where the empty-cell subtracted SANS curve of a sample containing $mC_{12}mE_5$ at a concentration of 25 mM is shown to be identical to solvent scattering and does not show form factor oscillations.

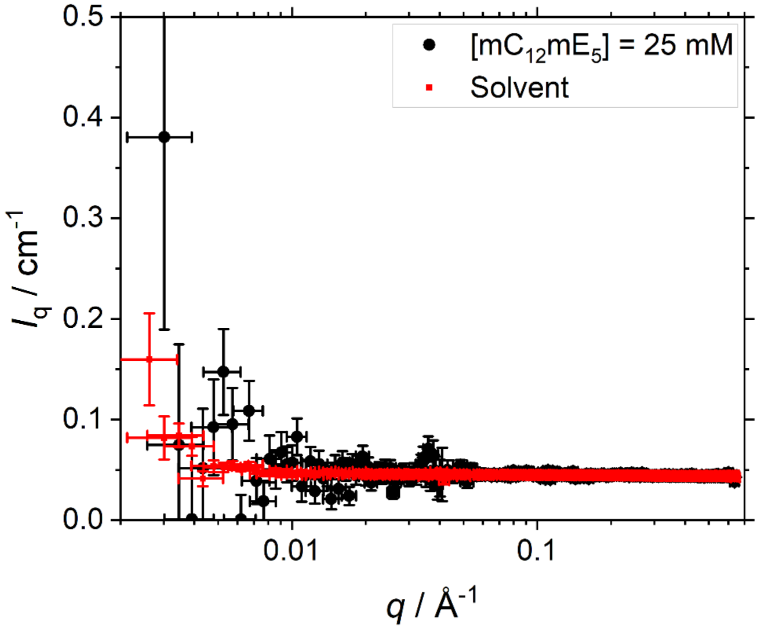

Figure S19: Empty cell subtracted SANS curves of the solvent (isotonic NaCl with [NaCl] = 154 mM in D_2O at pD = 2) and of a solution containing $mC_{12}mE_5$ at a concentration of 25 mM in the same solvent. SANS curves were recorded at a sample temperature of 10 °C. Differences in the signal-to-noise ratio are attributed to differences in measurement times (solvent: 140 min at 17.6 m and 60 min at 4 m, $mC_{12}mE_5$ solution: 20 min at 17.6 m and 10 min at 4 m with 17.6 m and 4 m being the sample-to-rear-detector distances).

SI9. NMR-spectra

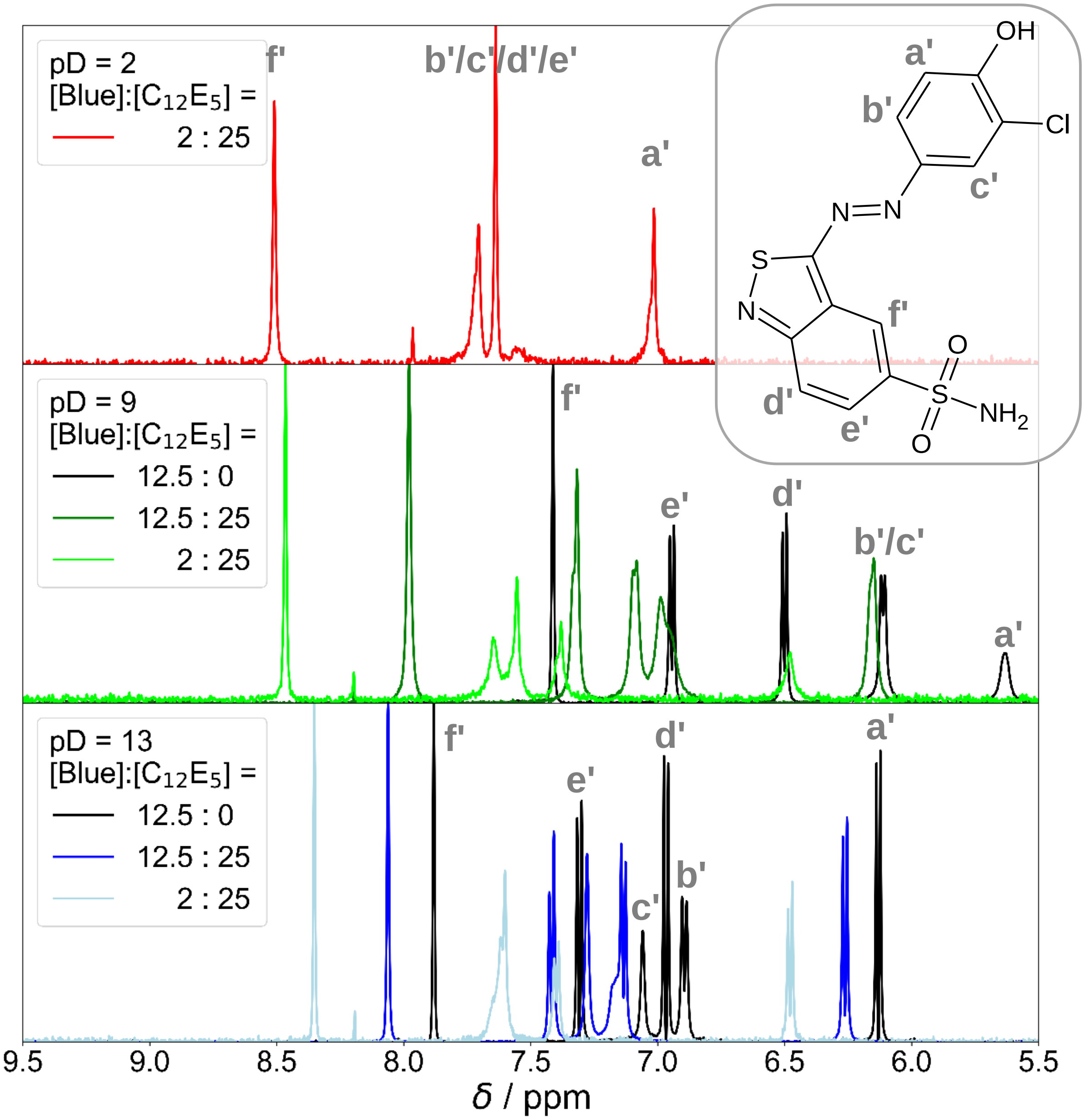

Figure S20: Blue resonance region in ^{1}H-NMR spectra of solutions containing Blue without surfactant or with $hC_{12}hE_5$ at a concentration of [$hC_{12}hE_5$] = 25 mM as indicated in the inset. NMR-spectra were recorded at different sample pDs, indicated in the inset of the respective plot. The spectra of pure Blue solutions without surfactant at pD = 9 and pD = 13 are shown as black lines together with the peak assignment. Due to its low water solubility, the ^{1}H-NMR spectrum of a pure BlueH solution (pD = 2) could not be recorded. Solutions were prepared in an isotonic NaCl solution (I = 0.154 M) in D_2O and spectra were recorded at a sample temperature of 10 °C.

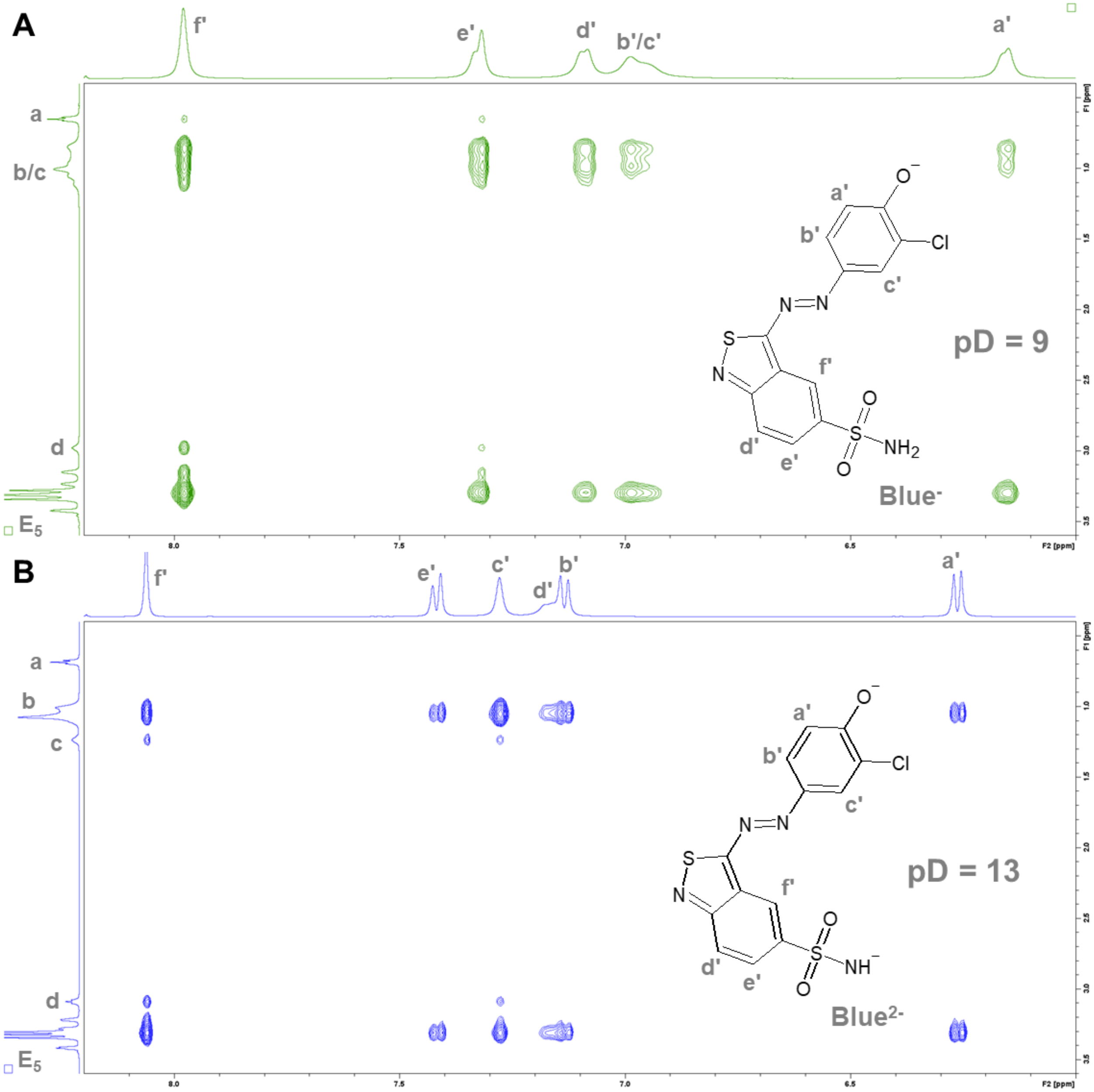

Figure S21: Section of NOESY spectra recorded from solutions containing Blue at a concentration of [Blue] = 12.5 mM and $hC_{12}hE_5$ at a concentration of [$hC_{12}hE_5$] = 25 mM. An isotonic NaCl solution (I = 0.154 M) in D_2O served as the solvent. All measurements were performed at 10 °C.

SI10. Comparison of parameters from full contrast and $C_{12}E_5$ contrast matched SANS

Table S7, **Table S8** and **Table S9** display results from form factor fitting of SANS curves shown in the main document. The first column denotes the figure in the main document, where the corresponding fit is displayed. The form factor models and their combination with a structure factor are indicated in the tables. Cross-section information from the best fit to full contrast data in the high-q region ($q > 0.045$ Å^{-1}) are compared with the results from fits to $C_{12}E_5$-matched SANS curves. For fits to $C_{12}E_5$-matched SANS-curves, n_p corresponds to the number density of respective particles and f_c is a correction factor accounting for deviations between the assumed and the actual contrast. The contrast is given by the scattering length density difference $\Delta\eta$ between the particle and the solvent. It was set to $\Delta\eta = 3.358 \cdot 10^{-6}$ Å^{-2}. If a core-shell distribution of scattering length densities was assumed for fits to $C_{12}E_5$-matched SANS curves, the contrast between solvent and shell was estimated to $\Delta\eta = 3.358 \cdot 10^{-6}$ Å^{-2} and the contrast between the solvent and the core was fixed to $\Delta\eta = 0$. No size distribution was assumed for any dimension. For fits to full contrast data at $q > 0.045$ Å^{-1}, a log-normal distribution of the core radius was assumed, resulting in the mean core radius r_{core} and with a standard deviation SD calculated from the distribution. Ornstein-Zernike parameters obtained from fitting full contrast SANS curves with the model which resulted in the best fit according to Table 2 of the main document are included for comparison. If no error is displayed for a parameter, the value was fixed during fitting.

Figure S22, **Figure S23** and **Figure S24** display full contrast and $C_{12}E_5$-matched SANS curves for pD = 2, pD = 9 and pD = 13 respectively. The left image displays full contrast curves with the best fit, whereas the right image displays corresponding $C_{12}E_5$-matched SANS curves with the respective best fit. All these SANS curves are shown elsewhere and are re-displayed here again to permit a direct comparison between the full contrast and the $C_{12}E_5$-matched case.

Table S7: Results from form factor fitting of SANS curves at pD = 2 shown in **Figure 8** of the main document. The first column refers to the respective plot as it is displayed in **Figure 8**. In the case of $C_{12}E_5$-matched SANS-curves the length *L* inevitably corresponds to the core length of the cylinders. Distribution of Blue along the core may not be homogeneous thereby affecting the extracted length *L*. Data from fits to full contrast experiments refer to the best fit with the model indicated in the respective header of the column. The first column of the full contrast experiments refers to fits to the high-*q* range (q > 0.045 $Å^{-1}$), whereas the second column refers to fits to the entire *q*-range according to the two-step fit strategy.

<table>
<tr><td colspan="13">pD = 2</td></tr>
<tr><td></td><td colspan="6">$C_{12}E_5$-matched [$mC_{12}mE_5$] = 25 mM</td><td colspan="6">Full contrast [$hC_{12}hE_5$] = 25 mM</td></tr>
<tr><td>Model: $I(q)$ =</td><td colspan="6">$n_p f_c \cdot P_{cylinder}(q) \cdot C(q)$</td><td colspan="3">$n_p \cdot P_{core\text{-}linshell\text{-}cylinder}(q)$
for $q > 0.045$ $Å^{-1}$</td><td colspan="3">$n_p \cdot P_{cap\text{-}core\text{-}shell\text{-}cylinder}(q) \cdot C(q)$</td></tr>
<tr><td>[BlueH] = 1 mM</td><td>Length L / Å</td><td>Radius R / Å</td><td>$n_p f_c$ / 10^{16} cm^{-3}</td><td>κ</td><td>ξ / Å</td><td>χ_{red}^2</td><td>r_{core} / Å</td><td>$SD(r_{core})$ / Å</td><td>r_{shell} / Å</td><td>L_{core} / Å</td><td>κ</td><td>ξ / Å</td></tr>
<tr><td>dotted line</td><td>31.9 ± 0.8</td><td>3.0 ± 0.2</td><td>163 ± 22</td><td>17.1 (SANS)</td><td>201 (SANS)</td><td>3.5185</td><td rowspan="3">12.7 ± 0.3</td><td rowspan="3">3.6 ± 0.2</td><td rowspan="3">14.4 ± 0.3</td><td rowspan="3">66</td><td rowspan="3">17.1 ± 0.1</td><td rowspan="3">201 ± 0.1</td></tr>
<tr><td>dashed line</td><td>10.0 ± 34</td><td>5.7 ± 10</td><td>88 ± 6</td><td>41 ± 2</td><td>201 (SANS)</td><td>2.1851</td></tr>
<tr><td>solid line</td><td>10.1 ± 300</td><td>5.8 ± 9</td><td>87 ± 5</td><td>48 ± 2</td><td>221 (SLS)</td><td>2.1513</td></tr>
<tr><td>[BlueH] = 2 mM</td><td>Length L / Å</td><td>Radius R / Å</td><td>$n_p f_c$ / 10^{16} cm^{-3}</td><td>κ</td><td>ξ / Å</td><td>χ_{red}^2</td><td>r_{core} / Å</td><td>$SD(r_{core})$ / Å</td><td>r_{shell} / Å</td><td>L_{core} / Å</td><td>κ</td><td>ξ / Å</td></tr>
<tr><td>dotted line</td><td>66 (SANS)</td><td>3.7 ± 0.2</td><td>26 ± 4</td><td>43.6 (SANS)</td><td>322 (SANS)</td><td>1.7020</td><td rowspan="3">13.3 ± 0.3</td><td rowspan="3">3.7 ± 0.2</td><td rowspan="3">13.6 ± 0.3</td><td rowspan="3">66</td><td rowspan="3">43.6 ± 0.2</td><td rowspan="3">322 ± 1</td></tr>
<tr><td>dashed line</td><td>66 (SANS)</td><td>2.9 ± 0.2</td><td>56 ± 11</td><td>73 ± 3</td><td>322 (SANS)</td><td>1.6842</td></tr>
<tr><td>solid line</td><td>66 (SANS)</td><td>3.0 ± 0.2</td><td>53 ± 10</td><td>156 ± 4</td><td>483 (SLS)</td><td>1.5890</td></tr>
</table>

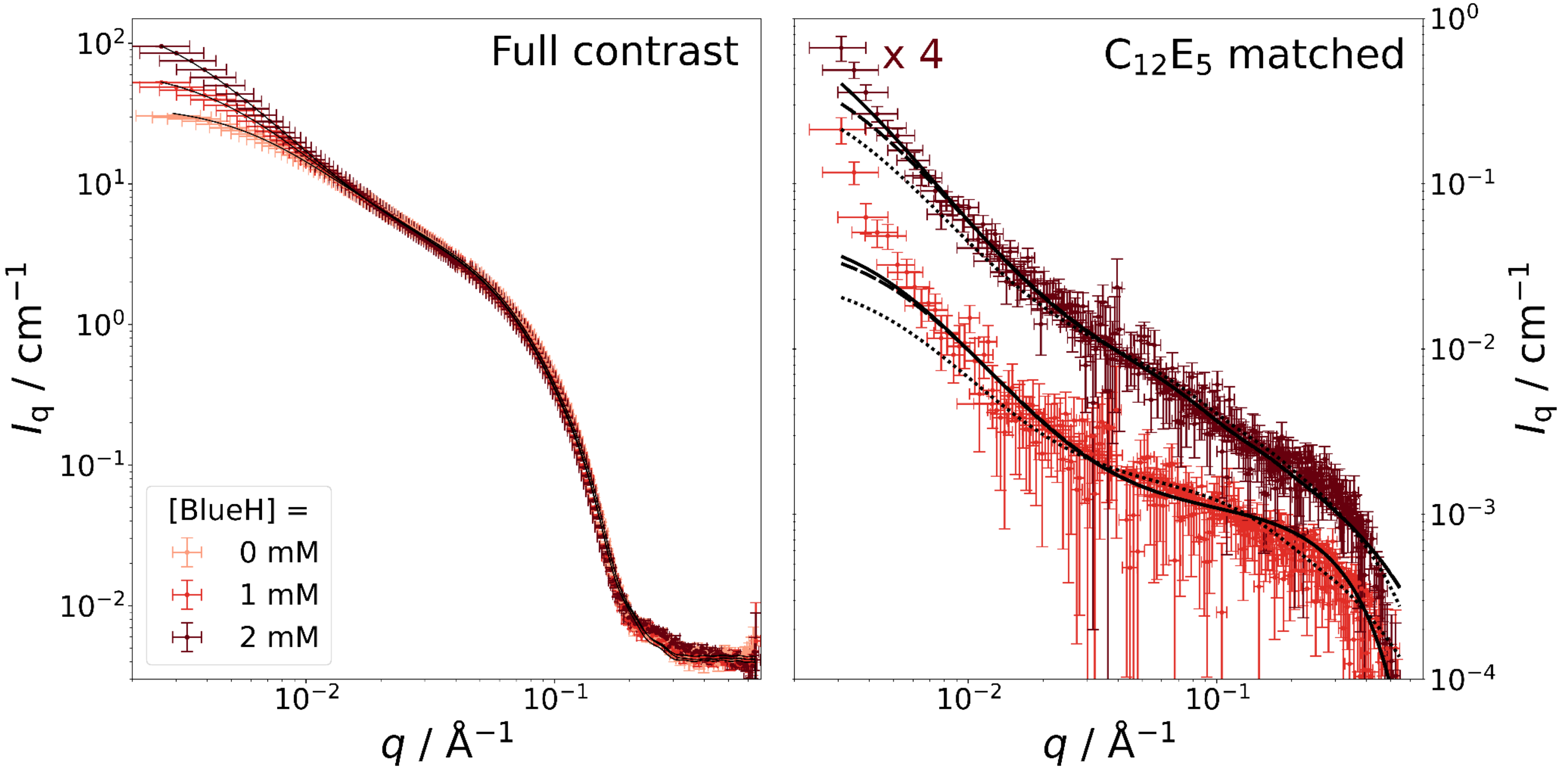

Figure S22: Left: Full contrast SANS curves from solutions containing [$hC_{12}hE_5$] = 25 mM and Blue at different concentrations at pD = 2. Black, solid lines display the best fit to the entire q-range. The used model is indicated in **Table 2** in the main document and in **Table S7**. Right: $C_{12}E_5$-matched SANS curves from solutions containing [$mC_{12}mE_5$] = 25 mM and Blue at different concentrations at pD = 2. Dotted line (**.....**): Fit with the form factor of a cylinder including Ornstein-Zernike scattering as a structure factor with κ and ξ obtained from the corresponding SANS full contrast measurement. Dashed line (**---**): Fit with the same model, but only ξ was kept constant according to the value obtained from the SANS full contrast measurement. Solid line (**—**): Fit with the same model, but ξ was kept constant according to a value obtained from static light scattering. The used model and parameters obtained from the fit are given in **Table S7**. In all cases, an isotonic NaCl solution, which was prepared in D_2O, served as the solvent and measurements were performed at a sample temperature of 10 °C.

Table S8: Results from form factor fitting of SANS curves at pD = 9 shown in **Figure 9** of the main document. The first column refers to the respective plot as it is displayed in **Figure 9**. Data from fits to full contrast experiments refer to the best fit with the model indicated in the respective header of the column. The first column of the full contrast experiments refers to fits to the high-q range (q > 0.045 Å^{-1}), whereas the second column refers to fits to the entire q-range according to the two-step fit strategy.

**pD = 9**												
	$C_{12}E_5$-matched [$mC_{12}mE_5$] = 25 mM						Full contrast [$hC_{12}hE_5$] = 25 mM					
**Model:** $I(q)$ =	$I_{q,Guinier}(q) + n_p f_c \cdot P_{core\text{-}shell\text{-}cylinder}(q) \cdot C(q)$						$n_p \cdot P_{core\text{-}linshell\text{-}cylinder}(q)$ for q > 0.045 Å^{-1}			$n_p \cdot P_{cap\text{-}core\text{-}shell\text{-}cylinder}(q) \cdot C(q)$		
[Blue$^-$] = 1 mM	Length L / Å	r_{core} / Å	r_{shell} / Å	κ	ξ / Å	χ_{red}^2	r_{core} / Å	$SD(r_{core})$ / Å	r_{shell} / Å	L_{core} / Å	κ	ξ / Å
solid line	66 (SANS)	7.4	21 ± 4	11.1 (SANS)	164 (SANS)	0.9000	12.5 ± 0.3	3.5 ± 0.2	11.2 ± 0.2	66	11.1 ± 0.1	164 ± 1
	$n_p f_c$ / 10^{16} cm^{-3}: 0.0014 ± 0.0008											
	R_g from $I_{q,Guinier}(q)$ / Å: 6.0 ± 0.2											
[Blue$^-$] = 2 mM	Length L / Å	r_{core} / Å	r_{shell} / Å	κ	ξ / Å	χ_{red}^2	r_{core} / Å	$SD(r_{core})$ / Å	r_{shell} / Å	L_{core} / Å	κ	ξ / Å
solid line	66 (SANS)	7.4	19 ± 2	12 (SANS)	174 (SANS)	1.4912	12.4 ± 0.3	3.5 ± 0.2	14.5 ± 0.3	66	12.0 ± 0.1	174 ± 1
	$n_p f_c$ / 10^{16} cm^{-3}: 0.006 ± 0.002											
	R_g from $I_{q,Guinier}(q)$ / Å: 4.94 ± 0.06											
[Blue$^-$] = 6.25 mM	Length L / Å	r_{core} / Å	r_{shell} / Å	κ	ξ / Å	χ_{red}^2	r_{core} / Å	$SD(r_{core})$ / Å	r_{shell} / Å	L_{core} / Å	κ	ξ / Å
solid line	40 (SANS)	7.9 ± 0.3	17.6 ± 0.2	7.1 (SANS)	97 (SANS)	3.2898	12.3 ± 0.3	3.4 ± 0.2	13.2 ± 0.3	40	7.1 ± 0.1	97 ± 1
	$n_p f_c$ / 10^{16} cm^{-3}: 0.0955 ± 0.0004											
	R_g from $I_{q,Guinier}(q)$ / Å: 4.24 ± 0.07											
[Blue$^-$] = 12.5 mM	Length L / Å	r_{core} / Å	r_{shell} / Å	κ	ξ / Å	χ_{red}^2	r_{core} / Å	$SD(r_{core})$ / Å	r_{shell} / Å	L_{core} / Å	κ	ξ / Å
solid line	40 (SANS)	6.9 ± 0.4	16.2 ± 0.3	2.7 (SANS)	52 (SANS)	2.9459	13.3 ± 0.2	2.91 ± 0.09	10.8 ± 0.3	40	2.7 ± 0.1	52 ± 1
	$n_p f_c$ / 10^{16} cm^{-3}: 0.191 ± 0.0007											
	R_g from $I_{q,Guinier}(q)$ / Å: 6.5 ± 0.2											

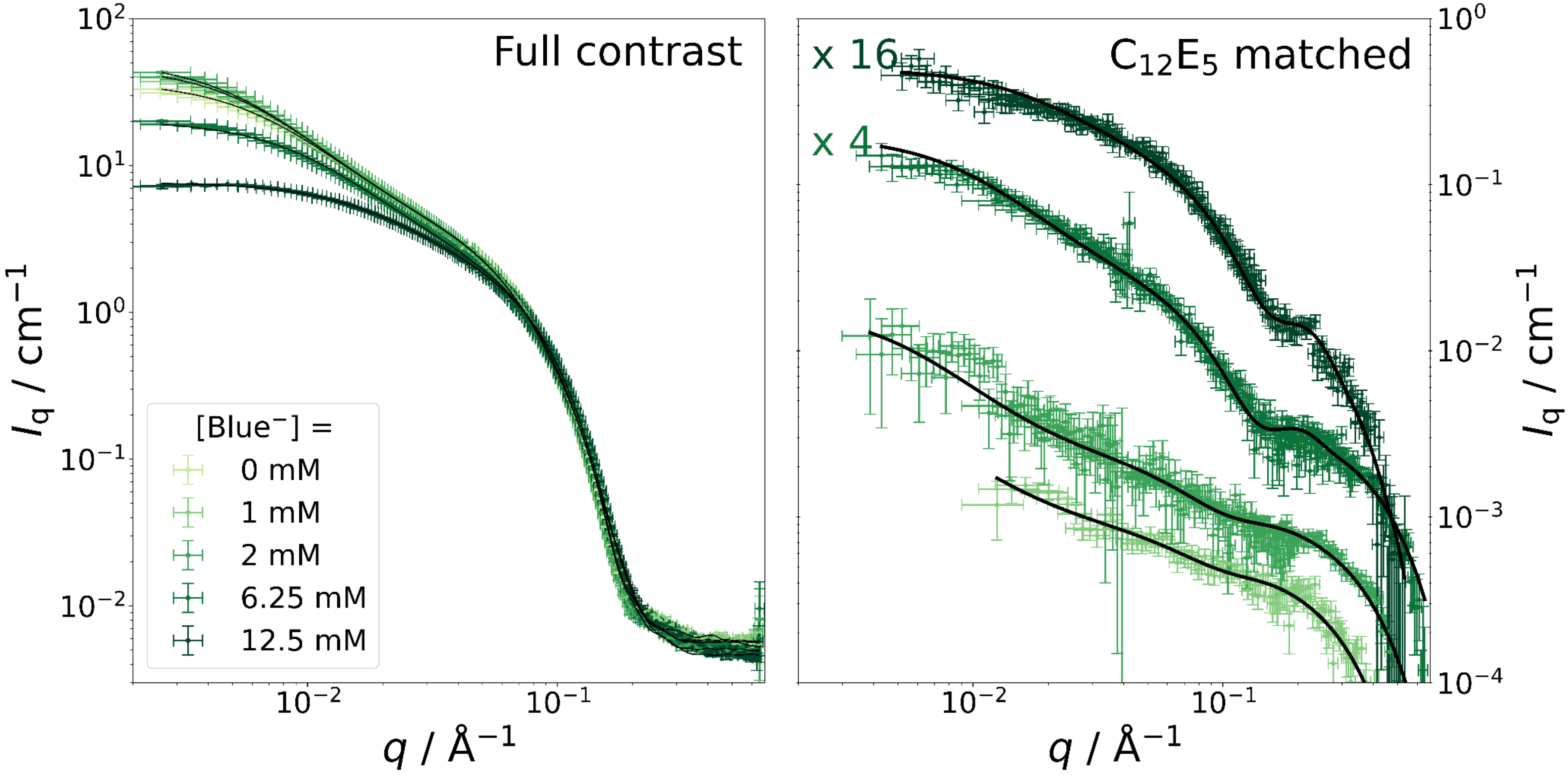

Figure S23: Left: Full contrast SANS curves from solutions containing [$hC_{12}hE_5$] = 25 mM and Blue at different concentrations at pD = 9. Black, solid lines display the best fit to the entire q-range. The used model is indicated in **Table 2** in the main document and in **Table S8**. Right: $C_{12}E_5$-matched SANS curves from solutions containing [$mC_{12}mE_5$] = 25 mM and Blue at different concentrations at pD = 9. Black, solid lines display the best fit to $C_{12}E_5$-matched SANS curves. The used model and parameters obtained from the fit are given in **Table S8**. In all cases, an isotonic NaCl solution, which was prepared in D_2O, served as the solvent and measurements were performed at a sample temperature of 10 °C.

Table S9: Results from form factor fitting of SANS curves at pD = 13 shown in **Figure 10** of the main document. The first column refers to the respective plot as it is displayed in **Figure 10**. Data from fits to full contrast experiments refer to the best fit with the model indicated in the respective header of the column. The first column of the full contrast experiments refers to fits to the high-q range (q > 0.045 Å^{-1}), whereas the second column refers to fits to the entire q-range according to the two-step fit strategy.

**pD = 13**												
	$C_{12}E_5$-matched [$mC_{12}mE_5$] = 25 mM						Full contrast [$hC_{12}hE_5$] = 25 mM					
**Model:** $I(q)$ =	$I_{q,Guinier}(q) + n_p f_c \cdot P_{core-shell-cylinder}(q) \cdot C(q)$						$n_p \cdot P_{core-linshell-cylinder}(q)$ for q > 0.045 Å^{-1}			$n_p \cdot P_{cap-core-shell-cylinder}(q) \cdot C(q)$		
[Blue^{2-}] = 1 mM	Length L / Å	r_{core} / Å	r_{shell} / Å	κ	ξ / Å	χ_{red}^2	r_{core} / Å	$SD(r_{core})$ / Å	r_{shell} / Å	L_{core} / Å	κ	ξ / Å
solid line	40 (SANS)	13 ± 2	4.79 ± 1.7	5.9 (SANS)	87 (SANS)	1.3152	13.7 ± 0.2	3.3 ± 0.1	12.0 ± 0.3	40	5.9 ± 0.1	87 ± 1
	$n_p f_c$ / 10^{16} cm^{-3}: 0.08 ± 0.02<br>R_g from $I_{q,Guinier}(q)$ / Å: 5.39											
[Blue^{2-}] = 2 mM	Length L / Å	r_{core} / Å	r_{shell} / Å	κ	ξ / Å	χ_{red}^2	r_{core} / Å	$SD(r_{core})$ / Å	r_{shell} / Å	L_{core} / Å	κ	ξ / Å
solid line	40 (SANS)	13.6 ± 0.7	4.79 ± 1.7	1.8 (SANS)	47 (SANS)	1.1606	14.5 ± 0.3	2.8 ± 0.2	10.1 ± 0.3	40	1.8 ± 0.1	47 ± 1
	$n_p f_c$ / 10^{16} cm^{-3}: 0.16 ± 0.02<br>R_g from $I_{q,Guinier}(q)$ / Å: 5.39											
**Model:** $I(q)$ =	$I_{q,Guinier}(q) + n_p f_c \cdot P_{core-shell-sphere}(q)$						$n_p \cdot P_{core-linshell-sphere}(q)$ for q > 0.045 Å^{-1}			$n_p \cdot P_{core-shell-sphere}(q) \cdot C(q)$		
[Blue^{2-}] = 6.25 mM	R_g	r_{core} / Å	r_{shell} / Å	$n_p f_c$ / 10^{16} cm^{-3}	χ_{red}^2		r_{core} / Å	$SD(r_{core})$ / Å	r_{shell} / Å	κ		ξ / Å
dashed line	5.39	21.1 ± 0.4	4.79 ± 1.7	0.13 ± 0.008	3.6223		16.9 ± 0.2	3.72 ± 0.07	11.7 ± 0.2	-		-
[Blue^{2-}] = 12.5 mM	R_g	r_{core} / Å	r_{shell} / Å	$n_p f_c$ / 10^{16} cm^{-3}	χ_{red}^2		r_{core} / Å	$SD(r_{core})$ / Å	r_{shell} / Å	κ		ξ / Å
dashed line	5.39	17.2 ± 0.3	4.79 ± 1.7	0.34 ± 0.02	1.8240		16.5 ± 0.2	3.55 ± 0.07	11.7 ± 0.2	-		-

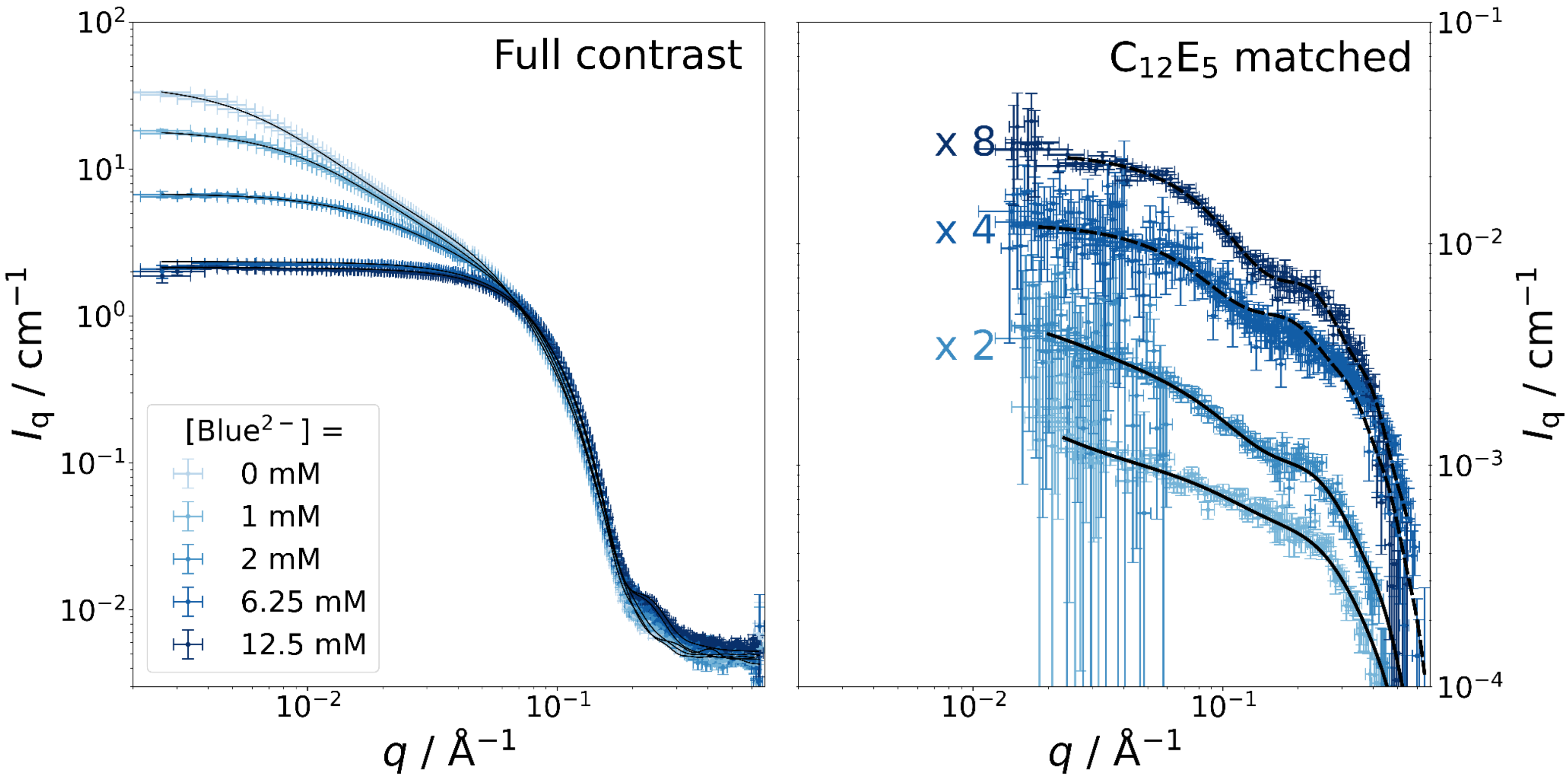

Figure S24: Left: Full contrast SANS curves from solutions containing [$hC_{12}hE_5$] = 25 mM and Blue at different concentrations at pD = 13. Black, solid lines display the best fit to the entire q-range. The used model is indicated in **Table 2** in the main document and in **Table S9**. Right: $C_{12}E_5$-matched SANS curves from solutions containing [$mC_{12}mE_5$] = 25 mM and Blue at different concentrations at pD = 13. Black, solid lines and black, dashed lines display the best fit to $C_{12}E_5$-matched SANS curves. The used model and parameters obtained from the fit are given in **Table S9**. In all cases, an isotonic NaCl solution, which was prepared in D_2O, served as the solvent and measurements were performed at a sample temperature of 10 °C.

SI11. Synthesis of deuterated $C_{12}E_5$

General. Reagents were purchased from commercial suppliers and used without further purification. Triethylene glycol-d_{12},[26] 2-(2-bromoethoxy-$d_{4(100\%)}$)tetrahydro-2H-pyran,[26] and 1-bromododecane-d_{25}[27] were synthesized according to literature procedures. Column chromatography was carried out using flash silica gel (40−63 μm, 230−400 mesh). ^{1}H and ^{13}C NMR were recorded on a Bruker AVANCE spectrometer at 500 and 125 MHz, respectively, unless specified otherwise. High-resolution mass spectra were obtained on a Waters XEVO-G2 QTOF mass spectrometer.

Pentaethylene glycol-d_{20}. To a suspension of sodium hydride (60wt% in mineral oil, 1.5 g, 37 mmol) in anhydrous DMF (55mL) and anhydrous THF (55 mL) was added triethylene glycol-$d_{12(100\%)}$ (1.5 g, 9.2 mmol) at r.t. under N_2. The reaction mixture was allowed to stir at 65°C for 30 min in prior to addition of 2-(2-bromoethoxy-$d_{4(100\%)}$)tetrahydro-2H-pyran (4.1g, 19.3 mmol) at r.t. After stirring at r.t for 20 h, the reaction mixture was quenched with sat. NH_4Cl solution (100 mL) at 0°C and extracted with DCM (100 mL x 3). The organic phase was washed with brine (100 mL), dried over $MgSO_4$, filtered and evaporated under reduced pressure. The residue was purified by column chromatography using hexane/EtOAc (5:95 → 8:2 →2:8) as eluent to afford a colourless oil (3.1g, 7.3 mmol), which was re-dissolved in MeOH (30 mL) and THF (30 mL) in prior to addition of pyridinium *p*-toluenesulfonate (182 mg, 0.7 mmol). After stirring at 45°C for 18h, the reaction mixture was evaporated under reduced pressure. The residue was purified by chromatography using hexane/EtOAc/MeOH (50:50:0 →20:80:0→0:100:0 →0:50:50) as eluent to afford the title compound as a colourless oil (1.7 g, 72%). ^{1}H NMR data was not collected since the title compound does not contain any H-atoms. The product was identified by co-spotting with pentaethylene glycol on TLC. HRMS (ESI+): Calc'd for $C_{10}H_2D_{20}O_6$ $[M+Na]^+$: 281.2569, Found: 281.2569.

Pentaethylene glycol monododecyl ether-$d_{25(90\%)}$ ($mC_{12}hE_5$). To a solution of pentaethylene glycol (2.5g, 10.5 mmol) in anhydrous DMF (10.5 mL) was portionwise added sodium hydride (60wt% in mineral oil, 420 mg, 10.5 mmol) at r.t. under N_2. The reaction mixture was allowed to stir for 30 min in prior to addition of 1-bromododecane-$d_{25(90\%)}$ (0.6 mL, 2.6 mmol) in anhydrous DMF (10.5 mL) at 0 °C. After stirring for 22 h at r.t, the reaction mixture was quenched with sat. NH_4Cl solution at 0°C and extracted with EtOAc (100 mL x 3). The organic phase was washed with brine (100 mL), dried over $MgSO_4$, filtered and evaporated under reduced pressure. The residue was purified by column chromatography using hexane/EtOAc (1:1) as eluent to afford the title compound as an oil (1.0 g, 89%). ^{1}H-NMR (500 MHz; $CDCl_3$): δ 3.71 (t, J = 4.0 Hz, 2H), 3.59

– 3.68 (m, 16H), 3.55 – 3.57 (m, 2H), 2.56 – 2.60 (m, 1.2 H*), 1.18 – 1.24 (m, 1.7 H*), 0.81 – 0.83 (m, 0.3 H*). *Contribution from residual protium. ^{13}C NMR (125 MHz; $CDCl_3$): δ 72.5, 70.6 (3C), 70.5, 70.3, 69.9, 61.7, 28.5, 28.4, 28.3. HRMS (ESI+): Calc'd for $C_{22}H_{21}D_{25}O_6$[M+Na]$^+$: 454.4761, Found: 454.4745.

Pentaethylene glycol -d$_{20(100\%)}$ monododecyl ether-d$_{25(90\%)}$ (mC$_{12}$dE$_5$). To a solution of pentaethylene glycol-d$_{20(100\%)}$ (1.7 g, 6.6 mmol) in anhydrous DMF (7 mL) was portionwise added sodium hydride (60wt% in mineral oil, 264 mg, 6.6 mmol) at r.t. under N_2. The reaction mixture was allowed to stir for 30 min in prior to addition of 1-bromododecane-d$_{25(90\%)}$ (0.7 mL, 3.3 mmol) in anhydrous DMF (7 mL) at 0 °C. After stirring for 22 h at r.t, the reaction mixture was quenched with sat. NH_4Cl solution at 0°C and extracted with EtOAc (100 mL x 3). The organic phase was washed with brine, dried over $MgSO_4$, filtered and evaporated under reduced pressure. The residue was purified by column chromatography using hexane/EtOAc (1:1) as eluent to afford the title compound as an oil (1.2 g, 80%). ^{1}H-NMR (500 MHz; $CDCl_3$): δ 3.84 – 3.87 (m, 0.02H*), 3.49 – 3.63 (m, 0.13H*), 2.59 (s, 1H), 1.18 – 1.26 (m, 1.3 H*), 0.81 – 0.83 (m, 0.2 H) *Contribution from residual protium. ^{13}C NMR (125 MHz; $CDCl_3$): δ 69.8, 69.6, 69.5, 61.1, 60.9, 28.5, 28.2. HRMS (ESI+): Calc'd for $C_{22}HD_{45}O_6$[M+Na]$^+$: 474.6017, Found: 474.6006.

^{1}H-NMR of pentaethylene glycol monododecyl ether -d_{25} (mC_{12}hE_5).

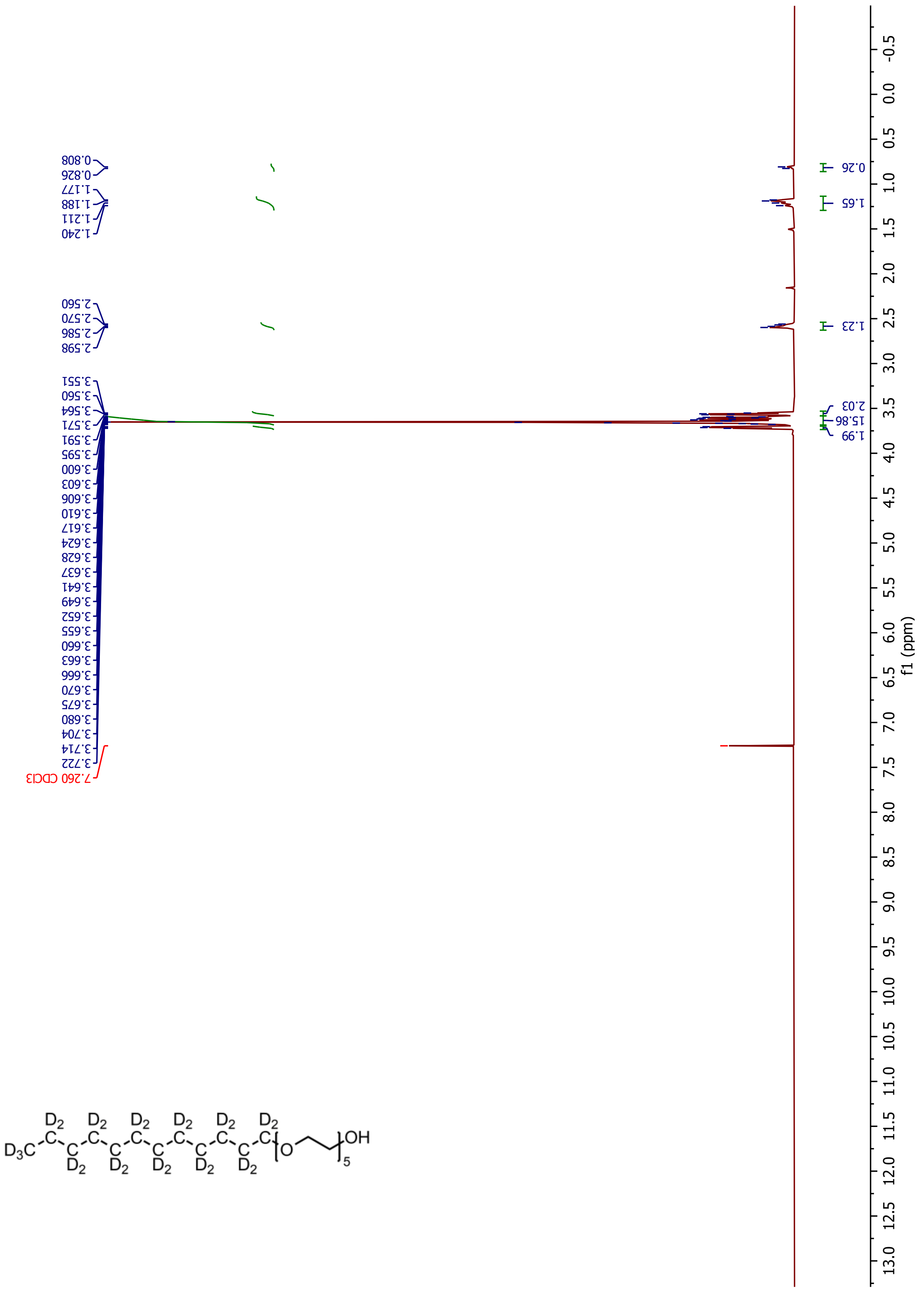

^{13}C-NMR of pentaethylene glycol monododecyl ether -d_{25} ($mC_{12}hE_5$).

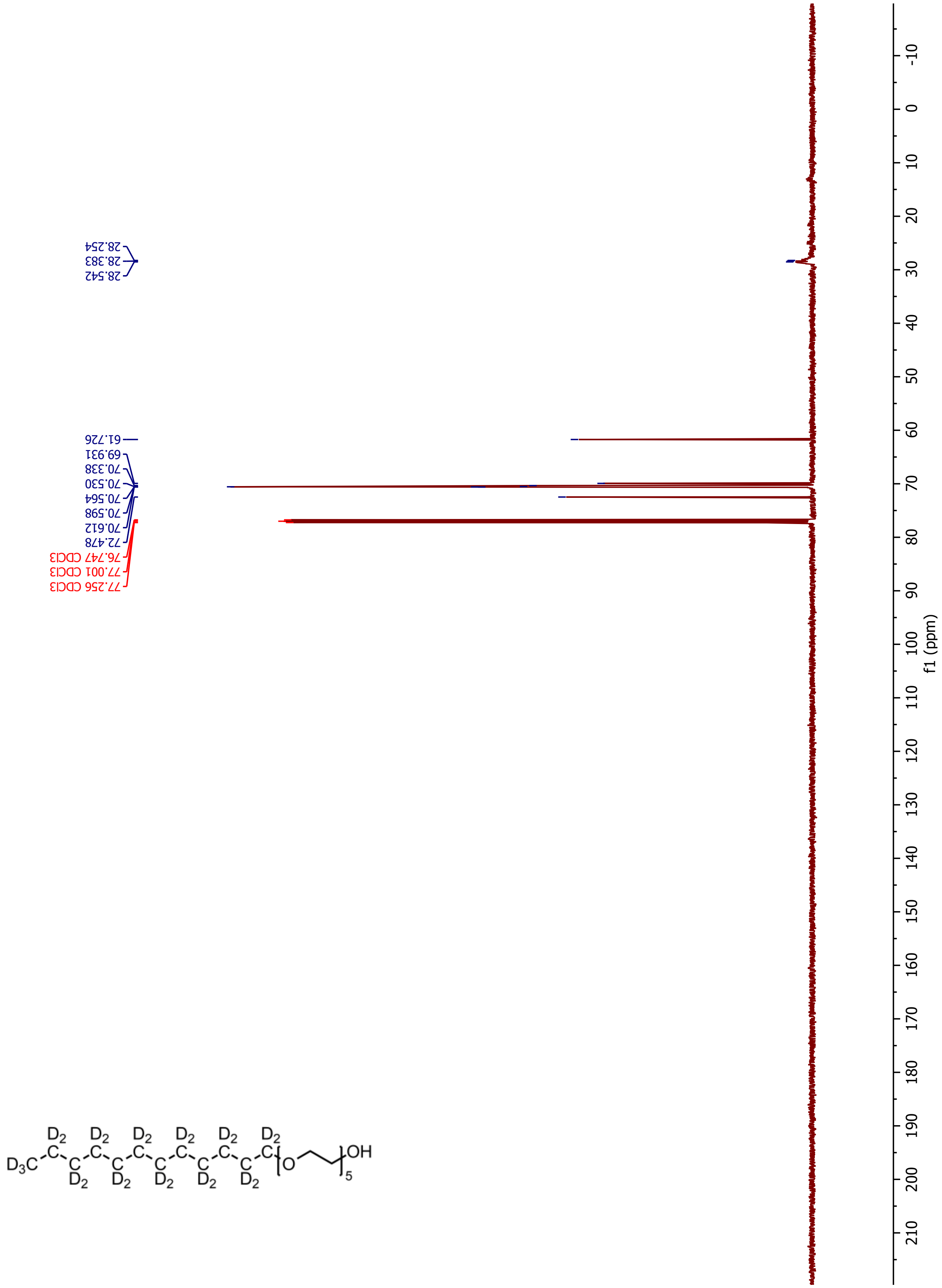

^{1}H-NMR of pentaethylene glycol monododecyl ether-d_{45} (mC_{12}dE_5).

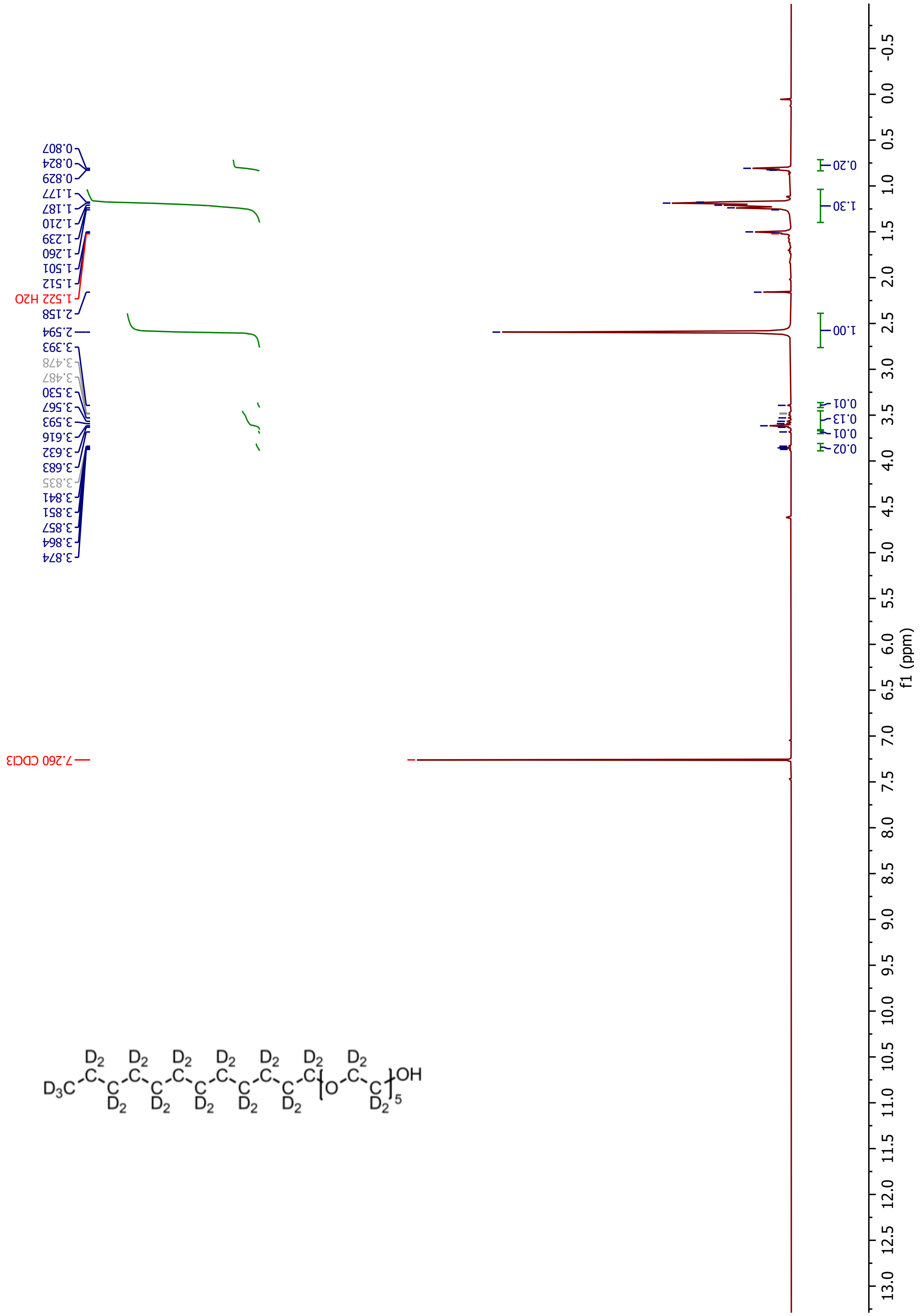

^{13}C-NMR of pentaethylene glycol monododecyl ether -d_{45} ($mC_{12}dE_5$).

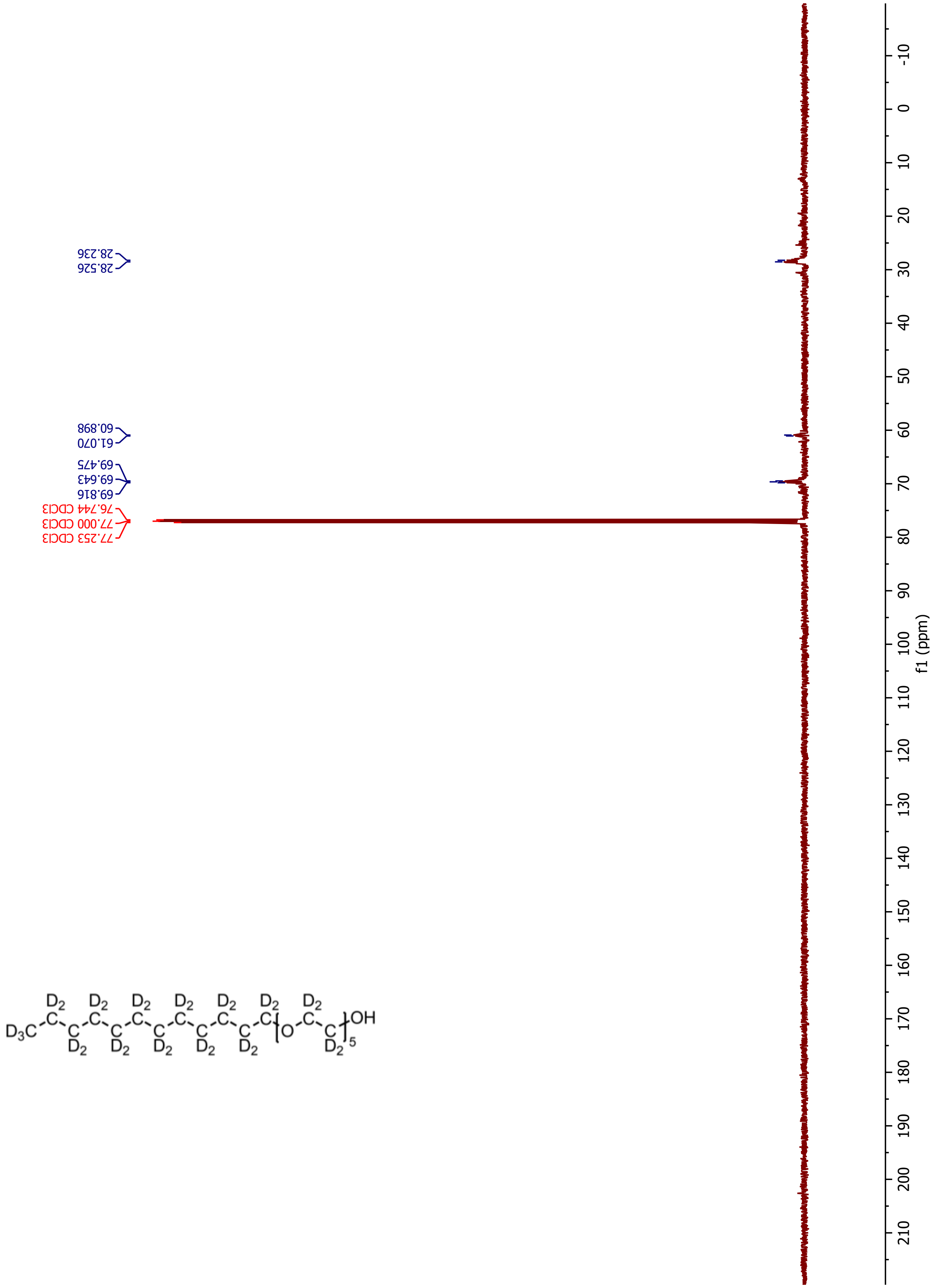

SI12. References

(1) Atkins, P. W.; De Paula, J. *Atkins' Physical Chemistry*, 10th ed.; Oxford University Press: Oxford ; New York, 2014.

(2) Dewhurst, Charles. GRASP, 2023.

(3) *Neutrons, X-Rays, and Light: Scattering Methods Applied to Soft Condensed Matter*, 1st ed.; Lindner, P., Zemb, T., Eds.; North-Holland delta series; Elsevier: Amsterdam ; Boston, 2002.

(4) Breßler, I.; Kohlbrecher, J.; Thünemann, A. F. SASfit: A Tool for Small-Angle Scattering Data Analysis Using a Library of Analytical Expressions. *J Appl Cryst* **2015**, *48* (5), 1587–1598. https://doi.org/10.1107/S1600576715016544.

(5) Kohlbrecher, Joachim. User Guide for the SASfit Software Package, 2023.

(6) Hayter, J. B. Concentrated Colloidal Dispersions Viewed as One-Component Macrofluids. *Faraday Discuss. Chem. Soc.* **1983**, *76* (0), 7–17. https://doi.org/10.1039/DC9837600007.

(7) Glatter, O.; Fritz, G.; Lindner, H.; Brunner-Popela, J.; Mittelbach, R.; Strey, R.; Egelhaaf, S. U. Nonionic Micelles near the Critical Point: Micellar Growth and Attractive Interaction. *Langmuir* **2000**, *16* (23), 8692–8701. https://doi.org/10.1021/la000315s.

(8) Berr, S. S. Solvent Isotope Effects on Alkytrimethylammonium Bromide Micelles as a Function of Alkyl Chain Length. *J. Phys. Chem.* **1987**, *91* (18), 4760–4765. https://doi.org/10.1021/j100302a024.

(9) Schärtl, W. *Light Scattering from Polymer Solutions and Nanoparticle Dispersions*; Springer Laboratory; Springer Berlin Heidelberg: Berlin, Heidelberg, 2007. https://doi.org/10.1007/978-3-540-71951-9.

(10) Corti, M.; Degiorgio, V. Micellar Properties and Critical Fluctuations in Aqueous Solutions of Nonionic Amphiphiles. *J. Phys. Chem.* **1981**, *85* (10), 1442–1445. https://doi.org/10.1021/j150610a033.

(11) Corti, M.; Minero, C.; Degiorgio, V. Cloud Point Transition in Nonionic Micellar Solutions. *J. Phys. Chem.* **1984**, *88* (2), 309–317. https://doi.org/10.1021/j150646a029.

(12) Kita, R.; Kubota, K.; Dobashi, T. Static and Dynamic Light Scattering of a Critical Polydisperse Polymer Solution. *Phys. Rev. E* **1998**, *58* (1), 793–800. https://doi.org/10.1103/PhysRevE.58.793.

(13) Frisken, B. J. Revisiting the Method of Cumulants for the Analysis of Dynamic Light-Scattering Data. *Appl. Opt.* **2001**, *40* (24), 4087–4091. https://doi.org/10.1364/AO.40.004087.

(14) Provencher, S. W. Inverse Problems in Polymer Characterization: Direct Analysis of Polydispersity with Photon Correlation Spectroscopy. *Die Makromolekulare Chemie* **1979**, *180* (1), 201–209. https://doi.org/10.1002/macp.1979.021800119.

(15) Scotti, A.; Liu, W.; Hyatt, J. S.; Herman, E. S.; Choi, H. S.; Kim, J. W.; Lyon, L. A.; Gasser, U.; Fernandez-Nieves, A. The CONTIN Algorithm and Its Application to Determine the Size Distribution of Microgel Suspensions. *J. Chem. Phys.* **2015**, *142* (23), 234905. https://doi.org/10.1063/1.4921686.

(16) Bulut, S.; Hamit, J.; Olsson, U.; Kato, T. On the Concentration-Induced Growth of Nonionic Wormlike Micelles. *Eur. Phys. J. E* **2008**, *27* (3), 261–273. https://doi.org/10.1140/epje/i2008-10379-2.

(17) Cebula, D. J.; Ottewill, R. H. Neutron Scattering Studies on Micelles of Dodecylhexaoxyethylene Glycol Monoether. *Colloid and Polymer Science* **1982**, *260* (12), 1118–1120. https://doi.org/10.1007/BF01411231.

(18) Burchard, W.; Schmidt, M.; Stockmayer, W. H. Influence of Hydrodynamic Preaveraging on Quasi-Elastic Scattering from Flexible Linear and Star-Branched Macromolecules. *Macromolecules* **1980**, *13* (3), 580–587. https://doi.org/10.1021/ma60075a020.

(19) Burchard, W.; Schmidt, M.; Stockmayer, W. H. Information on Polydispersity and Branching from Combined Quasi-Elastic and Intergrated Scattering. *Macromolecules* **1980**, *13* (5), 1265–1272. https://doi.org/10.1021/ma60077a045.

(20) Glatter, O.; Strey, R.; Schubert, K.-V.; Kaler, E. W. III. Experimental Techniques: Small Angle Scattering Applied to Microemulsions. *Berichte der Bunsengesellschaft für physikalische Chemie* **1996**, *100* (3), 323–335. https://doi.org/10.1002/bbpc.19961000319.

(21) Jerke, G.; Pedersen, J. S.; Egelhaaf, S. U.; Schurtenberger, P. Flexibility of Charged and Uncharged Polymer-like Micelles. *Langmuir* **1998**, *14* (21), 6013–6024. https://doi.org/10.1021/la980390r.

(22) Padia, F. N.; Yaseen, M.; Gore, B.; Rogers, S.; Bell, G.; Lu, J. R. Influence of Molecular Structure on the Size, Shape, and Nanostructure of Nonionic CnEm Surfactant Micelles. *J. Phys. Chem. B* **2014**, *118* (1), 179–188. https://doi.org/10.1021/jp409808c.

(23) Bernheim-Groswasser, A.; Wachtel, E.; Talmon, Y. Micellar Growth, Network Formation, and Criticality in Aqueous Solutions of the Nonionic Surfactant C12E5. *Langmuir* **2000**, *16* (9), 4131–4140. https://doi.org/10.1021/la991231q.

(24) Creatto, E. J.; Okasaki, F. B.; Cardoso, M. B.; Sabadini, E. Wormlike Micelles of CTAB with Phenols and with the Corresponding Phenolate Derivatives - When Hydrophobicity and

Charge Drive the Coacervation. *Journal of Colloid and Interface Science* **2022**, *627*, 355–366. https://doi.org/10.1016/j.jcis.2022.07.044.

(25) Strey, R.; Glatter, O.; Schubert, K. -V.; Kaler, E. W. Small-angle Neutron Scattering of D2O–C12E5 Mixtures and Microemulsions with N-octane: Direct Analysis by Fourier Transformation. *J. Chem. Phys.* **1996**, *105* (3), 1175–1188. https://doi.org/10.1063/1.471960.

(26) Xiao, H.; Choi, S. R.; Zhao, R.; Ploessl, K.; Alexoff, D.; Zhu, L.; Zha, Z.; Kung, H. F. A New Highly Deuterated [18F]AV-45, [18F]D15FSP, for Imaging β-Amyloid Plaques in the Brain. *ACS Med. Chem. Lett.* **2021**, *12* (7), 1086–1092. https://doi.org/10.1021/acsmedchemlett.1c00062.

(27) Sheepwash, E. E.; Rowntree, P. A.; Schwan, A. L. The Preparation of Three New Partially Deuterated Hexadecanethiols for Applications in Surface Chemistry. *J. Label. Compd. Radiopharm*. **2008**, *51* (12), 391–398. https://doi.org/10.1002/jlcr.1541. Notably, dododecanol-$d_{25(90\%)}$ was used instead of dododecanol-d_8 in the synthesis.

(28) Rosen, M. J. *Surfactants and Interfacial Phenomena*, 3rd ed.; Wiley-Interscience; John Wiley & Sons, Inc: Hoboken, New Jersey, USA, 2004.
